# Biogenesis of Pro-senescent Microparticles by Endothelial Colony Forming Cells from Premature Neonates is driven by SIRT1-Dependent Epigenetic Regulation of MKK6

**DOI:** 10.1038/s41598-017-08883-1

**Published:** 2017-08-15

**Authors:** Stéphanie Simoncini, Anne-Line Chateau, Stéphane Robert, Dilyana Todorova, Catherine Yzydorzick, Romaric Lacroix, Isabelle Ligi, Laurence Louis, Richard Bachelier, Umberto Simeoni, Frédérique Magdinier, Françoise Dignat-George, Florence Sabatier

**Affiliations:** 1 Aix Marseille Univ, INSERM, VRCM, Marseille, France; 2 Service de pédiatrie, Université de Lausanne, CHUV, 1011 Lausanne, Suisse Switzerland; 3 APHM, CHU de la Conception, Département de Néonatologie, Marseille, France; 4 Aix Marseille Univ, INSERM, GMGF, Marseille, France; 5 APHM, CHU de la Conception, Service d’hématologie, Marseille, France; 6 APHM, CHU de la Conception, Laboratoire de culture et thérapie cellulaire, INSERM, CBT-1409 Marseille, France

## Abstract

Senescent cells may exert detrimental effect on microenvironment through the secretion of soluble factors and the release of extracellular vesicles, such as microparticles, key actors in ageing and cardiovascular diseases. We previously reported that sirtuin-1 (SIRT1) deficiency drives accelerated senescence and dysfunction of endothelial colony-forming cells (ECFC) in PT neonates. Because preterm birth (PT) increases the risk for cardiovascular diseases during neonatal period as well as at adulthood, we hypothesized that SIRT1 deficiency could control the biogenesis of microparticles as part of a senescence–associated secretory phenotype (SASP) of PT-ECFC and investigated the related molecular mechanisms. Compared to control ECFC, PT-ECFC displayed a SASP associated with increased release of endothelial microparticles (EMP), mediating a paracrine induction of senescence in naïve endothelial cells. SIRT1 level inversely correlated with EMP release and drives PT-ECFC vesiculation. Global transcriptomic analysis revealed changes in stress response pathways, specifically the MAPK pathway. We delineate a new epigenetic mechanism by which SIRT1 deficiency regulates MKK6/p38^MAPK^/Hsp27 pathway to promote EMP biogenesis in senescent ECFC. These findings deepen our understanding of the role of ECFC senescence in the disruption of endothelial homeostasis and provide potential new targets towards the control of cardiovascular risk in individuals born preterm.

## Introduction

Preterm (PT) birth is associated with an increased risk of vascular-related diseases during the neonatal period, such as bronchopulmonary dysplasia and retinopathy, as well as early in adulthood, including hypertension and emphysema^1–4^. Adverse early programming may explain such association and influence cardiovascular risk over the life course by acting on the vascular bed. Vascular alterations are major determinants and include an increase in arterial stiffness, a reduction in microvascular density because of incomplete vasculogenesis, and an impairment of endothelial function^5, 6^.

Endothelial Colony Forming Cells (ECFC) are a relevant subset of endothelial progenitor cells that mainly reside in vessel wall, circulate at low frequency in the blood, and support vascular repair and *de novo* vessel formation^7–9^. A growing body of evidence supports their role as potential biomarkers and therapeutic targets in PT birth^5, 10, 11^. Among the factors that can alter cord blood ECFC functions in premature neonates, we recently demonstrated the involvement of accelerated senescence driven by sirtuin-1 (SIRT1) deficiency, which determines their vasculogenic defects^12^. Senescence of ECFC, limiting the ability for vascular healing, has emerged as an important contributor to the disruption of endothelial integrity developing under cardiovascular risk factors^8, 13, 14^.

In addition to the loss of specialized functions of the cells themselves, the detrimental effect of cells displaying stress-induced premature senescence (SIPS) might be due to an altered secretory phenotype referred to as senescence-associated secretory phenotype (SASP). According to the cell type and senescence context text^15^, SASP components such as cytokines, proteases, and extracellular vesicles (EV)^16, 17^ modulate the behavior of neighboring cells in a paracrine and/or autocrine manner, alter the cellular microenvironment, and can contribute to the functional decline of tissues and the progression of aging-related diseases^18–20^. This concept is mainly based on *in vitro* studies of fibroblasts undergoing excessive replication, but to our knowledge no *ex vivo* studies have characterized endothelial SAPS in the context of SIPS associated to cardiovascular risk factors.

Moreover, among the SASP components, previous studies have mainly focused on secreted soluble factors. However, endothelial cells, like most cells, release different types of extracellular vesicles EV, including microparticles. Microparticles are small heterogeneous membranous structures (0.1–1 µm) released under normal or pathological conditions, which can mediate phenotypic modification and reprogramming of cell function, in their local environment or at remarkable distance from their site of origin^21^. Circulating endothelial microparticles (EMP) are increased in conditions of vascular stress or dysfunction and have been demonstrated to behave not only as markers of endothelium damages^22^, but also as effectors in the control of vascular homeostasis^23^. EMP display variety of phenotypic and functional characteristics highly dependent on the functional state of the releasing cells and the mechanisms underlying the vesiculation process.

Despite the clinical importance of EMP in ageing and cardiovascular diseases, little is known about the mechanisms linking SIPS and EMP biogenesis. Herein, using senescent ECFC isolated from cord blood of PT neonates, we aimed to investigate whether SIRT1 deficiency could activate signaling pathways inducing EMP formation. Our study evidenced that PT-ECFC acquire a specific SASP including EMP able to propagate endothelial senescence. Moreover, we delineate a new epigenetic mechanism by which SIRT1 deficiency regulates MKK6/p38^MAPK^ pathway to promote EMP biogenesis in senescent ECFC.

## Results

### PT-ECFC displayed a senescence-associated secretory phenotype

**Figure 1:**
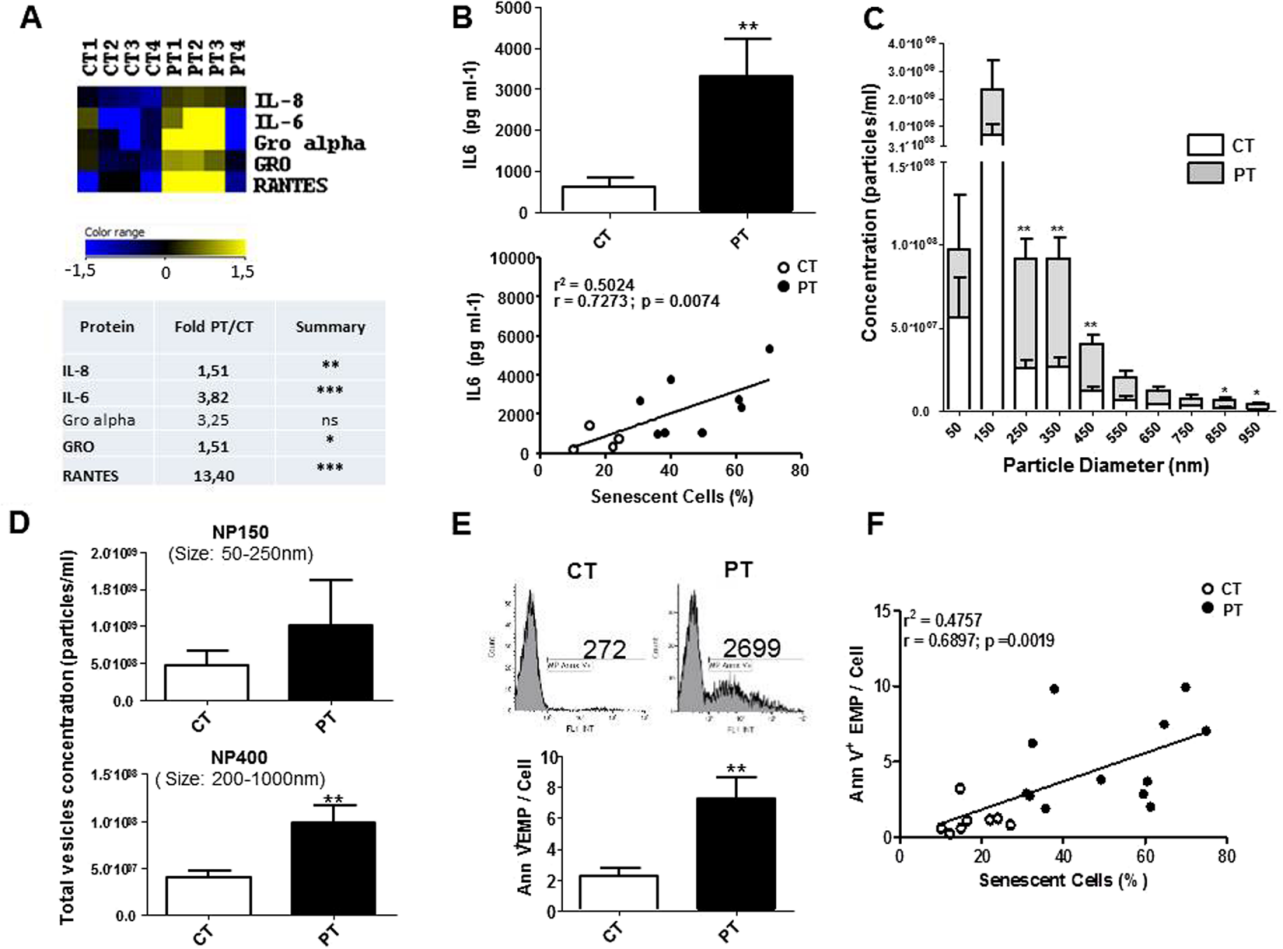
Prematurity modulates a subset of SASP. (**A**) Analysis of factors secreted by CT- and PT-ECFC using antibody arrays. CT-conditioned media signals were used as the baseline. The heat maps key represents log2-fold changes of PT-media from the baseline. Signals higher and lower than the baseline are highlighted in yellows and blue respectively. Factors that differed significantly between the CT- and PT-media are represented by an asterisk (*). (**B**) An ELISA assay confirmed that IL6 levels were significantly increased in conditioned media from PT vs. CT cells (upper panel, N = 9 vs. 5). Correlation between the secreted IL6 levels and the senescence percentage of ECFC (lower panel). (**C**) The qNano profiles of the concentrations of unfractionated raw vesicles for CT and PT-media using NP150 and NP400 nanopores (N = 5 vs. 6). (**D**) Size distribution of endothelial vesicles from CT- and PT-media, as estimated by qNano. The figure depicts the diameter of the vesicles (in nm) versus the normalized concentration of vesicles (in particles/ml). 60-nm bin size. (**E**) Comparison of levels of Annexin-V^+^ EMP of CT- and PT-media (N = 11 vs. 16). Representative plots and quantification of EMP measurements by flow cytometry (size of 0.1–1 µm that stained positively for Annexin-V). (**F**) Correlation between Annexin-V^+^ EMP levels and the senescence percentage of ECFC (N = 20). The data for all the bar graphs are presented as the means ± SEM. (Statistical analyses: Mann Whitney, Unpaired t-test; *p < 0.05, **p < 0.01, ***p < 0.001).

To determine whether PT-induced senescence of ECFC could induce a SASP, we first assessed the secretion profiles of ECFC from preterm neonates (PT-ECFC) and control ECFC (CT-ECFC) using cytokine antibody arrays (Figure S1A). After 48 h of basal culture, higher levels of cytokines (notably IL6, IL8, GRO and RANTES, previously identified as SASP components^24–26^ were detected in the conditioned media from PT-ECFC compared CT-ECFC (Fig. 1A, Figure S1B,C). Using an ELISA, we confirmed the enhanced secretion of IL6, a highly conserved SASP component shown previously elevated in endothelial cells at replicative senescence^27^, by PT-ECFC (Fig. 1B, upper panel). Strikingly, the IL6 level positively correlated with the percentage of senescent cells (Fig. 1B, lower panel) and inversely correlated with gestational age (Figure S2A).

We then used the qNano system to investigate whether this secretory profile was accompanied by a modulation in EV release. The size distribution of the unfractionated EV ranged from approximately 50 to 1,000 nm in diameter, indicating the presence of a mixed population of exosomes (≤100 nm) and EMP (0.1 to 1 µm) (Fig. 1C). No significant change in exosomes concentration was observed (Fig. 1D, upper panel), while the media from PT-ECFC exhibited significantly higher concentrations of particles greater than 200 nm compared to that of CT- ECFC (approximately 2.5-fold increase; Fig. 1D, lower panel and 1C), indicating enhanced EMP production.

Consistently, flow cytometry analysis of the PT-conditioned media confirmed the higher levels of EMP, defined as Annexin V+events, with size ranging from 0.1 to 1 µm, compared to the CT group (Fig. 1E). EMP release positively correlated with the percentage of senescent cells (Fig. 1F) and inversely correlated with gestational age (Figure S2B). We also observed a significant correlation between EMP release and IL6 secretion, linking the cytokine secretory profile with EMP biogenesis in senescent PT-ECFC (Figure S2C). Altogether, these data indicate that in the context of SIPS, PT-ECFC display a SASP associated with increased EMP release.

### EMP from PT-ECFC mediate a paracrine induction of senescence in endothelial cells

**Figure 2:**
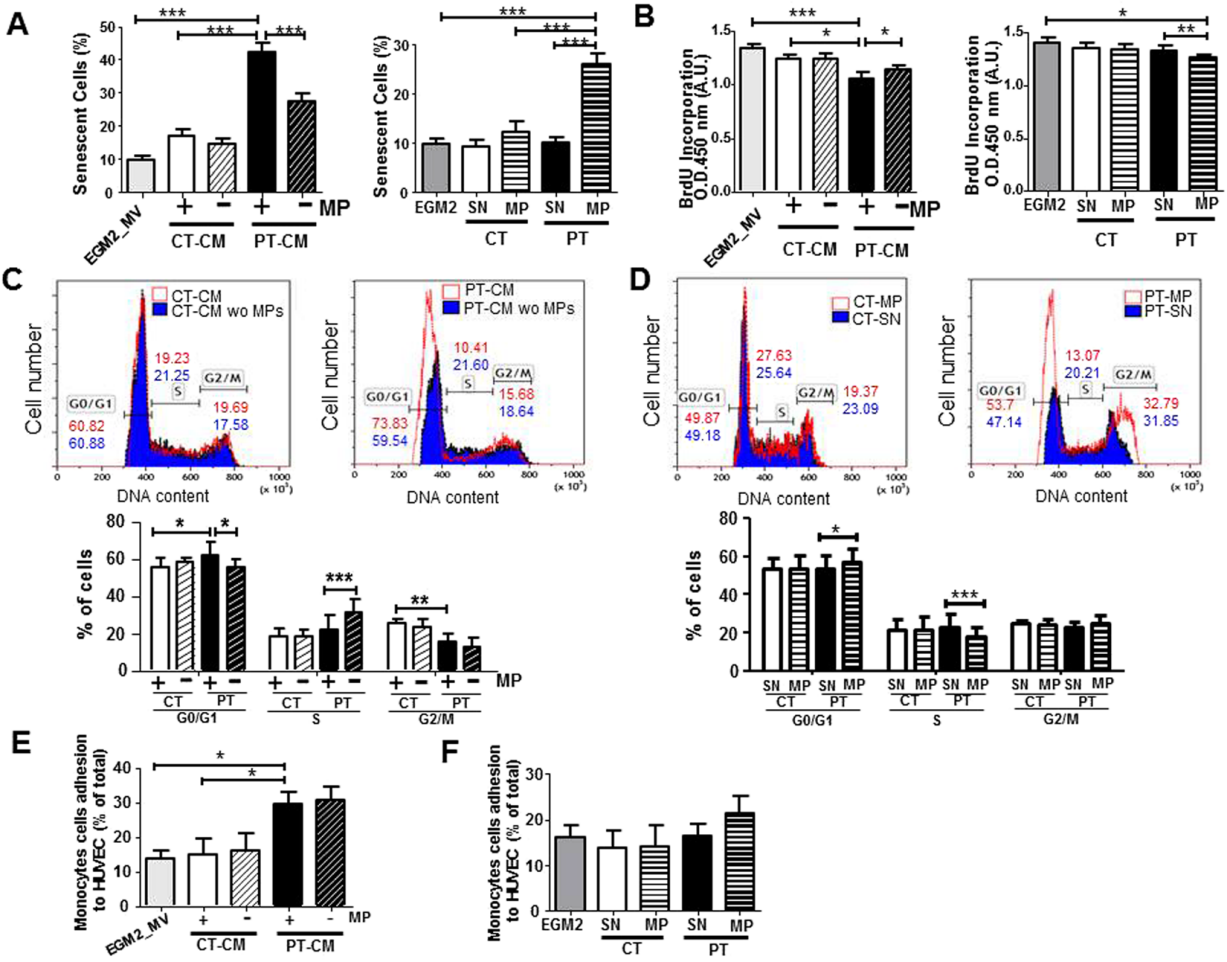
EMP components of the PT-SASP are key mediators of paracrine senescence. (**A**) Quantification of positive senescence-associated (SA) β-galactosidase staining in HUVEC exposed for 48 h to conditioned media (CM) ± depleted in EMP (left) or vehicle (SN), CT-EMP or PT-EMP (right). (HUVEC, N = 4; N = 6 CT vs. 7 PT). (**B**) Proliferation was assessed in a BrdU incorporation assay. The impact of CM ± depleted in EMP (left). Impact of purified EMP (right). (HUVEC, N = 4; N = 6 CT vs. 7–9 PT). (**C,D**) Cell cycle distribution by flow cytometry. DNA content in each cell cycle phase in HUVEC was analyzed by flow cytometry after propidium iodide staining. Representative histograms (upper panel) are from HUVEC treated with CM ± depleted in EMP **(C)** or purified EMP from CT- and PT-ECFC **(D)**. Graph (lower panel) represents a mean percentage of cells at different phases of the cell cycle determined by the DNA content ± SD for 4 independent HUVEC treated with 6 CT vs 7–9 PT samples. *p < 0.05; **p < 0.01; ***p < 0.001 (**E,F**) THP1 monocytic cell line adhesion assay on HUVEC exposed overnight to (**E**) CM ± depleted in EMP or (**F**) vehicle (SN), CT-EMP or PT-EMP. Data are represented as means ± SEM for 4 independent HUVEC treated with 4 CT vs 7 PT independent samples. Each experiment was carried out in triplicate. *p < 0.05.

We performed *in vitro* assays to investigate the senescence-induction capacity of the EMP part of the conditioned media from CT- or PT-ECFC. Early-passage HUVEC were used as target cells to exclude the confounding effects of replicative senescence. HUVEC exposed to CT-media for 48 h grew normally, whereas those treated with PT-media displayed higher levels of positive staining for SA-β-galactosidase (Fig. 2A left panel; Figure S3A), associated with a significant reduction in BrdU incorporation (Fig. 2B left panel). Cell cycle distribution analysis revealed drastic cell cycle arrest at the G0/G1 phase for HUVEC exposed to PT-media (Fig. 2C). Interestingly, EMP depletion of the PT-media, as determined by the qNano system (Figure S4), significantly attenuated the deleterious effects of the total media on the senescent phenotype and mitotic potential of HUVEC. Thus, the paracrine pro-senescent effect of PT-conditioned media might involve, at least in part, the EMP fraction.

To sustain the role of EMP, HUVEC were also treated with an adjusted concentration of CT-EMP or PT-EMP purified from the conditioned media. Twenty-four hours after treatment, PT-EMP significantly increased the SA-β-galactosidase activity and senescence-associated morphological changes in HUVEC compared to treatment with CT-EMP or vehicle (Fig. 2A right panel, Figure S3B). In addition, HUVEC exposed to PT-EMP displayed a significant increase in the level of the senescence-associated p21 protein, whereas the p16 level was unchanged (Figure S3C). Consistent with the senescent phenotype, PT-EMP-treated cells also exhibited significantly reduced proliferation, with a marked increase in G0/G1 (Fig. 2B right panel and 2D).

To test whether the EMP fraction from PT-ECFC could also contribute to the SASP pro-inflammatory potential, we evaluated if the pre-treatment of HUVEC with total or EMP-depleted conditioned media and purified EMP or the corresponding vehicle affected monocyte adhesion. A significant increase in the adhesion of calcein-AM-labeled monocytes was observed when HUVEC were co-incubated with PT-conditioned media, but not with CT-conditioned media (Fig. 2E). However, EMP depletion did not significantly prevent the pro-adhesive effect of PT-conditioned media. Consistently, monocyte adhesion to HUVEC was only slightly affected by purified PT-EMP compared to CT-EMP and the vehicle (Fig. 2F). Taken together, these data indicated that the EMP play a prominent paracrine role in senescence induction rather than in the pro-inflammatory response orchestrated by the SASP of PT-ECFC.

### SIRT1 level drives PT-ECFC vesiculation

**Figure 3:**
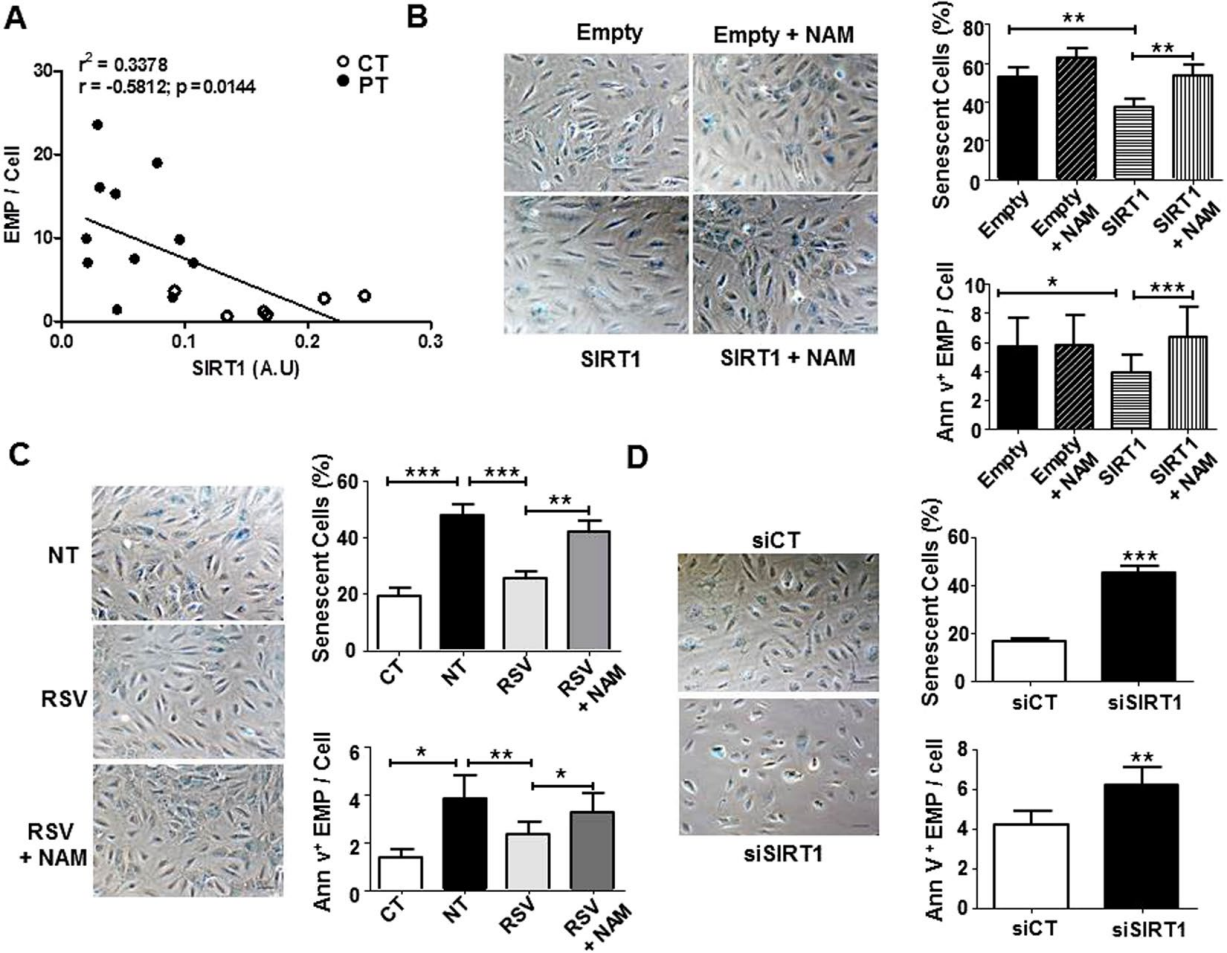
SIRT1 defect drives the senescence-associated EMP release induced by prematurity. (**A**) Correlation between Annexin-V+EMP release and SIRT1 protein levels. Every dot represents a single sample collected from term or PT neonates (N = 17). (**B–D**) Representative images and the quantification of ECFC positive for SA β-galactosidase activity (original magnification ×20, scale bar, 49 µm) and the quantification of EMP release by flow cytometry using Annexin-V staining. (**B**) The effect of SIRT1 overexpression on PT-ECFC (48 h, N = 14). PT-ECFC were transiently transfected with pCMV-sport6 vector (Empty) or the pCMV-Sport6-SIRT1vector (SIRT1), and treated or not with nicotinamide (NAM, 1 mM). (**C**) The effect of SIRT1 modulation by resveratrol (RSV) treatment on PT-ECFC (48 h, N = 18, CT = 9). PT-ECFC were treated with solvent alone (NT) or 1 µM of resveratrol (RSV, in the presence or absence of 1 mM of NAM (**D**) The effect of SIRT1 silencing on CT-ECFC (48 h, N = 11). CT-ECFC were transiently transfected with control siRNA (siCT) or SIRT1 siRNA (siSIRT1). The data for all of the bar graphs are presented as the means ± SEM. (Statistical analysis: One-way ANOVA, t-test; *p < 0.05, **p < 0.01, ***p < 0.001).

Our previous study showed that SIRT1 defect drives accelerated PT-ECFC senescence^12^. Thus, we asked whether SIRT1 might control PT-ECFC vesiculation. Consistently, we found a significant inverse correlation between EMP release and the SIRT1 protein level in ECFC (Fig. 3A). In addition, 48 h after transient transfection of PT-ECFC with a SIRT1-encoding vector, we observed as expected a significantly reduced SA-β-gal activity and senescence-associated morphological changes compared to cells transfected with an empty vector (Fig. 3B upper panel). Interestingly, SIRT1 overexpression was associated with a significant decrease in the level of released EMP (Fig. 3B lower panel). Moreover, the addition of nicotinamide (NAM), a well-established inhibitor of SIRT1 activity^28^, counteracted the effects of SIRT1 overexpression on EMP release and PT-ECFC senescence phenotype (Fig. 3B). Because we previously observed that resveratrol (RSV) reverses PT-ECFC senescence by inducing SIRT1 expression^12^, we tested whether RSV could also prevent EMP release in addition to senescence onset. As expected, 48 h of treatment with 1 μM RSV reduced the percentage of senescent PT-ECFC (Fig. 3C upper panel). In addition, RSV treatment significantly reduced EMP release by PT-ECFC to a level close to that of CT-ECFC, and this effect was abrogated by NAM (Fig. 3C lower panel), suggesting that RSV inhibits senescence-associated EMP release by PT-ECFC in a SIRT1-dependent manner.

To further uncover the causal relationship between SIRT1 downregulation and enhanced endothelial vesiculation, we analyzed the consequences of siRNA-mediated SIRT1 knockdown at both the mRNA and protein levels in CT-ECFC (Figure S5A,B). A significant increase in SA-β-gal activity was observed (Fig. 3D upper panel), whereas no apoptotic signal was detected using Annexin-V/7-AAD staining (Figure S5C). Growth arrest of senescent CT-ECFC, attested by a marked increase in p16Ink4 expression, in the absence of modulation of the p53/p21WAF pathways suggested that SIRT1 knockdown in CT-ECFC mimics the previously described^12^ PT-ECFC senescence phenotype (Figure S5A-B). Decreased SIRT1 expression in CT-ECFC was accompanied by the significant upregulation of EMP release (Fig. 3D lower panel). Altogether, these data indicate that reduced SIRT1 level drives the senescence-associated release of EMP by PT-ECFC.

### Microarray Analysis reveals an upregulation of the MKK6-p38^MAPK^ pathways in PT-ECFC

**Figure 4:**
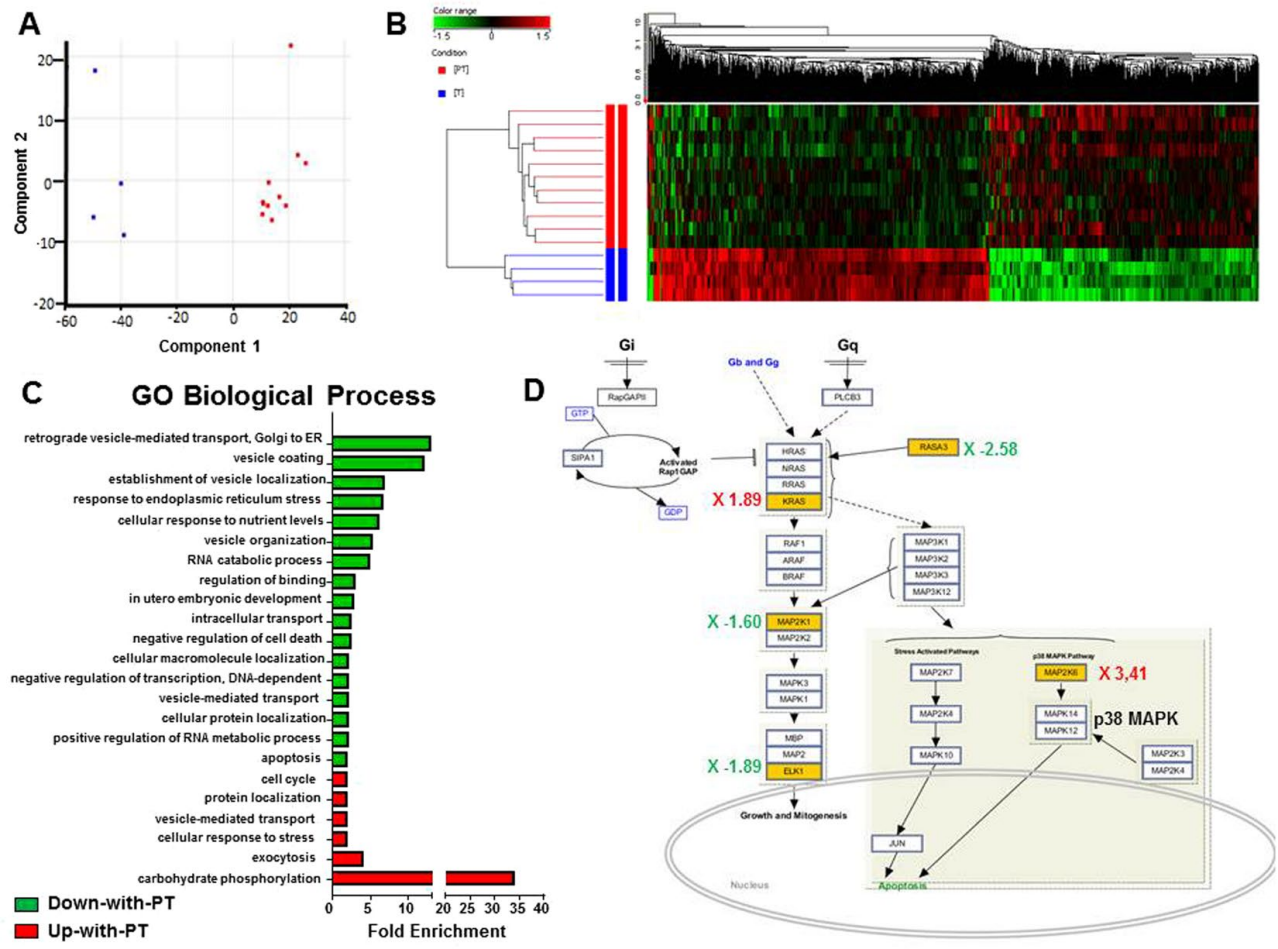
Prematurity induces a distinct transcriptome profile in ECFC. (**A**) Principal component analysis (PCA) plot of the gene expression profiles across 4 CT (blue) and 11 PT-ECFC (red). Each point represents a single ECFC sample, whereas the distance between points is indicative of the global gene expression similarity, such that similar array profiles are positioned closer together. (**B**) The hierarchical clustering for differentially expressed genes detected by whole-genome microarray analysis of the mRNA obtained from CT- and PT-ECFC. Genes in red: increased expression; genes in green: decreased expression. (**C**) Fold enrichment over chance for the Gene Ontology (GO) biological process of the Up-with-PT (red) or Down-with-PT (green) gene lists using DAVID (fold change ≥ 2, p ≤ 0.001). (**D**) The MAPK pathway in PT-ECFC. Pathway analysis was performed using SEA GeneSpring resources; differentially expressed genes are shown (yellow boxes, p ≤ 0.05), with the induction ratio derived from the microarray data.

To explore the molecular mechanisms underlying the SIRT1-dependent EMP production, we performed global gene expression profiling. As shown in Fig. 4A, after array normalization, the gene expression profiles of CT- and PT-ECFC were reduced to 2 principal components, demonstrating that these cells had distinct profiles. Subsequently, 41,087 probes that passed our quality control threshold were assessed for differential expression. Overall, hierarchical clustering identified 973 probes that were differentially regulated between CT and PT-ECFC. Of these probes, 439 (253 genes) and 534 (476 genes) were up- and downregulated respectively, in PT-ECFC (≥1.5 fold-change, p ≤ 0.05; Fig. 4B, Tables S2–3).

To provide a cohesive view of the biological functions and pathways associated with the expression changes of the PT genes, we integrated multiple resources. DAVID was used to determine which biological process was over-represented. The upregulated genes had a strong association with stress response, negative regulation of proliferation and cell cycle, protein localization and exocytosis, whereas the categories enriched for the downregulated genes were associated with cell growth and preservation of cell integrity, such as cell development, proliferation and apoptotic regulation (Fig. 4C, Tables S4–5). In addition, we performed pathway analysis to predict the transcriptional networks using the Finding Significant Pathway tools in GeneSpring GX. This analysis revealed multiple enriched pathways, including the mitogen-activated protein kinase (MAPK), Wnt, insulin and DNA damage signaling pathways (Table S6). Collectively, these analyses identified an enrichment of genes involved in the MAPK pathways (Fig. 4D), which were previously implicated in both stress response, senescence and cytoskeleton reorganization^19, 29, 30^.

### SIRT1 deficiency leads to increased senescence and vesiculation through the p38^MAPK^ pathway activation in PT-ECFC

**Figure 5:**
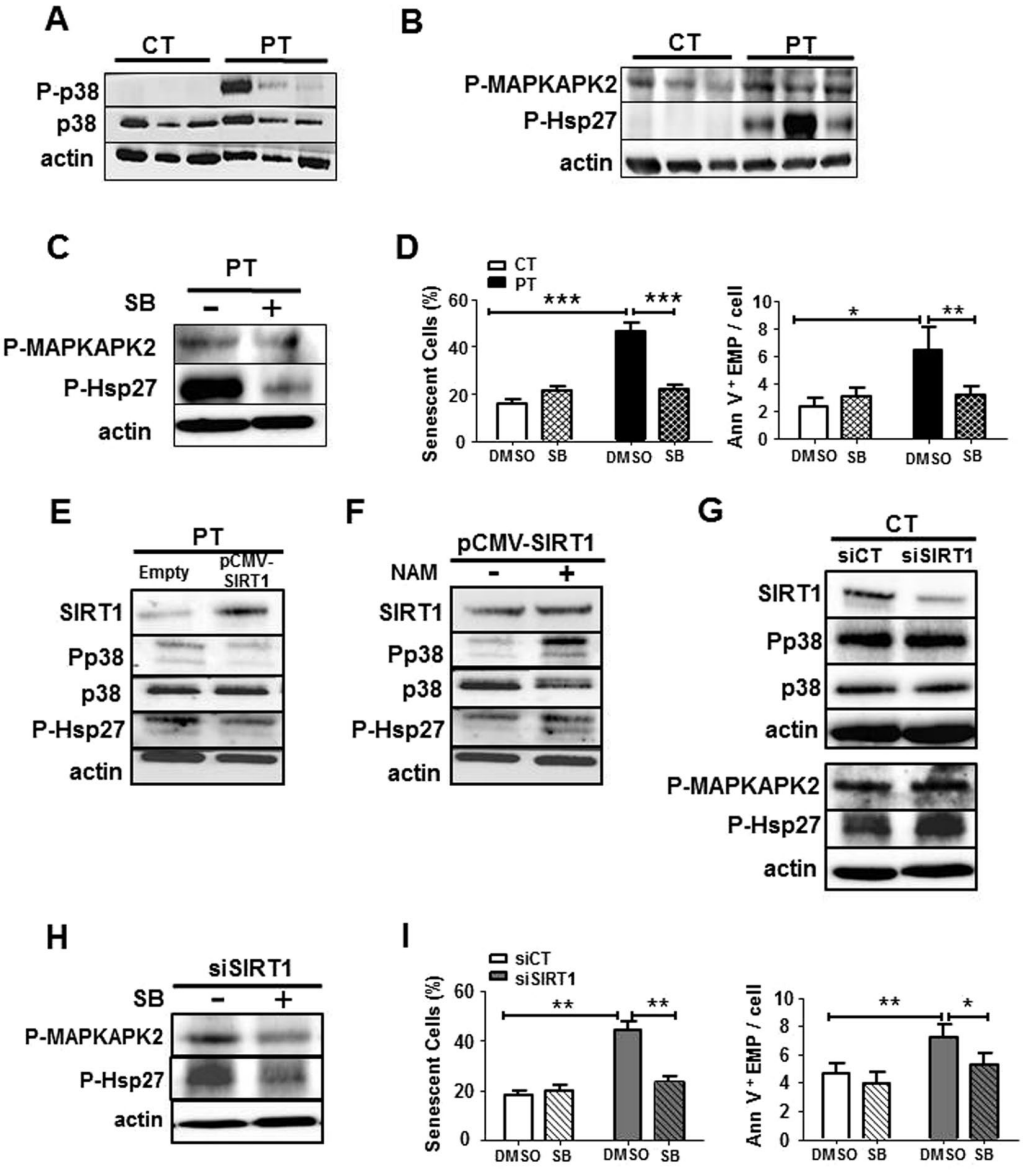
SIRT1 protects against senescence and vesiculation through the downregulation of the p38^MAPK^ pathway activation in ECFC. (**A**) The level of P-p38 and total p38 were examined through Western blot analyses in CT- and PT-ECFC (N = 11 vs 18). (**B**) Western blot analysis of p38^MAPK^ activation in CT- and PT-ECFC was performed by the determination of phosphorylation of MAPKAPK2 and Hsp27 (N = 11–18 vs 13–28) (**C**) Western blot analysis was performed to determine the levels of P_MAPKAPK2 and P-Hsp27 in PT-ECFC treated with 2 µM of the p38^MAPK^ activity inhibitor, SB203580 (SB). (**D**) The effect of SB203580 treatment on CT- and PT-ECFC (2 µM, 48 h, N = 6 vs. 14) on senescence and EMP release. Left panel: quantification of ECFs positive for SA β-galactosidase activity. Right panel: quantification of EMP release by flow cytometry using Annexin-V staining. (**E**) The impact of SIRT1 overexpression on p38^MAPK^ pathway activation in PT-ECFC (relative expression to empty; N = 8–11). (**F**) The impact of NAM on p38MAPK pathway activation (relative expression to SIRT1-transfected cells; N = 8–11) in PT-ECFC overexpressing SIRT1. (**G**) The impact of SIRT1 silencing on p38^MAPK^ pathway activation in CT-ECFC (relative expression to scrambled; N = 6–13). (**H,I**) The effect of SB203580 treatment on SIRT1-knockdown CT-ECFC was assessed on downstream effectors of p38^MAPK^ **(H**, **N = 4–7)**, as well as the percentage of ECFC positive for SA β-galactosidase activity **(I**, Left panel, **N = 8)** and EMP release **(I**, Right panel). The data for all of the bar graphs are presented as the means ± SEM. (Statistical analysis: One-way ANOVA, *p < 0.05, **p < 0.01, ***p < 0.001). All blots correspond to representative images of blot bands.

We then focused on the p38^MAPK^ pathways and first evidenced that in PT-ECFC, low SIRT1 expression was accompanied by a significant activation of the p38^MAPK^ pathway, as evidenced by increased phosphorylation of p38^MAPK^, without alteration of the total p38^MAPK^ level (Fig. 5A). We further investigated the downstream MAPKAPK2/Hsp27 signaling to p38^MAPK^, which was reported to play a key role in actin cytoskeleton remodeling and blebs formation^31, 32^. We observed significant increase in the phosphorylation of both downstream signaling molecules MAPKAPK2 and Hsp27 (Fig. 5B) in PT-ECFC compared to CT-ECFC. Interestingly, we previously reported the upregulation of another downstream target of the p38^MAPK^ pathway, the cyclin-dependent kinase inhibitor p16, in senescent PT-ECFC^12^. Application of SB203580, a p38^MAPK^ activity inhibitor^33^, significantly blocked the activation of MAPKAPK2/Hsp27 arm of p38^MAPK^ signaling and reduced EMP release from PT-ECFC (Fig. 5C and D right panel) compared to DMSO-treated PT-ECFC, these effects occurring concomitantly with the attenuation of the senescence phenotype (Fig. 5D left panel; Figure S6A). These data suggest that p38^MAPK^ via Hsp27 phosphorylation mediates senescence-associated release of EMP by PT-ECFC.

To investigate the relationship between SIRT1 and p38^MAPK^/MAKAPK2/Hsp27 pathways activation in the EMP release, we first evaluated the effects of SIRT1 overexpression using transient expression experiments and drug treatments in PT-ECFC. As shown in Fig. 5E, SIRT1 transfection into PT-ECFC significantly repressed p38^MAPK^ and Hsp27 activation compared with that of PT-ECFC transfected with an empty vector, while total p38^MAPK^ remain unchanged. Moreover, the addition of NAM counteracted the beneficial effect of SIRT1 overexpression on p38^MAPK^ pathway activation (Fig. 5F). Time course addition of RSV to PT-ECFC progressively induced a SIRT1 level increase associated with a gradual decrease in P-p38^MAPK^ and P-Hsp27 levels (Figure S7). Conversely, SIRT1 knockdown in CT-ECFC using SIRT1 siRNA transfection increased p38^MAPK^ activation and the phosphorylation of its target proteins compared to the control siRNA (Fig. 5G). Interestingly, inhibition of p38MAPK activity and MAPKAPK2 as well as Hsp27 phosphorylation with SB203580 partially blocked EMP release, concomitant to senescence, induced by SIRT1 knockdown in CT-ECFC (Fig. 5H,I, FigureS 8). Altogether these data indicate that SIRT1 defect, via the p38^MAPK^/MAPKAPK2/Hsp27 pathway activation, controls senescence-associated EMP release.

### SIRT1 deficiency leads to the upregulation of the p38^MAPK^ pathway by increasing MKK6 expression in PT-ECFC

**Figure 6:**
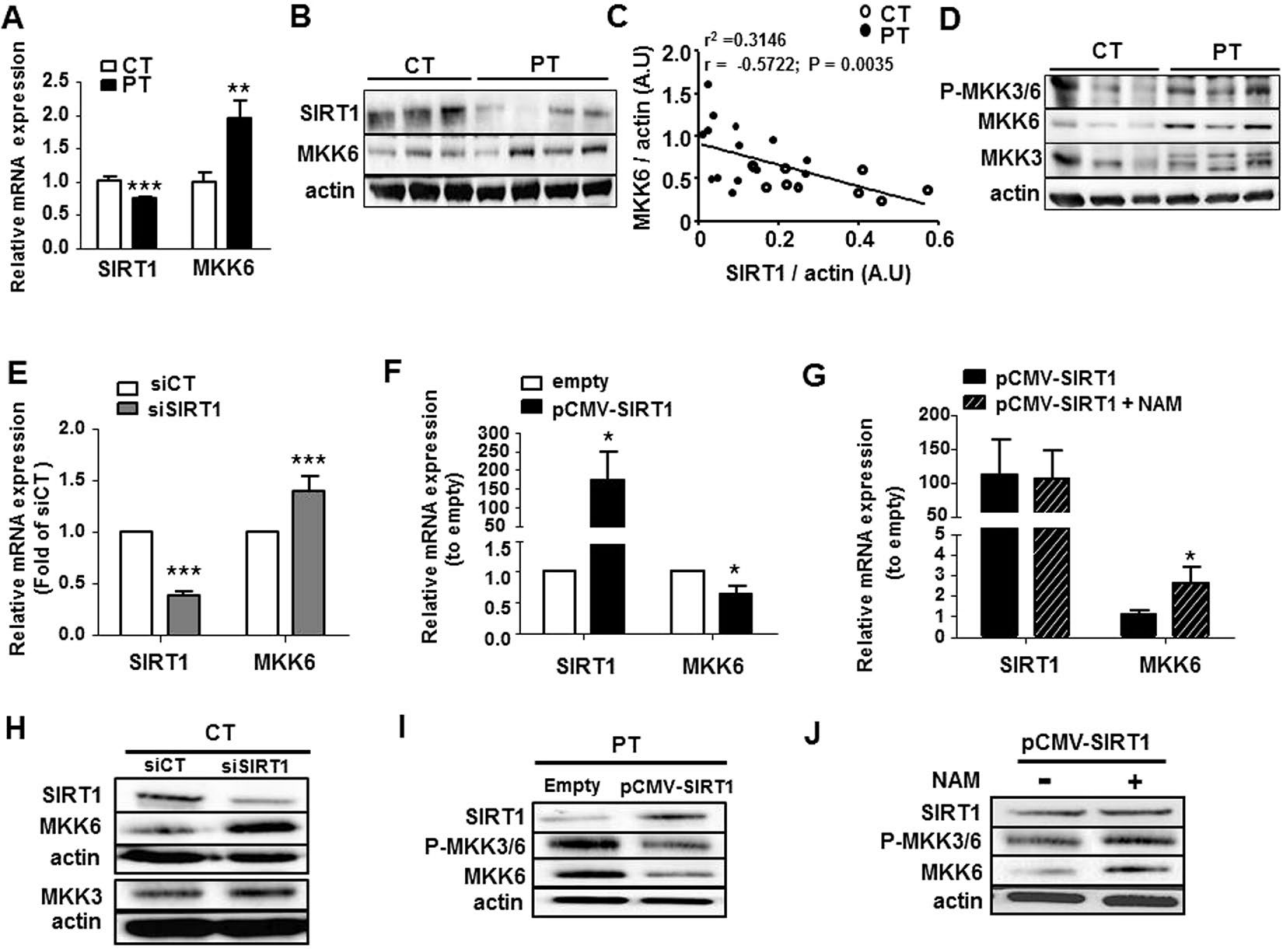
SIRT1 defect increased pMKK3/6 by increasing MKK6 levels. The SIRT1 and MKK6 mRNA **(A)** and protein **(B)** expression levels in CT-and PT-ECFC were assessed via RT-qPCR and Western blot analysis, respectively. (**A**) The impact of PT on the mRNA level (relative expression to CT; N = 13 CT vs. 16 PT). (**B**) The impact of PT on the protein level (relative expression to actin; N = 18 CT vs. 26 PT, representative blot). (**C**) The correlation between MKK6 and SIRT1 protein expression levels. Every plot represents a single sample collected from a patient (N = 24). (**D**) Western blot analysis of MKK3/6 activation in CT- and PT-ECFC was performed by the determination of phosphor-MKK3/6, MKK3 and MKK6 (N = 118 vs 28). (**E–J**) The impact of SIRT1 modulation on the activation of MKK3/6. SIRT1 and MKK6 mRNA **(E–G)** and protein **(H–J)** expression levels, along with MKK3/6 phosphorylation, were assessed via RT-qPCR and Western blot analysis, respectively. (**E**,**H**) The impact of SIRT1 silencing on the mRNA and protein expression of CT-ECFC (relative expression to scrambled; N = 12). (**F,I**) The impact of SIRT1 overexpression on mRNA and protein expression in PT-ECFC (relative expression to empty; N = 7 and 11, respectively). (**G,J**) The impact of NAM on mRNA (relative expression to empty, N = 8) and protein expression (relative expression to SIRT1-transfected cells; N = −6–9) in PT-ECFC overexpressing SIRT1. The data for all of the bar graphs are presented as the means ± SEM. (Statistical analysis: t-test; *p < 0.05, **p < 0.01, ***p < 0.001). All blots correspond to representative images of blot bands.

Having demonstrated the relationship between SIRT1 and p38^MAPK^ pathway activation in senescence-associated EMP release, we then examined the potential molecular intermediate. Phosphorylation of p38^MAPK^ is a downstream consequence of the activation of MKK3/6, upstream kinases, by phosphorylation (P-MKK3/6). Interestingly, in the above array analysis, marked differences were observed in the MKK6 (MAP2K6) expression level (Fig. 4D, Table S2). We therefore investigated whether SIRT1-dependent p38^MAPK^ pathway activation may occur through the modulation of MKK6 expression. Consistent with transcriptional profiling data, we first evidenced that in PT-ECFC, low SIRT1 expression and p38^MAPK^ pathway activation was accompanied by MKK6 upregulation at the mRNA and protein levels (Fig. 6A,B), and SIRT1 expression had a strong negative correlation with the MKK6 levels (Fig. 6C). Interestingly, both P-MKK3/6 and MKK6 were upregulated in PT-ECFC compared to CT-ECFC, while there was a decrease in MKK3 expression among the group. (Fig. 6D). Consistently, SIRT1 knockdown in CT-ECFC directly enhanced the mRNA and protein levels of MKK6 (Fig. 6E,H), whereas MKK3 level was not change (Fig. 6H). By contrast, as shown in Fig. 6F and I, SIRT1 transfection into PT-ECFC was associated with significant decreases in the mRNA and protein levels of MKK6 as well as MKK3/6 phosphorylation (Fig. 6I). The addition of NAM counteracted the beneficial effect of SIRT1 overexpression on MKK6 expression (Fig. 6G,J) and MKK3/6 activation (Fig. 6J). Similarly, the incubation of PT-ECFC with RSV resulted in the downregulation of MKK6, both at the mRNA and protein levels, concomitant to phosphorylation, along with p38^MAPK^ and its effector activation in a time-dependent manner (Figure S7), suggesting that SIRT1 defect-induced upregulation of MKK6 may occur prior to the upregulation of P-MKK3/6. Collectively, these results indicate that the activation of the MKK6/p38^MAPK^ pathway is, in part, regulated by SIRT1 through SIRT1-dependent MKK6 transcriptional modulation and participates in senescence-associated EMP release.

### SIRT1 represses MKK6 in an epigenetic manner

**Figure 7:**
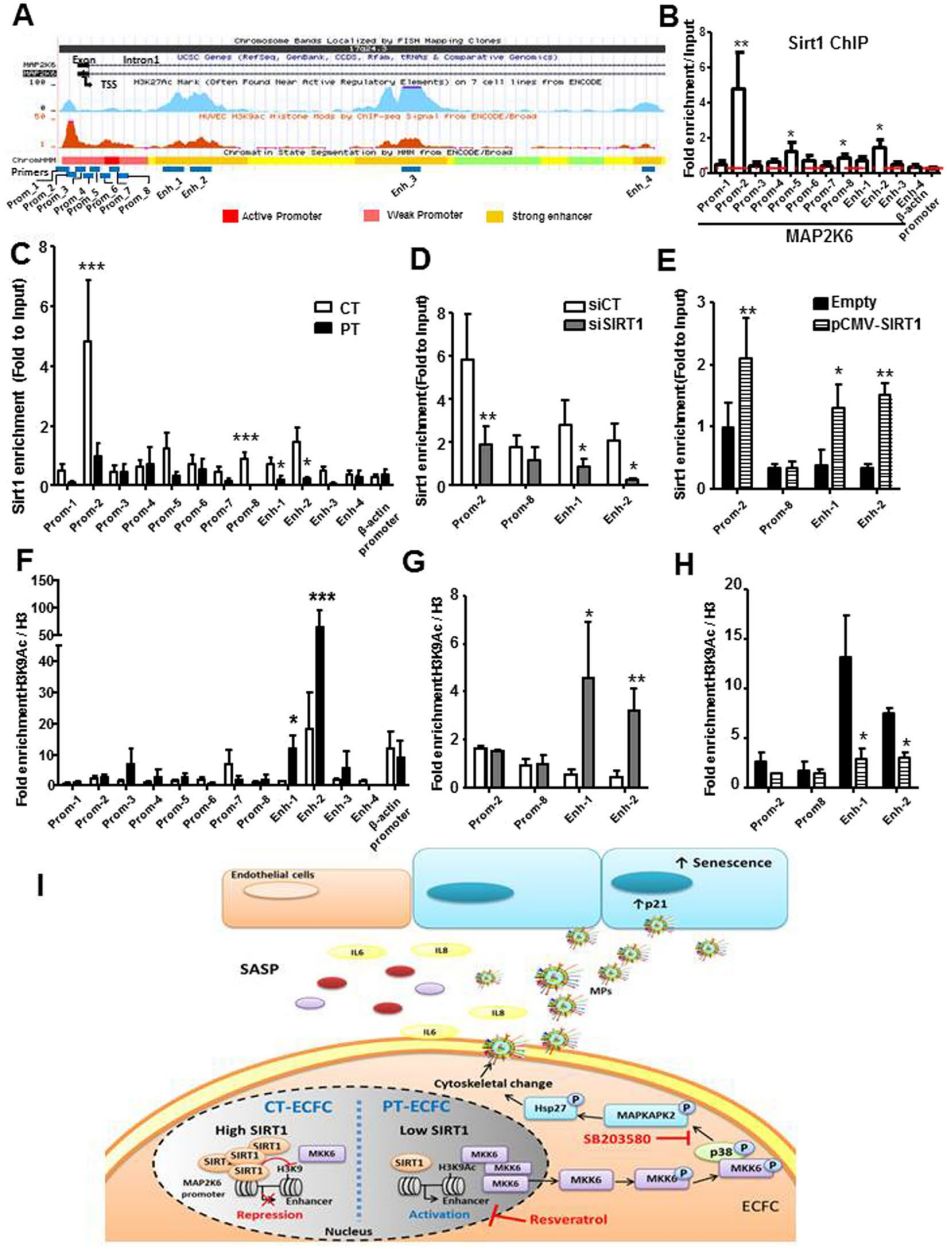
SIRT1 negatively regulates MKK6 transcription through epigenetic chromatin modification. (**A**) Genome browser view showing the genomic region surrounding the transcriptional start site (TSS, arrow) of human MAP2K6 (MKK6). Encode tracks illustrate the presence of H3K27ac and H3K9ac marks of the ChIP-seq and the promoter and enhancer regions in HUVEC. The annealing positions of primers used for the ChIP experiments are indicated at the bottom of the figure (**B**) ChIP assays were performed with chromatin prepared from CT-ECFC. The chromatin was immunoprecipitated with the antibody against SIRT1, and the precipitated genomic DNA was analyzed via RT-PCR using primers for different areas of the MAP2K6 promoter and enhancer region. The actin promoter served as a negative control. (**C** and **F**) ChIP assays were performed with chromatin prepared from CT- or PT-ECFC (N = 9 vs. 6). Chromatin was immunoprecipitated with the antibodies against SIRT1 **(C)**, H3 and H3K9Ac **(F)**. (**D** and **G**) ChIP assays were performed with CT-ECFC transfected with scrambled siRNA (siCT) or SIRT1 siRNA (siSIRT1) (N = 3–5). Chromatin was immunoprecipitated with antibodies against SIRT1 **(D)**, H3 and H3K9Ac **(G)**. The precipitated genomic DNA was analyzed via RT-qPCR using primers for the specific promoter (Prom_2 and 8) and the enhancer (Enh_1 and 2) regions. (**E** and **H**) ChIP assays of PT-ECFC transfected with pCMV (Empty) or pCMV-SIRT1 (N = 3). The chromatin was immunoprecipitated with antibodies against SIRT1 **(E)**, H3 and H3K9Ac **(H)**. The precipitated genomic DNA was analyzed via RT-qPCR using primers for the specific promoter (Prom_2 and 8) and enhancer (Enh_1 and 2) regions. The data for all of the bar graphs are presented as the means ± SEM. (Statistical analysis: Two-way ANOVA; *p < 0.05, **p < 0.01, ***p < 0.001). (**I**) Proposed signaling mechanism controlling the release of EMP and their functional impact in the SIPS context of prematurity.

To determine whether SIRT1 directly regulates the MAP2K6 gene, we performed chromatin immunoprecipitation (ChIP) assays. We selected 12 specific primer pairs encompassing the 5′ untranslated region (5′UTR) of the MAP2K6 gene, including the transcription start site (TSS), the enhancer sites within the first intron and the peaks enriched in histones H3K27ac and H3K9ac in HUVEC, described in the UCSC database^34^ (Fig. 7A). Within this 16,547-bp region, we found significant SIRT1 enrichment at the distal 5′ regulatory region (−437 to −329 bp) and in the first strong enhancer region of intron 1 (+3,005 to +3,196 bp) in CT-ECFC (Fig. 7B). Prematurity led to significantly decreased SIRT1 binding at these regions (Fig. 7C), suggesting that SIRT1 directly binds in proximity to the MAP2K6 TSS to regulate its transcription.

Specifically, increased H3K9ac, a mark of active chromatin and previously identified as a specific SIRT1 target^35^, was also enriched at the TSS and at the first strong enhancer region of PT-ECFC compared to that of CT-ECFC (Fig. 7F), suggesting that H3K9acplays a critical role in PT-induced MAP2K6 mRNA expression.

To further investigate whether SIRT1 regulates the H3K9Ac level on MAP2K6, we knocked-down SIRT1 using siRNA transfection in CT-ECFC or overexpressed it in PT-ECFC. SIRT1 silencing decreased SIRT1 binding to these regions and significantly increased the H3K9Ac level on the MAP2K6 enhancer (Fig. 7D and G). Conversely, in spite of SIRT1 binding to MAP2K6 regulatory regions, SIRT1 overexpression almost completely removed H3K9Ac at the first strong enhancer in PT-ECFC (Fig. 7E and H). Together, these results further indicate that SIRT1 binding might suppress MAP2K6 expression by modifying H3K9 acetylation at its first strong enhancer.

## Discussion

In the context of SIPS affecting PT-ECFC, we show that PT-ECFC develop a SASP characterized by increased levels of inflammatory cytokines and EMP able to propagate endothelial senescence. This study identifies a new epigenetic mechanism linking the SIRT1 and p38^MAPK^ pathways to control the biogenesis of EMP by senescent ECFC. We showed that SIRT1 deficiency induces a signaling pathway involving the sequential accumulation of active MMK6 and p38^MAPK^, which mediate EMP release. For the first time, MKK6 expression was shown to be negatively regulated by SIRT1 at the chromatin level, through deacetylation of histone H3 lysine 9 in its promoter region (Fig. 7I).

ECFC are attracting increasing interest in vascular disease modeling to probe mechanisms of endothelial pathogenesis and to delineate therapeutic targets, in particular in conditions associated to adverse intrauterine or perinatal environments such gestational diabetes, growth restriction or prematurity^10, 12, 36, 37^. Indeed, antenatal and postnatal stressors can significantly affect PT-ECFC, which are more vulnerable at this stage of development, corresponding to the third trimester of gestation^38^, compared to CT-ECFC^10, 12, 39^. Altered ECFC functions, mediated by disruption of proangiogenic pathway and accelerated senescence, may compromise the endothelial repair capacity with significant involvement in the progression of endothelial dysfunction^8, 13, 14, 26^ and development of subsequent diseases, such as bronchopulmonary dysplasia or retinopathy. Nevertheless, the spectrum of the paracrine effectors of senescent ECFC has never been fully investigated. Here, we demonstrated that PT-ECFC, presenting a SIRT1-dependent accelerated senescence^12^, established a SASP, as evidenced by the enhanced secretion of the typical pro-inflammatory cytokines, such as IL6, that correlated with the level of cell senescence and low gestational age. Our results are consistent with previous reports describing IL6 as the most predictive marker in cord blood of preterm birth^40^. Furthermore, we observed that the conditioned medium from PT-ECFC induces a proadhesive phenotype in non-senescent naïve endothelial cells, suggesting that the SASP of PT-ECFC may affect vascular health by actively participating in the low-grade proinflammatory status associated with prematurity.

In addition to soluble factors, increased release of EV from senescent cells constitutes a new mechanism for intercellular communication^16, 41^. The association of increased vesiculation and endothelial senescence has been recently described in *in vitro* models of replicatively senescent endothelial cells^42, 43^ but remains poorly characterized in *ex-vivo* endothelial models of SIPS. Interestingly, we showed that prematurity-associated senescence of ECFC leads to increased production of EMP, as identified by diameter size range and Annexin-V^+^ staining. In addition, PT-EMP levels correlate with senescence, gestational age and IL6 levels. Previous studies demonstrated that senescent cells promote paracrine senescence in culture models and in mouse models of oncogene-induced senescence *in vivo* ^20^. The novelty of our study is to identify the major contribution of the vesicular fraction of the secretome in mediating such effect. Indeed, purified EMP from PT-ECFC were able to implement a full senescence response in naïve HUVEC. Our findings are consistent with recent studies demonstrating that EMP contribute to vascular aging propagation^44, 45^. Together with our previous report of elevated EMP levels in cord blood of PT neonates^5^, these observations suggest that EMP are relevant participants of the ECFC-mediated SAPS occurring in the context of preterm birth. The molecular mechanisms by EMP from PT-ECFC can induce paracrine senescence require future investigation based on EMP profiling. Among the bioactive components conveyed by EMPs and able to act on nearby cells, miRNA deserve increasing attention. Indeed, miRNa packaged in EV can be transferred to target cells, exert gene silencing effect and play a role in a broad range of pathophysiological processes including senescence, as recently demonstrated for EV derived from bone cells, cancer cells or myotubes^46–48^. Another hypothesis may involve pro-oxidant message carried by EMP from senescent cells^43^. Nevertheless, the strongest paracrine pro-senescent effect was obtained for unprocessed PT-media and removing the EMP from the secretome did not completely abrogate the biological activity, suggesting the presence of both soluble and vesicular factors acting in concert to trigger paracrine senescence mechanisms. Some reports indicate that EV released upon inflammatory stimulation can contain cytokines such as members of IL-1 family or IL-6^49–51^. In our study, analysis of the proadhesive phenotype of HUVEC co-cultured with PT-EMP demonstrated that PT-EMP have not a prominent role in the pro-inflammatory response orchestrated by the SASP. This data may indicate that despite the relationship between EMP and IL-6 levels released by PT-ECFC, pro-senescent EMP do not carry biologically relevant amounts of IL-6. However, elevated levels of circulating microparticles are associated with a number of cardiovascular and inflammatory pathologies (e.g., hypertension, diabetes and metabolic syndrome), and microparticles are known to actively contribute to the pathogenesis of cardiovascular diseases, including inflammation^22, 52^. Thus, a potential role of senescence-associated EMP in low-grade and prolonged endothelial activation cannot be ruled out. In addition to their increased production levels, the qualitative differences in the bioactivities of EMP generated by senescent ECFC reinforce the notion that the multifaceted role of EMP are strongly dependent upon the cellular context associated with their formation.

Given the detrimental role of EMP on endothelial homeostasis, knowledge of the molecular mechanisms governing EMP biogenesis in senescent cells is challenging. SIRT1 possesses protective roles against vascular aging and resultant cardiovascular diseases^53^. Although the beneficial effect of SIRT1 on endothelial senescence has been extensively studied, its direct role in the regulation of SASP is poorly described. Recently, a role of SIRT1 deficiency in the regulation of SASP components, such as IL6, IL8 and plasminogen activator inhibitor (PAI) expression, has been described in fibroblastic cell lines and endothelial cells undergoing replicative senescene^54, 55^. Here, using strategies allowing the modulation of SIRT1 level in ECFC, we assigned to SIRT1 a critical role in the control of senescence-associated EMP biogenesis. This could be consistent with a recent observation showing that increased EMP levels accompanied a decreased mitochondrial biogenesis through a SIRT1-dependent mechanism^56^. While we do not exclude the involvement of alternative mechanisms impacting on EMP biogenesis, our results establish a new link between the deficiency of SIRT1 and EMP release involving MKK6/p38^MAPK^/Hsp27signaling pathway. The data presented here have focused on the activation of the p38^MAPK^ pathway and the role of specific upstream MAPK Kinase upon SIRT1 defect. Although both MKK3 and MKK6 are required for the p38^MAPK^ phosphorylation in response to environmental stresses, we observed that SIRT1 defect induces the activation of MKK3/6 through only an upregulation of MKK6, which participates to the activation of p38^MAPK^ and subsequently, to the occurrence of SIPS as well as senescence-associated EMP release. Our findings are in agreement with prior studies revealing p38^MAPK^ pathway as an upstream mediator in senescence and SASP induction^19, 57, 58^ as well as playing a major role in MP shedding^23, 30^. In endothelial cells, Hsp27 phosphorylation, mediated by p38MAPK signaling pathway, is thought to play an important role in actin cytoskeletal rearrangement, membrane blebbing, and EMP generation^31, 32^. Here, we demonstrate that selective activation of p38^MAPK^ in response to SIRT1 defect is required for Hsp27 phosphorylation and associated EMP formation. Together, these data suggest that the environmental stress of the perinatal period might modulate senescence and the secretory profile through the SIRT1/MKK6/p38^MAPK^-dependent pathways.

Importantly, the SIRT1 modulation experiments argue in favor of a primary role for SIRT1 deficiency over MKK6 dysregulation. Indeed, we observed that the deletion of SIRT1 in ECFC increased MKK6 expression and that MKK6 expression negatively correlated with SIRT1 level. Previous studies demonstrated that SIRT1 is associated with specific changes in chromatin during aging and represses several genes by regulating the chromatin structure through its histone deacetylase activity^55, 59, 60^. For the first time, our data identify MKK6 (MAP2K6) as a direct target of SIRT1, which bound to its promoter and represses it at chromatin level. Of note, we demonstrated that H3K9Ac enrichment, a hallmark of transcription activation, at the MKK6 enhancer plays an important role in senescence-induced MKK6 upregulation in PT-ECFC and is regulated by the deacetylase activity of SIRT1. Thus, SIRT1-mediated increase in open chromatin at the MKK6 promoter may be crucial for its activity. In line with this, previous reports indicated that accumulation of active chromatin markers such as H3K9Ac was associated with SIRT1 depletion and increased expression of pro-aging and inflammatory genes^35, 61^. Thus, repression of secretory profiles (consisting of soluble factors and MP) may contribute to the anti-aging effect of SIRT1, leading to protection against the endothelial dysfunction of age-associated vascular diseases. However, future studies are needed to identify the mechanism that targets SIRT1 to MAP2K6 locus in ECFC. Such mechanism is likely to play an important role in repressing MKK6 expression and senescence-associated phenotype.

Finally, this work and our previous data that highlight the importance of SIRT1 in endothelial homeostasis may expose new reversion strategies with global effects on senescence and its consequences in the prematurity model. Among them, various environmental, lifestyle and pharmacologic interventions, such as caloric restriction, exercise, and resveratrol, are now well-appreciated for their capacity to modulate SIRT1 and related post-translational modifications^62, 63^. Correcting the function of SIRT1, a critical modulator of the signaling pathways controlling aging-related processes and the balance of histone acetylation, may thus emerge as a promising therapeutic target^53^. In addition, the SASP is becoming an obvious pharmaceutical target to correct senescence-associated effects. Therefore implementation of “senolytic” therapy, recently reported^64^, has been considered to reduce the SASP contribution. Another perspective could be to limit the biogenesis of EMP as part of the SASP. Our study not only provides news insights into the molecular mechanisms of MP formation in the context of SIPS and their potential role in preterm birth-associated vascular risk, but also opens avenues regarding new targeted therapeutic strategies for maintaining vascular homeostasis^56, 65^.

In conclusion, this study demonstrates that SIRT1 deficiency acts as a major determinant of EMP biogenesis associated with SIPS of endothelial cells through a novel mechanism involving the upregulation of MKK6 gene expression. Targeting the molecular pathways that link SIPS, gene expression changes and SASP components may offer new perspectives to control the progression of vascular alterations in individuals born preterm and, more generally, to limit endothelial pathogenesis in accelerated aging-related vascular diseases.

## Methods

Detailed methods are provided in the online data supplement.

### Patients

Eighteen term (control, gestational age (GA) >37 weeks, appropriate weight) and twenty-nine preterm neonates (GA 24 to 35 weeks with appropriate or small weight for GA), were included. Exclusion criteria were congenital viral infections, major congenital heart or structural brain malformations, genetic abnormalities and metabolic diseases. This research was approved by a local ethic committee Assistance Publique Hôpitaux de Marseille and the study was performed conform the declaration of Helsinki. All the parents have provided written informed consent for the use of cord blood. The patient characteristics are shown in Table S1.

### Isolation of endothelial colony-forming cells

ECFC were isolated and expanded from mononuclear cell fraction (MNC) obtained from the cord blood of term (CT) and preterm (PT) neonates, cultured and characterized as previously described^12^. ECFC were used between the third and fourth passage.

### Antibody arrays

Conditioned media for antibody array analysis were prepared by washing cells with PBS and incubating them in basal medium for 48 h. The conditioned media were collected in a centrifuge tube, and the cells remaining on the dish were counted to normalize conditioned media volumes by cell number. The conditioned media were clarified by brief centrifugation, 0.2 µm filtered, diluted with a serum-free medium to a concentration equivalent to 1.35 × 10^5^ cells per 1.3 ml, and applied to the antibody arrays (Raybiotech; AAH-CYT-1) as described previously by Freund *et al*.^19^ and as recommended by the supplier. The signals were detected using a G-BOX Imaging System (GeneSys) and were analyzed using specific software (GeneTools, Syngene). Signals were averaged and displayed as described in the figure legend.

### ELISA

Conditioned media were prepared by incubating cells for 48 h as described above. The CM were analyzed using the Human IL-6 ELISA Kit II and reagents, following the procedures described by the manufacturer (BD OptEIATM; 550799).

### Counting and measuring particles

#### Tunable resistive pulse sensing

The concentration and size distribution of particles was analyzed with TRPS (qNano, Izon Science Ltd, Christchurch, New Zealand), a relatively new technology that allows the detection of particles passing through a nanopore by way of single-electrophoresis^66^. Calibration was performed using CPC200 OR SKP400 calibration particles (Izon) as a standard according to the manufacturer’s instructions. Data were recorded and analyzed using the Izon Control Suite software version 3.2.2.234.

#### Flow cytometric analysis of EMP

EMP were analyzed using a Gallios flow cytometer, as previously described by Robert *et al*.^67^. Briefly, conditioned medium was collected after 48 hours of ECFC culture (passage 3 to 4) in 0.22 µm filtered complete medium. After two initial centrifugation to discard debris (300 × g, 5 minutes) and apoptotic bodies (2000 × g, 15 minutes), EMP in resulting supernatant were quantified withth Annexin A5-FITC (AnnV-FITC, Tau Technology BV, Netherlands). CytoCount beads (Cyto-Count, Dako, Copenhagen, Denmarrk) were added as internal standard to samples before FC analysis to determine the concentration of EMP. The samples analyses were performed with the Kaluza® Analysis software (Beckman Coulter), as already described^68, 69^.

### Functional Analysis

#### Preparation of Conditioned Medium

Conditioned medium (CM) was collected after 48 hours of incubation with PT or CT-ECFC in completed medium and then clarified by two serial centrifugation steps (300 g for 5 min and 2000 g for 15 min, at 4 °C) to remove cells debris and apoptotic bodies. CM was stored at −80 °C for subsequent analyses. Fresh CM was used for EMP isolation and EMP-free CM preparation.

#### Microparticles Isolation

To obtain EMP fractions, clarified CM was subjected to differential ultracentrifugation steps (70,000 g; 90 min, from 4 to 8 °C). EMP-free CM was obtained after the first ultracentrifugation, 0.2 µm filtered to remove vesicles > 200 nm, and stored at −80 °C for subsequent analysis. The resultant EMP pellet was washed twice in PBS in the same conditions. The final pellet containing the EMP fractions were diluted in EBM2 and stored at −80 °C until subsequent use. The number of resulting EMP was quantified using high-sensitive flow cytometry as described above. The high-speed last wash supernatant was used as control (vehicle).

#### Cell culture

Human umbilical venous endothelial cells (HUVEC) were isolated from term neonates cord vein according to the method of Jaffe *et al*.^70^ and used at the fourth passage. HUVEC were cultured in EGM2 media (Lonza) and cells were stimulated with CM ± depleted in EMP or in complete medium with vehicle (SN), CT-EMP or PT-EMP (50 EMP/Cell).

#### Senescence-Associated-ß-galactosidase (SA-ß-gal) staining

SA-ß-Gal activity was performed using a Promokine Senescence detection kit (PK-CA577-K320, PromoCell) according to the manufacturer’s instructions. Percentage of SA ß-gal positive cells was counted in 10 randomly selected microscopic fields (magnification ×20; 400–600 cells).

#### Proliferation assay and cell cycle analysis

Proliferative capacity was analyzed using a bromodeoxyuridine (BrdU) incorporation assay (Roche Molecular Biochemicals) according to the manufacturer’s instructions. Cell cycle analysis was conducted by using a propidium iodide-based flow cytometry protocol. HUVEC were incubated for 24 hours with CM or EMP from each condition, harvested, fixed with 70% cold ethanol, and stained with propidium iodide. At least 10,000 events were acquired per sample with a Gallios flow cytometer system and analyzed using Kaluza® Analysis software (Beckman Coulter).

#### THP-1 adhesion to HUVEC

THP-1 monocytic cells were cultured in RPMI 1640 with 10% heat-inactivated FCS. Adhesion of the monocytic cell line THP-1 to HUVEC was performed as described by Akeson *et al*.^71^ using calcein-labeled cells. HUVEC were stimulated with CM or EMP from each condition, for 48 h. At the end of the stimulation, calcein-labeled THP-1 were incubated with HUVECs for 30 min in RPMI medium. Adhesion to HUVEC was measured according to the method published by Vaporcyan *et al*.^72^. Experiments were performed in triplicates.

### Transfection

PT-ECFC were transfected with the pCMV-Sport6 expression vector (Empty) or the pCMV-Sport6-SIRT1 plasmid (NIH_MGC_91 clone) using the jetPEI^TM^-HUVEC *in vitro* DNA transfection protocol (Polyplustransfection SA, Illkirch, France). CT-ECFC were transfected with the SignalSilence® SIRT1 siRNA (#12241, Cell Signaling) or SignalSilence® Control siRNA using the jetPRIME *in vitro* siRNA transfection protocol (Polyplustransfection SA, Illkirch, France).

### Drug Treatment

ECFC were incubated with 1μM Resveratrol (RSV) (Calbiochem, La Jolla, USA), 2 µM SB203580 (p38MAP Kinase inhibitor) suspended in DMSO. For nicotinamide (NAM), medium containing 1 mM NAM was added to cells. All the doses for drug treatment were determined by dose and time-response experiments in pilot experiments or previous studies^12^. Control cells were mock-treated with DMSO.

### Western Blot

After transfections and/or treatment with chemicals or not, cells were lysed for the Western Blot assay. Bands were incubated with primary antibodies against SIRT1 (#2493),p16^**INK4a**^ (#4824), p21^*WAF*^ (#2947), p53 (#9282), MKK6 (#8550),MKK3 (#5674), Phospho-MKK3/MKK6 (#9236), Phospho-p38 MAP Kinase (#9211), p38 MAP Kinase (#9212), Phospho-Hsp27 (#2401), Phospho-MAPKAPK2 (#3007), Phospho-ATF2 (#5112) and actin (#8457) purchased from Cell Signaling Technology (Danvers, MA) and used at the recommended dilution for immunoblotting (1:1000). Raw data presenting full-length blots bands with molecular size markers are shown in Figure S9.

### RNA isolation and quantitative RT-PCR

Total RNA was extracted using the mirVana miRNA Isolation Kit (Ambion), according to the manufacturer’s recommendations. Two-step RT-PCR was performed. Total RNA was reverse transcribed into cDNA using the High Capacity cDNA Archive Kit (Applied Biosystems, Foster City, California, USA). cDNA product was amplified in a 20 µl reaction on MxP3000 (Stratagene, NL) using the Brillant QPCR Master Mix (Stratagene, La Jolla, CA) using pre-designed primers for SIRT1 (HS01009003_m1), p16^INK4a^ (HS00233365_m1), p21^*WAF*^(HS00355782_m1), p53 (HS01034249_m1), MAP2K6 (HS00992389_m1) and RPL13A (HS00204173_m1) (Applied Biosystems). Each sample was run in duplicate, and the relative fold change was determined using the 2^−ΔΔCT^ methods with CT-ECFC as baseline, normalized to RPL13A expression.

### Microarrays

The microarray study was performed using microarrays chip that included 45,000 probes (1 microarray for each sample, 4 × 44 K Whole Genome Microarray G4112F) and the One-Color Microarray-Based Gene Expression Analysis based on the Agilent Technologies procedures^73^. cRNA labeling and hybridization performance were performed and all parameters checked were found within the manufacturers specifications. Signal intensities on 20 bit tiff images were calculated by Feature Extraction software (FE, Version 8.5; Agilent Technologies). Data analyses were conducted with GeneSpring GX software (Vers.13.1.1; Agilent Technologies). We considered genes to be differentially expressed when the adjusted p value was below 0.05, as determined by a moderated T-test supplemented with Benjamini-Hochberg multiple testing corrections, and the absolute fold-change (FC) was higher than 1.5. Unsupervised analyses were performed using principal component analysis (PCA) and hierarchical clustering. The average linkage was based on the Pearson correlation distance. Microarray data are available in the ArrayExpress database (www.ebi.ac.uk/arrayexpress) under accession number E-MTAB-4860.

### Chromatin Immunoprecipitation (ChIP) and real-time PCR

Chromatin immunoprecipitation was performed as described previously^74^. Precleared chromatin was incubated overnight at 4°C with 5 μl of primary antibodies specific for SIRT1 (#07-131) acH3K9 (#07-352) and H3 (#04-928) purchased from Millipore. After collection of immune complexes, DNA was recovered and quantified. Analysis of ChIP DNA samples was performed by quantitative PCR with the specific promoter regions described in the Supplemental Table VII. Real-time PCR experiments were performed using the SYBR® Premix Ex Taq^TM^ (Takara Bio Inc, Japan) and analyzed using the stepOnePlus™ Real-Time PCR System (Applied Biosystems). qPCR values were normalized to the values obtained with the positive control (Chromosome 5) and to input DNA. For histone mark ChIP, after Chromosome 5 normalization, data were further normalized for the total histone H3 signal using the (2^Ct(IP)-Ct(Ref)^) equation.

### Statistical Analysis

All the statistical analyses were performed using the software GraphPad Prism software and significance was calculated with 95% confidence interval (alpha < 0.05) Data are expressed as means ± SEM. Demographic data of the preterm and term populations were analyzed qualitatively using the chi2 test of Pearson and quantitatively using the 2-tailed unpaired t test. Normality was confirmed with a D’Agostino and Pearson omnibus test. For data normally distibuted, statistical significance was assessed by an unpaired or pair Student t-test, as appropriate. For data not normally distributed, we used a Mann-Whitney or Wilcoxon test, as appropriate. When ANOVA was utilized, post-hoc intergroup comparison were analyzed for statistical significant differences using either Tukey’s (all group compared to each other) or Dunnet’s (groups compared to control group) methodology, as appropriate. Statistical significance was accepted at p-value < 0.05.

## Electronic supplementary material

Supplemental data
