# Supplementary material for: Biogenesis of Pro-senescent Microparticles by Endothelial Colony Forming Cells from Premature Neonates is driven by SIRT1-Dependent Epigenetic Regulation of MKK6
Source: Sci Rep. 2017 Aug 15;7:8277. doi: 10.1038/s41598-017-08883-1 (PMC5557933; doi:10.1038/s41598-017-08883-1)
Supplement: Supplementary file 1 — Supplemental data [file 41598_2017_8883_MOESM1_ESM.pdf]

SUPPLEMENTAL MATERIAL

Biogenesis of Pro-senescent Microparticles by Endothelial Colony Forming Cells from Premature Neonates is driven by SIRT1-Dependent Epigenetic Regulation of MKK6

Stéphanie Simoncini[1,§], Anne-Line Chateau[1,6,§], Stéphane Robert[1], Dilyana Todorova[1], Catherine Yzydorzick[2], Romaric Lacroix[1], Isabelle Ligi[3], Laurence Louis[4], Richard Bachelier[1], Umberto Simeoni[2], Frédérique Magdinier[4], Françoise Dignat-George[1,5] and Florence Sabatier[1,6]

[1]Aix Marseille Univ, INSERM, VRCM, Marseille, France; [2]Service de pédiatrie, Université de Lausanne, CHUV, 1011, Lausanne, Suisse; [3]APHM, CHU de la Conception, Département de Néonatologie, Marseille, France; [4]Aix Marseille Univ, INSERM, GMGF, Marseille, France; [5]APHM, CHU de la Conception, Service d'hématologie, Marseille, France; [6]APHM, CHU de la Conception, Laboratoire de culture et thérapie cellulaire, INSERM, CBT-1409, Marseille, France. [§]The authors contributed equally to this work.

Address correspondence to:
Pr. F. Dignat-George
VRCM, INSERM UMR_S 1076,
Faculté de Pharmacie
27 Bd Jean Moulin 13385
Marseille cedex 05 France.
Tel: 33 4 918 356 86
Fax: 33 4 918 356 02
Email: francoise.dignat-george@univ-amu.fr

Expanded Materials and Methods

Patients

Eighteen term (control, gestational age (GA) > 37 weeks, appropriate weight) and twenty-nine preterm neonates (GA 24 to 35 weeks with appropriate or small weight for GA), were included. Exclusion criteria were congenital viral infections, major congenital heart or structural brain malformations, genetic abnormalities and metabolic diseases. This research was approved by a local ethic committee Assistance Publique Hôpitaux de Marseille and the study was performed conform the declaration of Helsinki. All the parents have provided written informed consent for the use of cord blood. The patient characteristics are shown in **Table S1**.

Isolation of endothelial colony-forming cells.

ECFC were isolated and expanded from mononuclear cell fraction (MNC) obtained from the cord blood of term (CT) and preterm (PT) neonates, cultured and characterized as previously described[1]. ECFC were used between the third and fourth passage.

Antibody arrays

Conditioned media for antibody array analysis were prepared by washing cells with PBS and incubating them in basal medium for 48h. The conditioned media were collected in a centrifuge tube, and the cells remaining on the dish were counted to normalize conditioned media volumes by cell number. The conditioned media were clarified by brief centrifugation, 0.2µm filtered, diluted with a serum-free medium to a concentration equivalent to 1.35 x 10^5 cells per 1.3ml, and applied to the antibody arrays (Raybiotech; AAH-CYT-1) as described previously by Freund et al[2] and as recommended by the supplier. The signals were detected using a G-BOX Imaging System (GeneSys) and were analyzed using specific software (GeneTools, Syngene). Signals were averaged and displayed as described in the figure legend.

ELISA

Conditioned media were prepared by incubating cells for 48h as described above. The CM were analyzed using the Human IL-6 ELISA Kit II and reagents, following the procedures described by the manufacturer (BD OptEIATM; 550799).

Counting and measuring particles.

i. **Tunable resistive pulse sensing.** The concentration and size distribution of particles was analyzed with TRPS (qNano, Izon Science Ltd, Christchurch, New Zealand), a relatively new technology that allows the detection of particles passing through a nanopore by way of single-electrophoresis[3]. The technology is based on the Coulter principle at the nano scale and operates by detecting transient changes in the ionic current generated by the transport of the target particles through a size-tunable nanopore in a polyurethane membrane. Samples were diluted to half in PBS buffer in a small sterile tube and analyzed using both an NP150 (size range 50 to 250nm) and NP400 (size range 200 to 1000nm) nanopore at a 45mm stretch. Calibration was performed using CPC200 OR SKP400 calibration particles (Izon) as a standard

according to the manufacturer's instructions. Data were recorded and analyzed using the Izon Control Suite software version 3.2.2.234

ii. **Flow cytometric analysis of EMP**. EMP were analyzed using a Gallios flow cytometer, as previously described by Robert et al[4]. Briefly, conditioned medium was collected after 48 hours of ECFC culture (passage 3 to 4) in 0.22µm filtered complete medium. After two initial centrifugation to discard debris (300 x g, 5 minutes) and apoptotic bodies (2000 x g, 15 minutes), EMP in resulting supernatant were quantified. The EMP labeling was based on a 30min incubation of 30µl of supernatant with Annexin A5-FITC (AnnV-FITC, Tau Technology BV, Netherlands). Thereafter, 500µl of calcium buffer was added to improve the binding of Annexin A5 to phosphatidylserine. Then, 30µl of CytoCount beads (Cyto-Count, Dako, Copenhagen, Denmarrk) were added as internal standard to samples before FC analysis to determine the concentration of EMP. EMP concentrations (EMP/µl) were calculated by the following formula: (Number of events AnnexinV x Total amount of flow count beads)/ Number of acquired beads. The samples analyses were performed with the Kaluza® Analysis software (Beckman Coulter), as already described[5,6].

Transfection.

PT-ECFC were transfected with the pCMV-Sport6 expression vector (Empty) or the pCMV-Sport6-SIRT1 plasmid (NIH_MGC_91 clone) using the jetPEI™-HUVEC *in vitro* DNA transfection protocol (Polyplustransfection SA, Illkirch, France). CT-ECFC were transfected with the SignalSilence® SIRT1 siRNA (#12241, Cell Signaling) or SignalSilence® Control siRNA using the jetPRIME in vitro siRNA transfection protocol (Polyplustransfection SA, Illkirch, France).

Drug Treatment.

ECFC were incubated with 1µM Resveratrol (RSV) (Calbiochem, La Jolla, USA), 2µM SB203580 (p38MAP Kinase inhibitor) suspended in DMSO. For nicotinamide (NAM), medium containing 1mM NAM was added to cells. All the doses for drug treatment were determined by dose and time-response experiments in pilot experiments or previous studies.[1] Control cells were mock-treated with DMSO.

Senescence-Associated-ß-galactosidase (SA-ß-gal) staining.

SA-ß-Gal activity was performed using a Promokine Senescence detection kit (PK-CA577-K320, PromoCell) according to the manufacturer's instructions. Percentage of SA ß-gal positive cells was counted in 10 randomly selected microscopic fields (magnification x20; 400-600 cells).

Apoptosis.

The number of apoptotic ECFC was determined by staining with the FITC-Annexin V/7-AAD kit according to the manufacturer's instructions (Beckman Coulter) on a Gallios Flow cytometer (Beckman Coulter).

Western Blot analysis.

For western blot analysis, equal amount of proteins (30µg) from ECFC were separated on 4-12% gradient SDS-polyacrylamide gel, blotted on cellulose C+ membranes. Equal loading

was verified using Ponceau red solution. Membranes were blocked in 3% BSA-TBS, 1 hours at RT before proceeding to the antibody incubation. All primary antibody incubation was performed in blocking buffer overnight at 4°C. Horseradish peroxidase-conjugated anti-mouse or anti-rabbit antibodies were used as secondary antibodies and incubated for 1h at RT. Immunocomplexes were visualized by chemiluminescence using ECL according to manufacturer's instuctions (Pierce, #32106). A G-BOX Imaging System (GeneSys) was used to catch up the specific bands, and the optical density of each band was measured using specific software (GeneTools, Syngene). After initial immunodetection, membranes were stripped of antibodies and re-probed with antibody against total protein or another protein with the same molecular weight. All proteins for each panel were assessed on one membrane, therefore actin expression need to be determined once to control for loading for all these proteins. The difference between the proteins of interest and the control loading protein of the same sample was calculated as relative content and presented graphically.

The antibodies against SIRT1 (#2493),p16$^{\mathbf{INK4a}}$ (#4824), p21^{WAF} (#2947), p53 (#9282), MKK6 (#8550),MKK3 (#5674), Phospho-MKK3/MKK6 (#9236), Phospho-p38 MAP Kinase (#9211), p38 MAP Kinase (#9212), Phospho-Hsp27 (#2401), Phospho-MAPKAPK2 (#3007), Phospho-ATF2 (#5112) and actin (#8457) were purchased from Cell Signaling Technology (Danvers, MA) and used at the recommended dilution for immunoblotting (1:1000).

RNA isolation and quality control.

ECFC were harvested from cultures dishes and total RNA was extracted using the mirVana miRNA Isolation Kit (Ambion), according to the manufacturer's recommendations. The quantity and quality of the RNA were assessed using a Nanodrop (Thermo Science, Orsay, France) and a 2100 bioanalyzer (Agilent Technologies, Massy,France), respectively. All samples used in microarrays study, showed common, high quality RNA Integrity Numbers (RIN 9.0-9.8).

Quantitative-Real-Time PCR.

Two-step RT-PCR was performed. Total RNA was reverse transcribed into cDNA using the High Capacity cDNA Archive Kit (Applied Biosystems, Foster City, California, USA). cDNA product was amplified in a 20µl reaction on MxP3000 (Stratagene, NL) using the Brillant QPCR Master Mix (Stratagene, La Jolla, CA) using pre-designed primers for SIRT1 (HS01009003_m1), p16^{INK4a} (HS00233365_m1), p21^{WAF}(HS00355782_m1), p53 (HS01034249_m1), MAP2K6 (HS00992389_m1) and RPL13A (HS00204173_m1) (Applied Biosystems). The PCR program consisted of an initial denaturation at 95°C for 10 minutes followed by amplification for 40 cycles (95°C for 15 seconds, 60°C for 1minute). Each sample was run in duplicate, and the relative fold change was determined using the $2^{-\Delta\Delta CT}$ methods with CT-ECFC as baseline, normalized to RPL13A expression.

Microarrays.

The microarray study was performed using microarrays chip that included 45,000 probes (1 microarray for each sample, 4x44K Whole Genome Microarray G4112F) and the One-Color Microarray-Based Gene Expression Analysis based on the Agilent Technologies procedures[7]. Briefly, 400ng of total RNA were converted to cDNA, followed by in vitro transcription and incorporation of Cy3-CTP into nascent cRNA. The hybridization was performed for 17h at 65°C. cRNA labeling and hybridization performance were performed and all parameters checked were found within the manufacturers specifications. Arrays were

scanned as described in the manufacturers' protocol. Signal intensities on 20 bit tiff images were calculated by Feature Extraction software (FE, Version 8.5; Agilent Technologies).

Data analyses were conducted with GeneSpring GX software (Vers.13.1.1; Agilent Technologies). Probe signal intensities were quantile normalized across all samples to reduce inter-array variability[8]. Input data pre-processing was established by baseline transformation to the median of all samples. After grouping of biological replicates according to their respective experimental condition, a given transcript had to be expressed above background (i.e. called "detected' or "Marginal" by GeneSpring) in at least 80.0 percent of samples in any 1 out of 2 conditions to be further analyzed in pairwise comparisons of conditions.

We considered genes to be differentially expressed when the adjusted p value was below 0.05, as determined by a moderated T-test supplemented with Benjamini-Hochberg multiple testing corrections, and the absolute fold-change (FC) was higher than 1.5. Unsupervised analyses were performed using principal component analysis (PCA) and hierarchical clustering. The average linkage was based on the Pearson correlation distance. Microarray data are available in the ArrayExpress database (www.ebi.ac.uk/arrayexpress) under accession number E-MTAB-4860. Functional enrichment analysis was performed on selected genes with the DAVID bioinformatics tool using Gene Ontology (GO) pathways. Keywords were selected when the Benjamini-Hochberg-Corrected p value for enrichment was less than 0.01.

Pathway analysis was carried out using Pathway Analysis Module of GeneSpring. The Biopax pathways/networks exchange format from public databases (Wikipathways and KEGG, available on line: http://www.wikipathways.org and http://www.genome.jp/kegg) was imported into GeneSpring and the Find Significant Pathway tool was then used to identify the biological pathway for which there was significant enrichment in the differential expression gene list. Pathways with at least 5 differential expressed genes and a p-value≤0.001 were considered for analysis.

Chromatin Immunoprecipitation (ChIP) and real-time PCR.

Chromatin immunoprecipitation was performed as described previously[9]. Precleared chromatin was incubated overnight at 4°C with 5 µl of primary antibodies specific for SIRT1 (#07-131) acH3K9 (#07-352) and H3 (#04-928) purchased from Millipore. After collection of immune complexes, DNA was recovered and quantified. Analysis of ChIP DNA samples was performed by quantitative PCR with the specific promoter regions described in the **Supplemental Table VII**. Real-time PCR experiments were performed using the SYBR® Premix Ex Taq™ (Takara Bio Inc, Japan) and analyzed using the stepOnePlus™ Real-Time PCR System (Applied Biosystems). qPCR values were normalized to the values obtained with the positive control (Chromosome 5) and to input DNA. For histone mark ChIP, after Chromosome 5 normalization, data were further normalized for the total histone H3 signal using the ($2^{Ct(IP)-Ct(Ref)}$) equation.

Functional Analysis

Preparation of Conditioned Medium

Conditioned medium (CM) was collected after 48 hours of incubation with PT or CT-ECFC in completed medium and then clarified by two serial centrifugation steps (300g for 5min and

2000g for 15min, at 4°C) to remove cells debris and apoptotic bodies. CM was stored at -80°C for subsequent analyses. Fresh CM was used for EMP isolation and EMP-free CM preparation.

Microparticles Isolation.

To obtain EMP fractions, clarified CM was subjected to differential ultracentrifugation steps (70,000g; 90min, from 4 to 8°C). EMP-free CM was obtained after the first ultracentrifugation, 0.2µm filtered to remove vesicles > 200nm, and stored at -80°C for subsequent analysis. The resultant EMP pellet was washed twice in PBS in the same conditions. The final pellet containing the EMP fractions were diluted in EBM2 and stored at -80°C until subsequent use. The number of resulting EMP was quantified using high-sensitive flow cytometry as described above. The high-speed last wash supernatant was used as control (vehicle).

Cell culture

Human umbilical venous endothelial cells (HUVEC) were isolated from term neonates cord vein according to the method of Jaffe et al[10] and used at the fourth passage. Early passage normal HUVEC were used as target cells to exclude the confounding effects of replicative senescence. HUVEC were cultured in EGM2 media (Lonza) and cells were stimulated with CM ± depleted in EMP or in complete medium with vehicle (SN), CT-EMP or PT-EMP (50 EMP/Cell). The doses of EMP were chosen from pilot experiments aimed at identifying the optimal induction of senescence.

Senescence-Associated-ß-galactosidase (SA-ß-gal) staining.

CM or EMP from each condition were added to naïve cells (6,000 cells/wells) in 96-well culture dishes and incubated for 48h. SA-ß-Gal activity was performed using a Promokine Senescence detection kit as described above.

Proliferation assay

The effect of CM or EMP treatment on HUVEC proliferation was assessed by measurement of BrdU incorporation using the Cell proliferation assay Kit according to the manufacturer's instructions (Roche Diagnostic Mannhein Germany). HUVEC were plated at 6,000 cells/wells in gelatin-coated 96-well culture plates in triplicate and were allowed to attach in complete medium. Subsequently, CM ± depleted in EMP or medium containing either vehicle (SN) or CT or PT-EMP was added for 24h. Thereafter, 10µM BrdU was added to each well and incubated overnight. BrdU incorporation was assayed by spectrophotometry using an optical density of 450nm.

Cell cycle analysis

Cell cycle analysis was conducted by using a propidium iodide-based flow cytometry protocol. HUVEC (35,000 cell/wells in 24-well culture dishes) were incubated for 24hours with CM or EMP from each condition, harvested, fixed with 70% cold ethanol, and stained with propidium iodide. At least 10,000 events were acquired per sample with a Gallios flow cytometer system and analyzed using Kaluza® Analysis software (Beckman Coulter).

THP-1 adhesion to HUVEC

THP-1 monocytic cells were cultured in RPMI 1640 with 10% heat-inactivated FCS. They were split one to tenth once a week. Adhesion of the monocytic cell line THP-1 to HUVEC was performed as described by Akeson et al [11] using calcein-labeled cells. HUVEC (6,000 cells per wells) were stimulated with CM or EMP from each condition, for 48h in 96-well plates. THP-1 cells were labeled with 10µM Calcein-AM (C3100MP, Invitrogen) at 37°C for 30min and 10^6cells/ml. At the end of the stimulation, 40,000 calcein-labeled THP-1 per well were incubated with HUVECs for 30min in RPMI medium. Adhesion to HUVEC was measured according to the method published by Vaporcyan et al[12]. Experiments were performed in triplicates.

Western Blot Analysis

Western blotting was used to examine levels of cell cycle/senescence proteins in HUVEC treated with medium containing vehicle (SN), CT or PT-EMP for 24hours. Cellular proteins were obtained, and western blotting was performed as described above. Antibodies of p16$^{\mathbf{INK4a}}$, p21^{WAF}, actin were purchased from Cell Signaling Technology Inc.

Statistical Analysis

All the statistical analyses were performed using the software GraphPad Prism software and significance was calculated with 95% confidence interval (alpha<0.05) Data are expressed as means ± SEM. Demographic data of the preterm and term populations were analyzed qualitatively using the chi2 test of Pearson and quantitatively using the 2-tailed unpaired t test. Normality was confirmed with a D'Agostino and Pearson omnibus test. For data normally distibuted, statistical significance was assessed by an unpaired or pair Student t-test, as appropriate. For data not normally distributed, we used a Mann-Whitney or Wilcoxon test, as appropriate. When ANOVA was utilized, post-hoc intergroup comparison were analyzed for statistical significant differences using either Tukey's (all group compared to each other) or Dunnet's (groups compared to control group) methodology, as appropriate. Statistical significance was accepted at p-value < 0.05.

Additional Figures and Tables

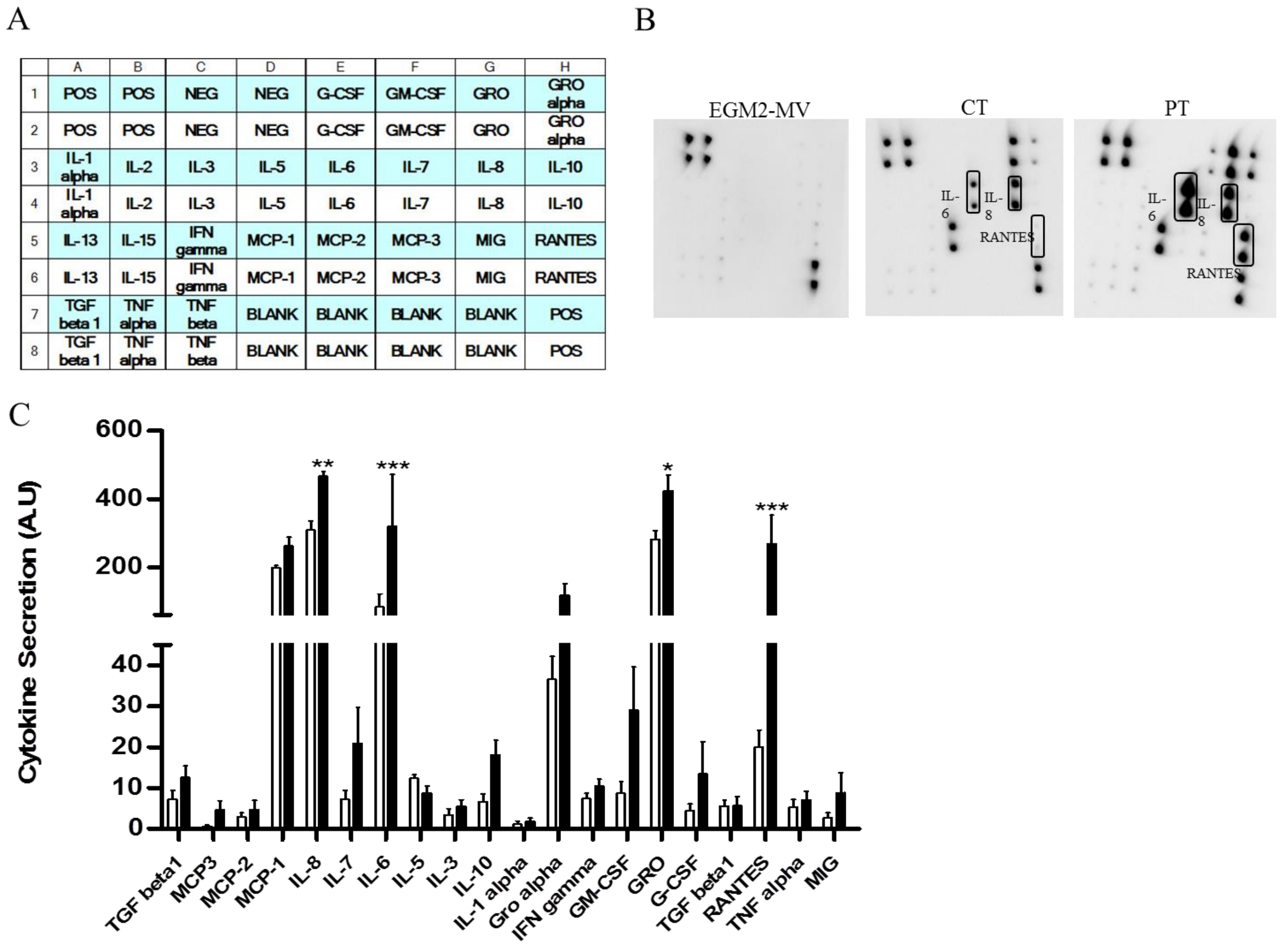

Figure S1. Prematutrity modulates cytokines secretory phenotype

(A) Cytokine array map from RayBiotech. **(B)** Representative images of cytokine array incubated with EGM2-MV only, conditioned media from CT and PT-ECFC obtained after 48h of culture. Boxes show the binding signals of IL6, IL8 and RANTES (**C**) Quantification of cytokines array signal of protein spot using GeneTools Syngen. Data are represented as means ± SEM from 4 CT- and 4 PT independent samples

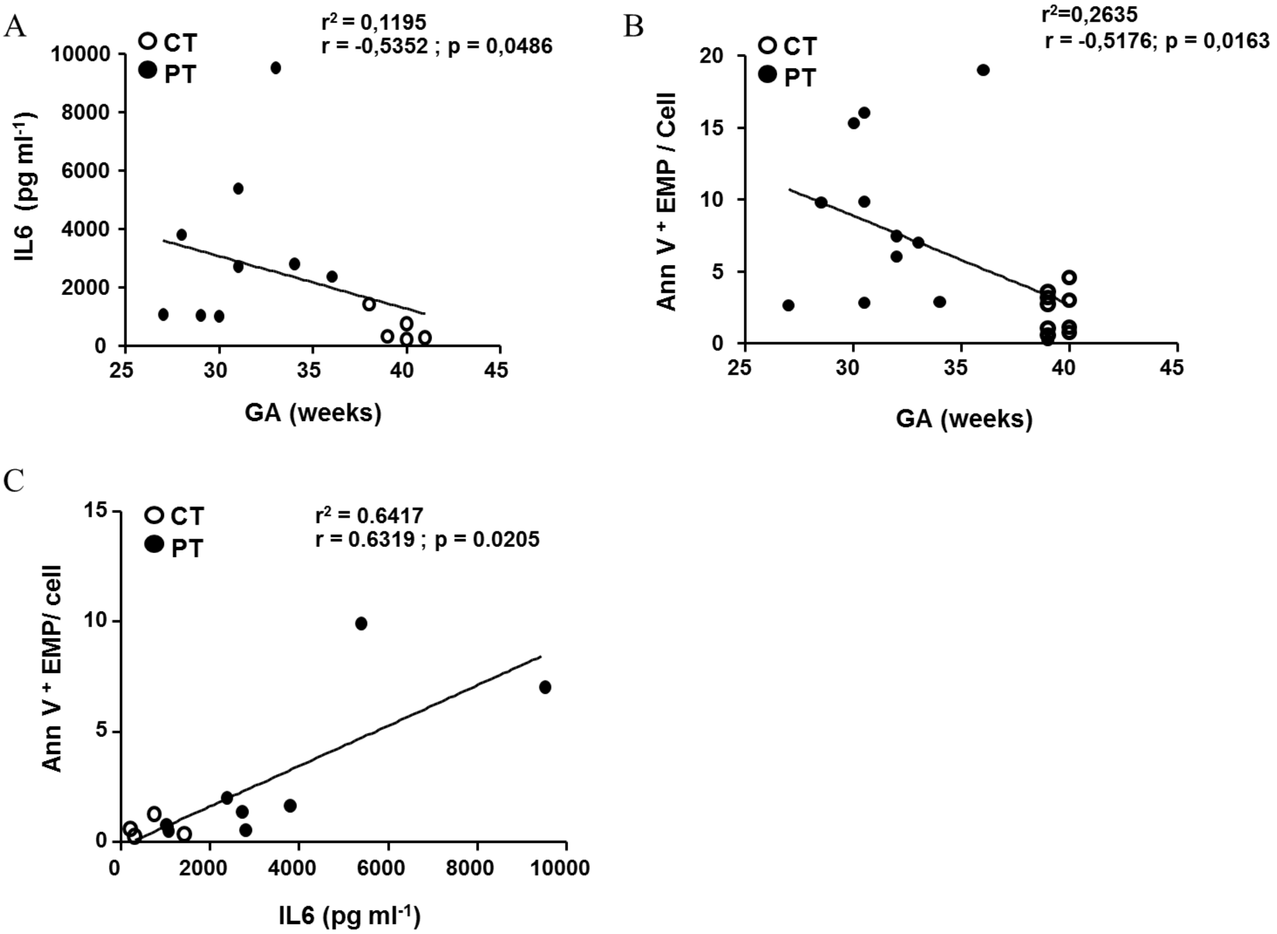

Figure S2. Relation between IL6, EMP levels and gestational aging (GA)

(A) IL6 level in conditioned medium negatively correlated with gestational age of neonates in samples from all groups (n=14);r, Spearman correlation coefficient. **(B)** $AnnV^+$ EMP level in conditioned medium negatively correlated with gestational age of neonates in samples from all groups (n=20); r, Pearson correlation coefficient. **(C)** $AnnV^+$ EMP correlated with IL6 levels in conditioned media in samples from all groups (n=13) r, Spearman correlation coefficient.

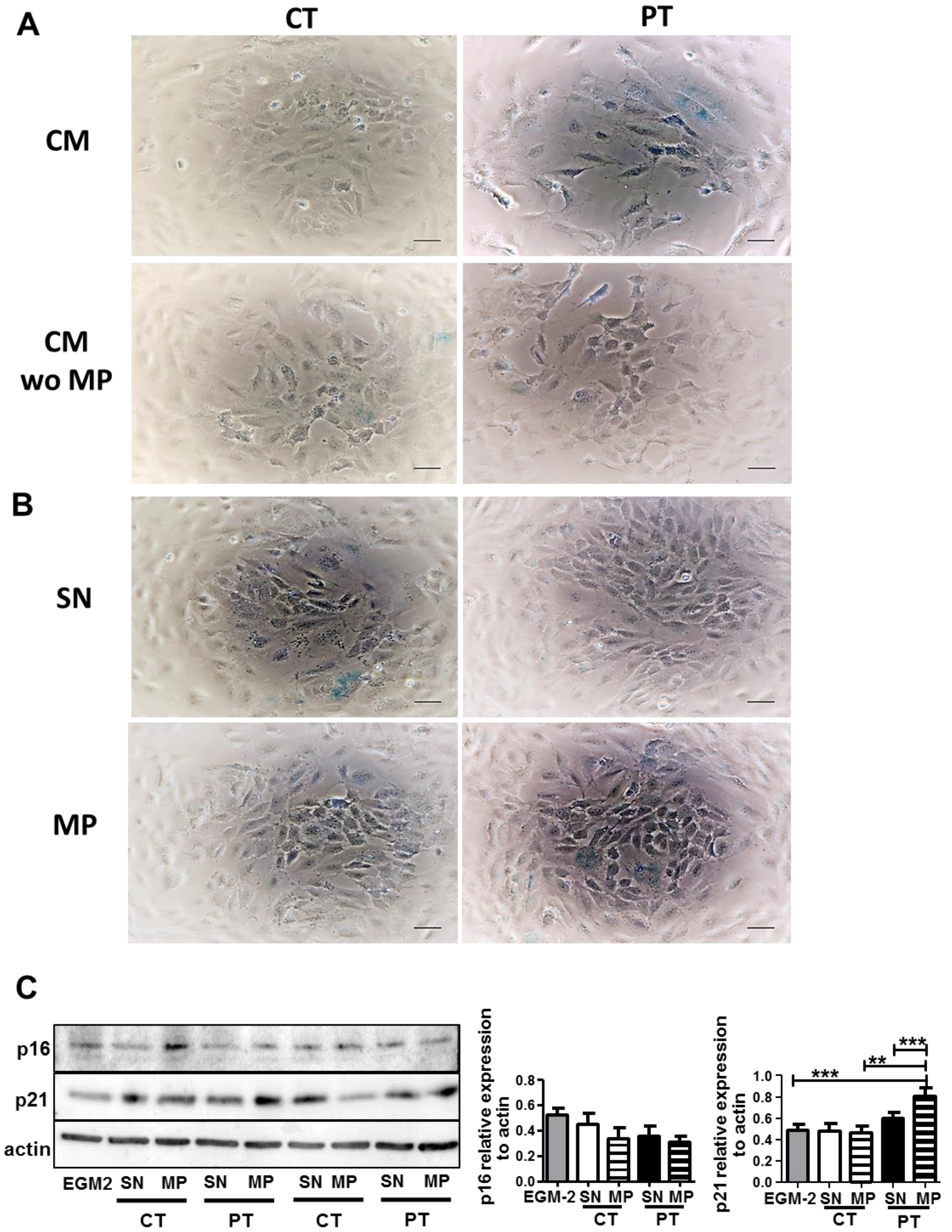

Figure S3. EMP components of PT-SASP mediate paracrine senescence

HUVEC were cultured and treated with conditioned media (CM) ± depleted in EMP **(A)** or purified EMP from CT- and PT-ECFC **(B)**. After 48h, SA-β-gal staining was performed for evaluation of the senescence status. Representative results of SA-β-galactosidase staining are shown (original magnification x20,scale bar, 49µm). **(C)** The impact of EMP treatment on p16 and p21 protein expression levels (relative expression to actin; HUVECs, N=7; N= 6 CT vs. 6 PT). Graphs represented means ± SEM. **: $p<0.01$, ***: $p<0.001$.

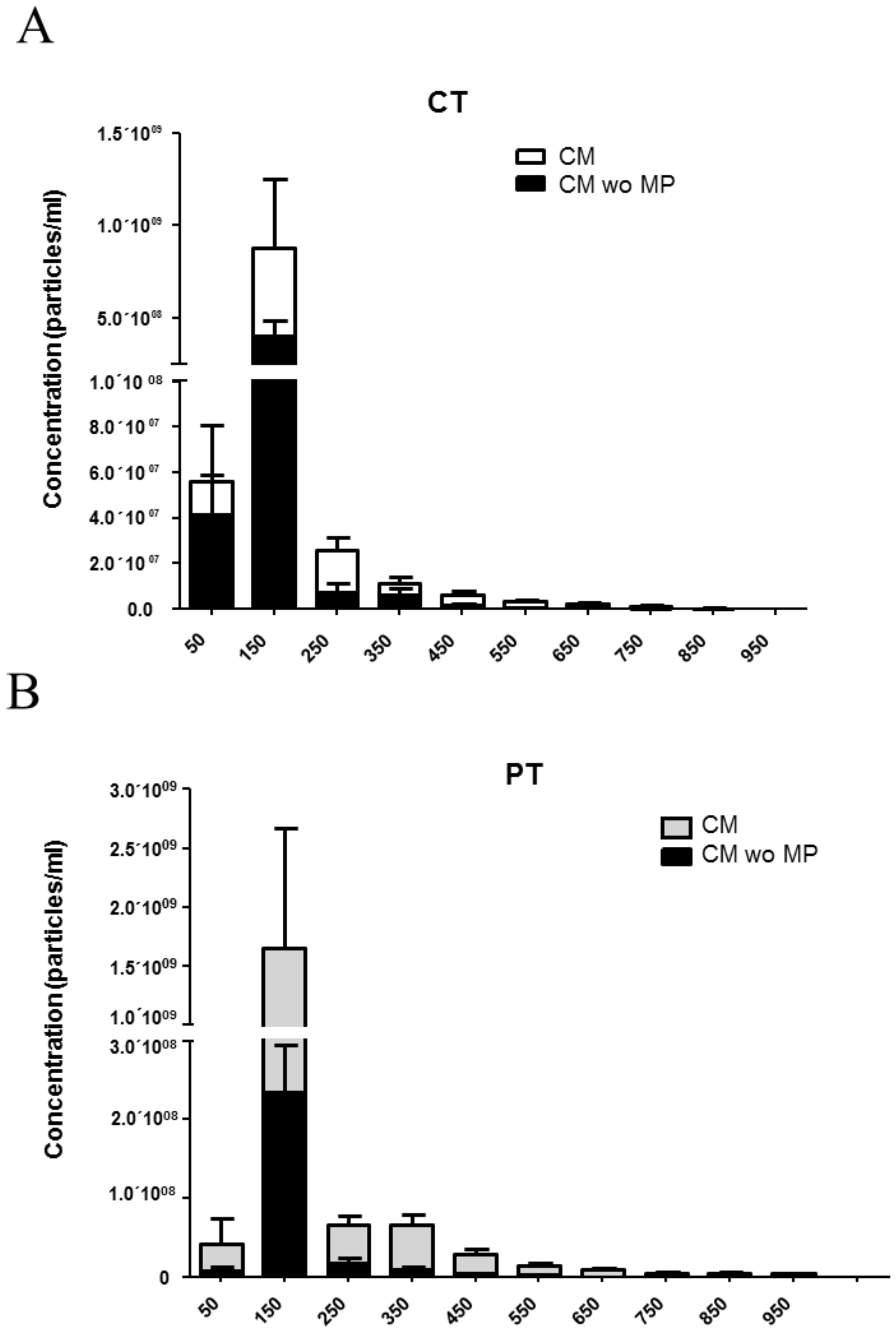

Figure S4. Sequential centrifugation and filtration of CM conducted to EMP depletion.

qNano analysis of conditioned media from **(A)** and PT-CM **(B)** ± depleted in EMP. The figure depicts the diameter of the vesicles (in nm) versus the normalized concentration of vesicles (in particles/ml). 100-nm bin size.

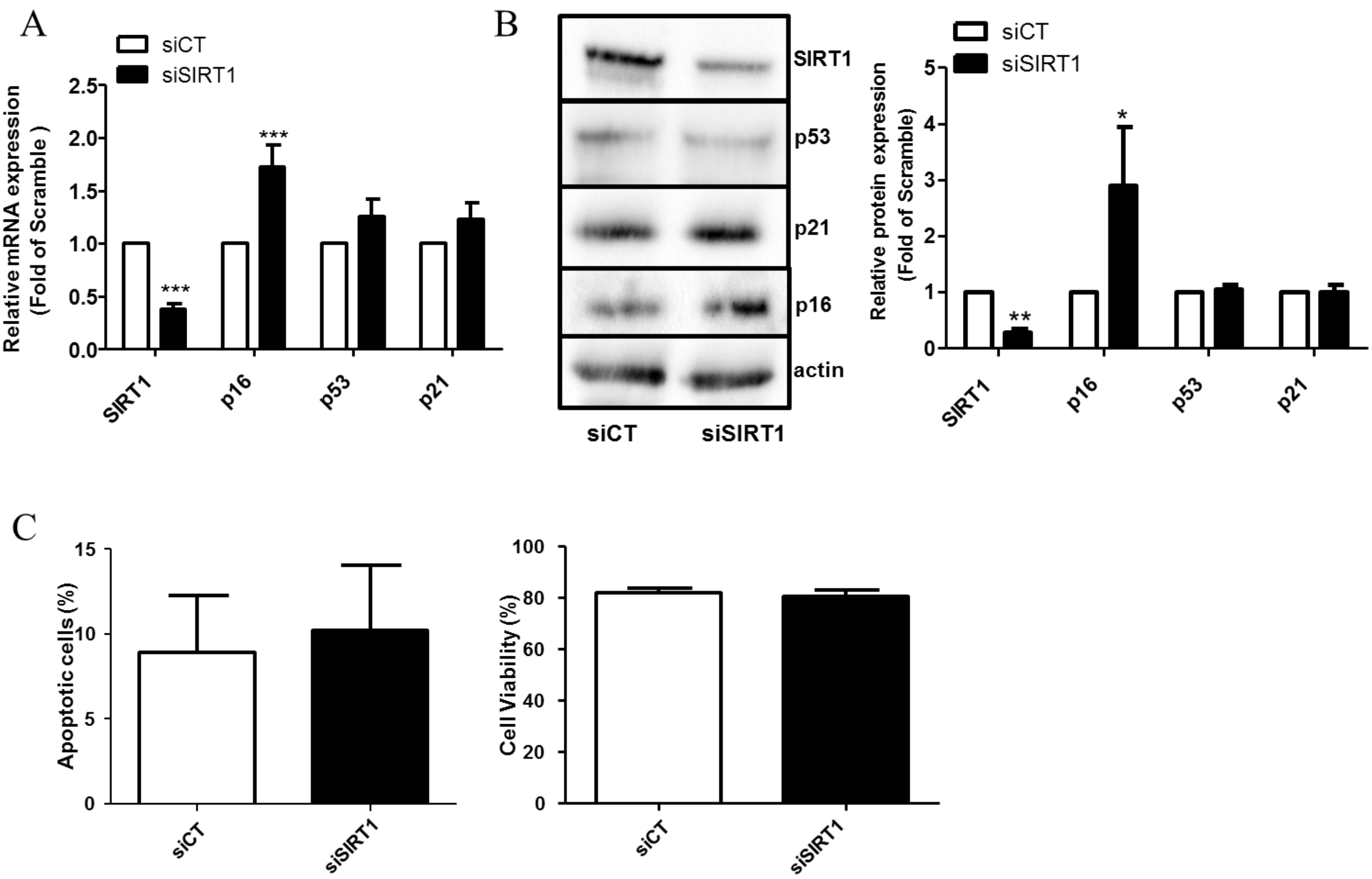

Figure S5. Modeling of PT-like senescence by SIRT1 silencing in CT-ECFCs

SIRT1 and senescent markers (p16, p53 and p21) mRNA **(A)** and protein **(B)** expression in CT-ECFC, transfected with SIRT1 siRNA (siSIRT1) or Scramble (siCT), were assessed by RT-qPCR and western blot analysis respectively. **(A)** Impact of SIRT1 silencing on mRNA level. Two days after transfection, SIRT1 knockdown was monitored by quantitative RT-PCR analysis on Stratagen MX3000, together with the expression of p 16, p53 and p21. Changes in mRNA were normalized to the housekeeping gene RPL13A. Results were expressed as fold induction of mRNA level in SIRT1 siRNA-transfected PT-ECFC compared with siCT. Graph represented means ± SEM of 11 independent experiments. *** $p<0.001$ **(B)** The impact of SIRT1 silencing on the senescence associated protein expression of CT-ECFCs was monitored by western blot. Representative experiment is shown. Actin was used as the internal control. The fold induction of protein expression in SIRT1-Knockdown CT-ECFC was calculated in comparison of matched scramble (siCT) transfected cells. Graphs represented means ± SEM of the relative intensity of 11 independent experiments.*$p<0.05$;**$p<0.01$ **(C)** Cell viability was analyzed by flow cytometry using fluorescent Annexin V membrane labeling and 7AAD incorporation into DNA. No difference in the number of viable, apoptotic or necrotic cells were observed between CT-ECFC transfected with siCT or siSIRT after 48h. Data are mean percentage of cells ± SEM of 6 independent experiments.

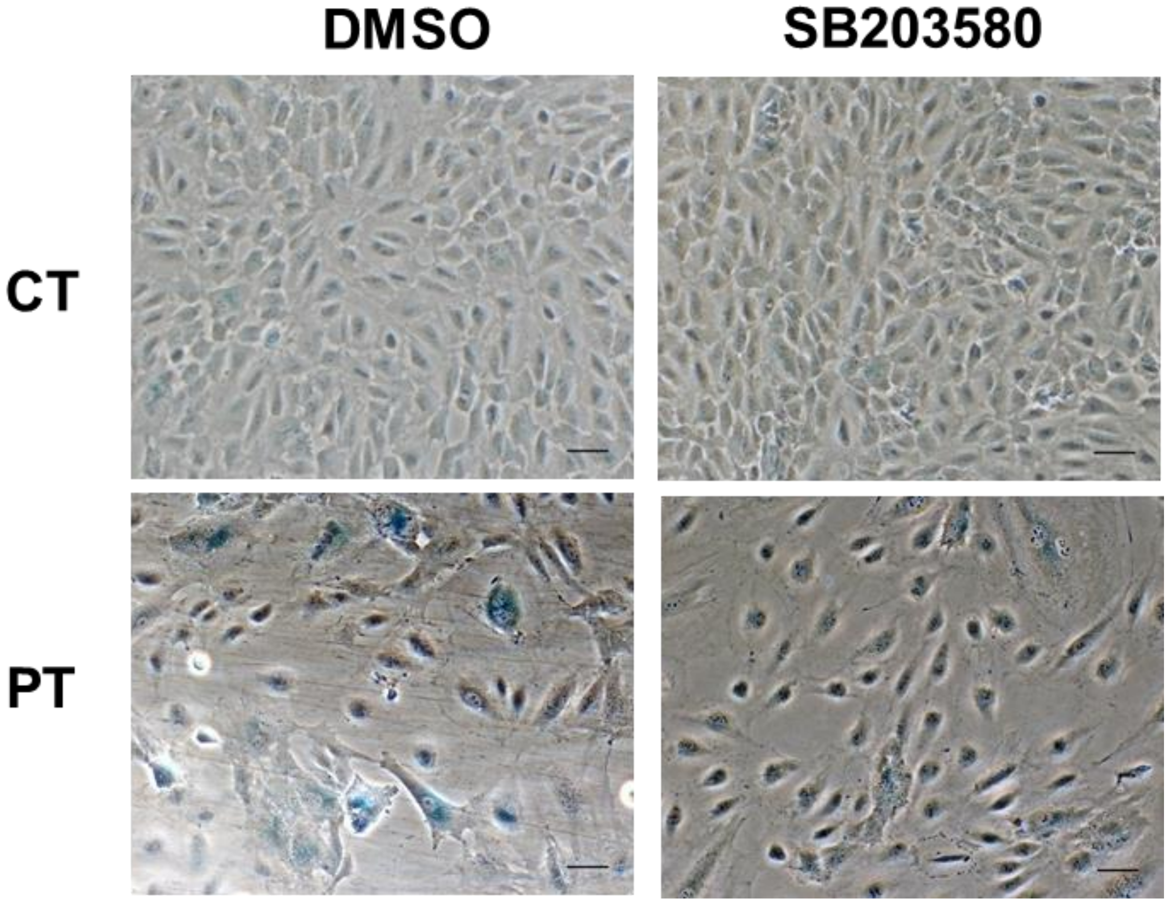

Figure S6. SB203580 treatment reverts PT-ECFC senescence

CT-ECFC and PT-ECFC were incubated in the presence or not of 2µM SB203580 for 48h. SA-β-gal staining was performed for evaluation of the senescence status. Representative images of SA-β-gal staining are shown (original magnification x20,scale bar, 49µm).

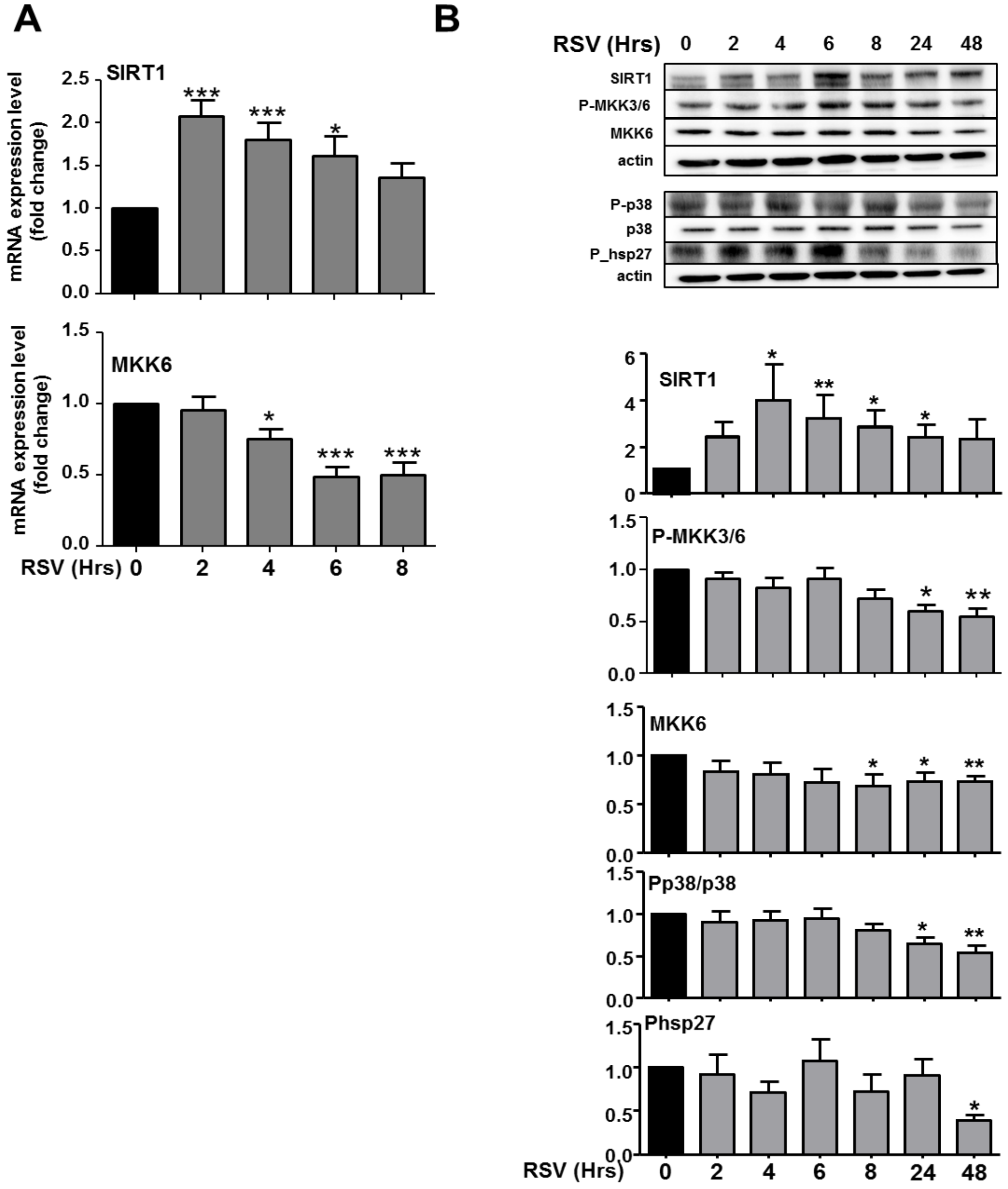

Figure S7. Resveratrol inhibits MKK6 expression and p38^{MAPK} activation.

PT-ECFC were treated with 1µM RSV or solvent alone (DMSO) for different times. **(A)** Time-dependent changes in the level of SIRT1 and MKK6 were determined by qRT-PCR analysis on a Stratagen MX3000. Changes in mRNA were normalized to the housekeeping gene *RPL13A,* as described in "Methods". Results were expressed as fold induction of mRNA level in RSV-stimulated PT-ECFC compared to DMSO-treated PT-ECFC. Data are means ± SEM of 8-10 independent samples. * *P*< 0.05, *** *P* < 0.001. **(B)** Time dependent modulation in SIRT1 and MKK6/p38MAPK pathways proteins in RSV-treated PT-ECFC. Each whole cell lysate (30µg) was resolved on 4-12% SDS-PAGE gradient under reducing conditions. Blots were probed with anti-SIRT1, P-MKK3/6, MKK6, Pp38, p38, PHsp27 polyclonal antibodies or with anti-β-actin polyclonal antibody as a loading control. Staining intensity was measured by densitometry and normalized to β-actin. Results were expressed as fold induction of protein expression in RSV-stimulated PT-ECFC compared with DMSO-treated PT-ECFC. Bars represent mean ± SEM of the relative intensities in 6-10 independent samples. * *P* < 0.05, ** *P* < 0.01.

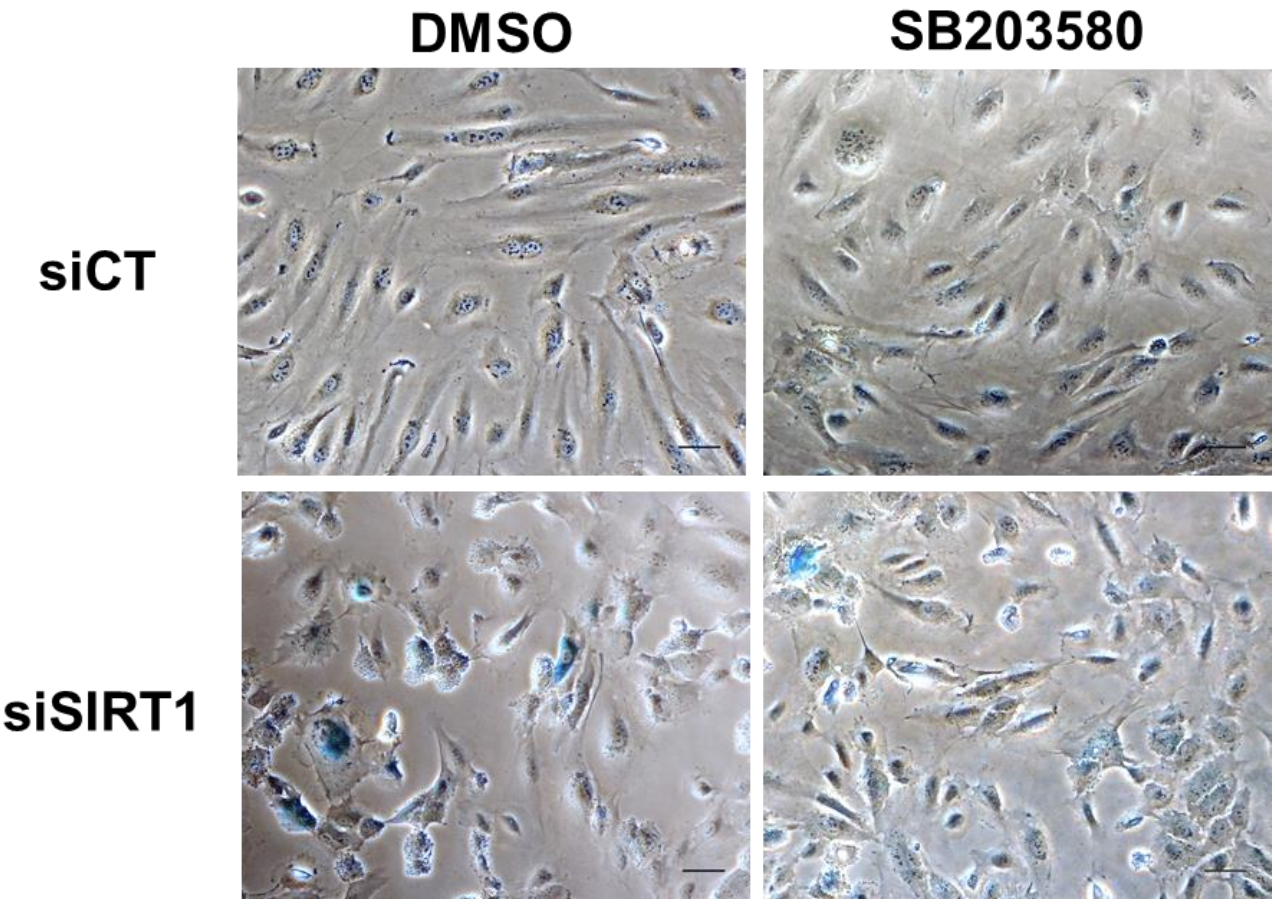

Figure S8. SB203580 treatment reverts CT-ECFC senescence induced by SIRT1 siRNA.

After transfection with siRNA control (siCT) or SIRT1 (siSIRT1), CT-ECFC were incubated in the presence or not of 2µM SB203580 for an additional 24h. SA-β-gal staining was performed for evaluation of the senescence status. Representative images of SA-β-gal staining are shown (original magnification x20, scale bar, 49µm).

Figure S9. Raw data

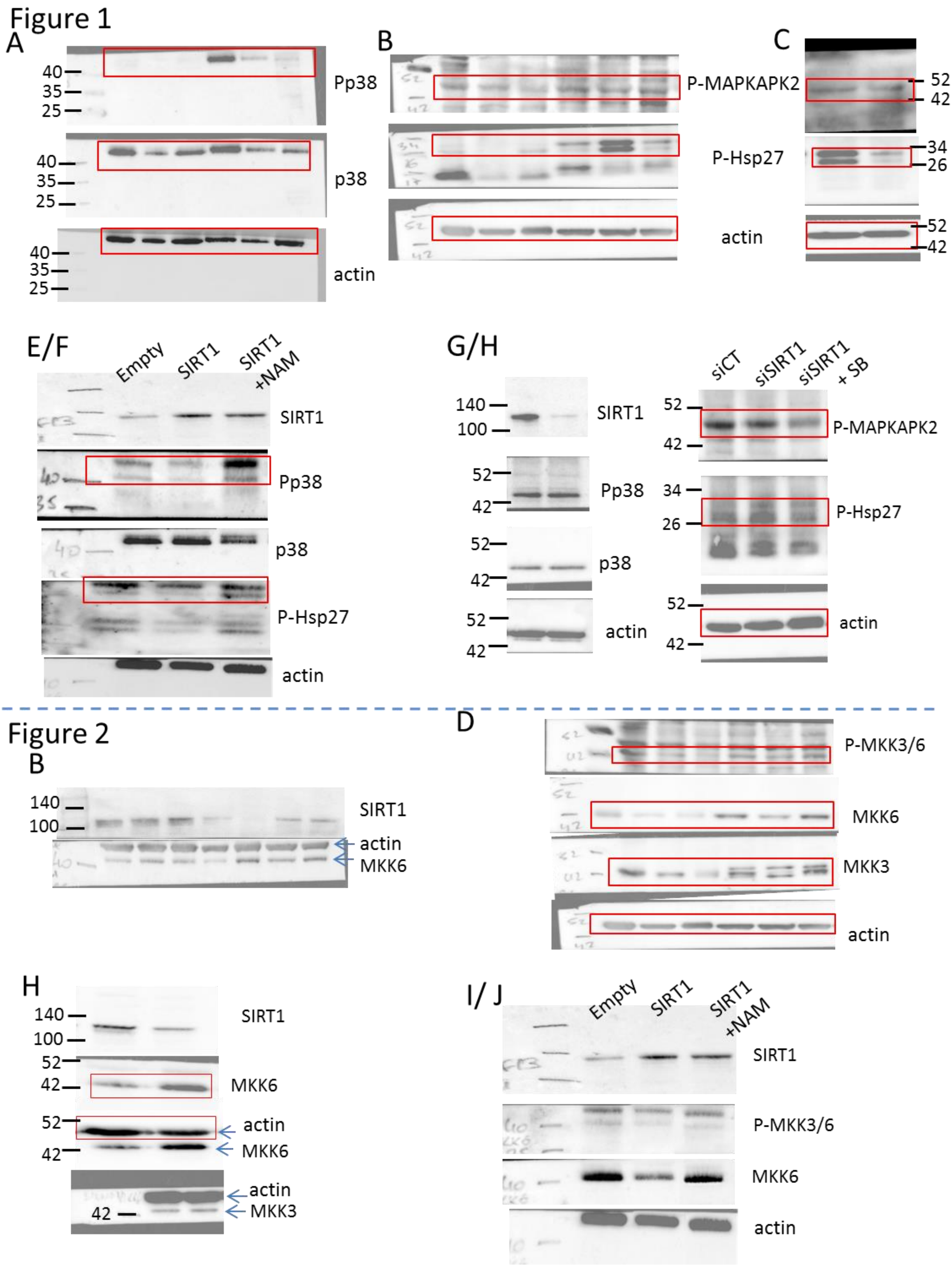

Table S1: Clinical characteristics of mothers and neonates.

Characteristics	Normal birth weight term neonates	Low birth weight preterm neonates	P value
Maternal data			
Number	18	28	
Age, years	32.67 ± 1.268	30.10 ± 1.130	NS*
Primigravida, n (%)	3 (17%)	8 (28%)	NS†
Primiparity, n (%)	4 (22%)	14 (50%)	NS†
Multiple pregnancies, n (%)	0 (0%)	5 (18%)	NS†
Antenatal steroid therapy, n (%)	0 (0%)	27 (96%)	< 0.0001†
HTA, Preeclampsia, n (%)	1 (5%)	3 (11%)	NS†
Premature rupture of membranes, n (%)	0 (0%)	16 (57%)	< 0.0001†
Cesarean section, n (%)	11 (61%)	23 (82%)	NS†
Infant data			
Number	18	29	
Mean gestational age (w)	39.44 ± 0.2321	31.07 ± 0.5160	< 0.0001*
Number by gestational age (w)			
≤ 28		4	
28-32		11	
32-36		14	
≥ 37	18		
Mean birth weight, (gr)	3547 ± 107.3	1573 ± 94.20	< 0.0001*
Number with birth weight, (gr)			
≤ 1,500		15	
1,510-2,000		7	
2,010-2,700		7	
≥ 2,700	18		
Male	10 (55%)	22 (75%)	NS†
Small for gestational, n (%)	0	4 (14%)	NS†

Significant differences were determined by: *: two-tailed unpaired t-test, †: the chi^2 test of Pearson. NS indicates not significant, No, number; and HTA, arterial hypertension

Table S2: Genes upregulated in PT-ECFC relative to CT-ECFC

ProbeName	Gene Symbol	FC (abs)	p (Corr)	p
A_23_P6335	SERPIND1	17.095173	7.678564E-4	4.89907E-4
A_24_P40306	SERPIND1	16.277954	8.1287115E-4	5.295698E-4
A_23_P78405	LIPG	7.2464876	0.0011209381	9.524144E-4
A_23_P399255	RNF182	6.728418	0.0010643232	8.838832E-4
A_24_P911179	ASPM	6.2638187	6.8285596E-4	3.787817E-4
A_23_P301530	ANK3	6.10681	0.0012896705	0.0012074136
A_32_P152348	HIST1H2BD	5.7422314	3.3914755E-4	6.579644E-5
A_23_P148753	PLEKHA6	5.5711737	7.25094E-4	4.1766724E-4
A_23_P202269	ANK3	5.2564063	0.0011772255	0.0010377819
A_32_P52386	lnc-EVX1-3	5.137981	5.863832E-4	2.751593E-4
A_23_P35871	E2F8	4.5342784	7.3597586E-4	4.3520908E-4
A_23_P258493	LMNB1	4.3706193	3.7883423E-4	1.05525716E-4
A_24_P237912	TCAF2	3.9284492	8.3580194E-4	5.704987E-4
A_23_P333998	POLQ	3.918786	0.0011399247	9.815297E-4
A_24_P43959	FRMD4A	3.908402	6.8132184E-4	3.6937266E-4
A_23_P381714	CA13	3.6760795	3.530762E-4	8.6045104E-5
A_24_P644742	HAUS6	3.6420453	1.4953452E-4	2.0408825E-6
A_32_P48842	SMIM10	3.636104	9.4853743E-4	7.30309E-4
**A_24_P416489**	**MAP2K6**	**3.4100718**	**9.960561E-4**	**7.9412217E-4**
A_24_P156781	PIK3R3	3.2312868	3.530762E-4	8.962758E-5
A_23_P48570	DHRS2	3.2053542	8.3226775E-4	5.5892766E-4
A_23_P366908	CCSAP	3.1233194	3.530762E-4	8.928014E-5
A_23_P22970	PIK3R3	3.1112447	5.753532E-4	2.3764157E-4
A_24_P272561	ZDHHC13	3.0996451	6.834655E-4	3.814329E-4
A_32_P24531	lnc-PPA2-1	3.0588953	1.4953452E-4	1.1823589E-6
A_24_P942328	DHFR	3.0569654	7.25094E-4	4.15049E-4
A_24_P171110	HNRNPU	3.0213485	7.4082275E-4	4.6583818E-4
A_32_P150891	DIAPH3	2.9907699	8.4099243E-4	5.823729E-4
A_24_P914240	ZNF44	2.9426215	3.9035646E-4	1.1437247E-4
A_23_P357270	SLC17A4	2.9420094	4.2885364E-4	1.4360248E-4
A_24_P298174	CBX1	2.8406188	4.3390074E-4	1.4798886E-4
A_32_P214925	TCAF2	2.8308308	3.1580793E-4	5.611166E-5
A_23_P398460	HK2	2.8111115	5.448165E-4	2.146999E-4
A_32_P99549	HNRNPM	2.7831109	0.0011772255	0.0010355201
A_23_P367071	UBE2DNL	2.779436	5.5608887E-4	2.2710214E-4
A_23_P254612	DBF4	2.7611766	9.357628E-4	7.098155E-4
A_23_P13065	ZDHHC13	2.7572293	3.7883423E-4	1.06675216E-4
A_23_P216396	EXOSC2	2.7101634	5.5608887E-4	2.280091E-4
A_32_P437876	LOC100133315	2.7028432	5.2417937E-4	2.0537323E-4
A_24_P269895	HNRNPA3	2.6903505	2.2160183E-4	2.4205963E-5
A_23_P107507	CBX1	2.6487443	6.472328E-4	3.2353928E-4

A_24_P509177	BBIP1	2.6450908	9.1396377E-4	6.677552E-4
A_24_P207195	IRX3	2.6363583	3.9035646E-4	1.1585139E-4
A_24_P7873	ELFN1	2.57549	4.3390074E-4	1.4838853E-4
A_23_P106874	PMFBP1	2.5639555	4.0550015E-4	1.2882068E-4
A_24_P374759	ERC2	2.5497077	7.4082275E-4	4.489376E-4
A_24_P462899	CENPW	2.5376422	0.0012781684	0.0011721656
A_24_P281923	DLAT	2.5170834	5.863832E-4	2.7445433E-4
A_23_P42042	LYRM2	2.5087917	3.3914755E-4	7.1503375E-5
A_23_P18123	NLGN1	2.4953845	4.6550616E-4	1.6329829E-4
A_24_P8098	NSD1	2.4857402	6.6392653E-4	3.4290564E-4
A_24_P221634	NSF	2.4809246	5.753532E-4	2.4155976E-4
A_24_P488588	HNRNPA3	2.4680638	1.9764541E-4	1.621892E-5
A_23_P94133	POP1	2.4363585	5.860619E-4	2.7100358E-4
A_23_P379020	GNRHR2	2.42962	6.8285596E-4	3.7953726E-4
A_23_P168747	NCAPG2	2.3888853	0.0012856561	0.0012007267
A_24_P488586	HNRNPA3	2.3774538	2.1504618E-4	2.1908027E-5
A_24_P264211	PTMA	2.3692524	2.1042408E-4	1.953397E-5
A_32_P194264	CHAC2	2.3672519	0.0013257653	0.0012955656
A_23_P348737	NR2F1	2.3437905	6.8244827E-4	3.7452832E-4
A_24_P134727	TFAM	2.3344986	2.2253957E-4	2.5926463E-5
A_23_P337464	LYRM7	2.3289406	6.932519E-4	3.9005288E-4
A_23_P30972	ASCC3	2.322726	5.753532E-4	2.5662556E-4
A_24_P273413	EML4	2.3222775	1.9351073E-4	1.4017561E-5
A_24_P41629	RPL21P44	2.3129685	1.4953452E-4	1.4046025E-6
A_23_P84189	PITPNC1	2.2808878	9.614214E-4	7.446088E-4
A_23_P160934	ANP32E	2.270519	6.8244827E-4	3.7620155E-4
A_23_P94461	FSD1L	2.2515883	0.0013146335	0.0012515283
A_23_P112673	ZWILCH	2.2462583	9.936622E-4	7.876866E-4
A_24_P238499	TYMSOS	2.2450159	8.3580194E-4	5.7259185E-4
A_24_P391811	DOCK10	2.2390528	8.3226775E-4	5.523558E-4
A_24_P350576	TNIK	2.2388287	3.3914755E-4	6.5607135E-5
A_32_P353677	ATAD3B	2.2353525	8.3580194E-4	5.749708E-4
A_23_P373119	HMGB3P1	2.227906	0.001164781	0.0010125949
A_23_P120270	MCFD2	2.2205472	6.8244827E-4	3.7323355E-4
A_23_P397978	TCAF2	2.218365	0.0013246301	0.0012872459
A_32_P79504	FANCM	2.2174225	0.0012856561	0.0011909598
A_23_P96072	GRIN1	2.2150614	9.3034335E-4	6.9934694E-4
A_23_P97123	ANKRD36BP1	2.1941137	0.0012856561	0.0011846541
A_24_P82630	SMCHD1	2.1919544	9.357628E-4	7.074582E-4
A_24_P712350	CHML	2.1908178	3.530762E-4	8.7268236E-5
A_24_P374427	ZDHHC21	2.1868622	8.3226775E-4	5.5307406E-4
A_24_P50829	TRPM7	2.1837566	0.0013303839	0.0013083046
A_32_P164649	SAFB	2.1782699	2.1042408E-4	2.0131689E-5
A_24_P45379	CACYBP	2.1771054	0.0010630856	8.790435E-4
A_24_P173727	PEX10	2.1547015	0.0010188104	8.2703354E-4
A_24_P829209	HOXA-AS3	2.1397355	0.0012220357	0.0010939864
A_23_P5616	RIF1	2.1391141	9.022291E-4	6.377997E-4
A_24_P89080	DCK	2.1365762	9.1396377E-4	6.8055966E-4

A_24_P336577	FAM212B	2.1347034	9.574881E-4	7.393815E-4
A_24_P286412	CRCP	2.130663	8.782624E-4	6.161841E-4
A_23_P349615	SMC5	2.1305435	1.5677472E-4	4.6675923E-6
A_23_P116123	CHEK1	2.128699	9.110757E-4	6.583915E-4
A_24_P267452	CD3EAP	2.1262093	3.3914755E-4	7.3155854E-5
A_23_P202258	TFAM	2.121912	5.753532E-4	2.4746277E-4
A_32_P233304	LIN9	2.1196601	0.0010894343	9.1323873E-4
A_23_P341275	POP1	2.1136708	6.2940526E-4	3.1255203E-4
A_23_P371876	ALKBH8	2.1096797	4.0550015E-4	1.2614539E-4
A_32_P229104	LOC102723559	2.1083193	3.3914755E-4	7.660952E-5
A_24_P219378	CASKIN1	2.1071038	2.2239047E-4	2.5329211E-5
A_32_P83776	CCSAP	2.0994794	6.6236826E-4	3.394826E-4
A_23_P346309	BAX	2.0951953	7.25094E-4	4.2118217E-4
A_23_P151405	CKAP2	2.0916264	0.0013369208	0.00133083
A_23_P102320	NUP35	2.0900977	0.0013273369	0.0013001249
A_24_P127174	lnc-KIAA1267-3	2.0797536	6.8069116E-4	3.6707328E-4
A_23_P250206	GCK	2.0780714	8.7093835E-4	6.090617E-4
A_24_P933492	ZDHHC21	2.054908	2.4230906E-4	3.1985604E-5
A_24_P196519	MTAP	2.0537927	0.0013146335	0.0012547413
A_32_P93149	DDX10	2.0534158	9.819334E-4	7.739156E-4
A_23_P122563	PFDN6	2.0455096	6.6392653E-4	3.4481834E-4
A_23_P54373	RAB27A	2.0413346	9.1396377E-4	6.846238E-4
A_24_P119002	FAM98A	2.0386882	7.941301E-4	5.119335E-4
A_23_P155197	PTPLB	2.0273967	8.3580194E-4	5.687583E-4
A_24_P941148	HAUS6	2.0237284	3.1473604E-4	5.0342693E-5
A_23_P135568	TNIK	2.0205886	7.445827E-4	4.715125E-4
A_24_P238878	DDX24	2.0162005	3.1473604E-4	5.154704E-5
A_24_P364296	STX2	2.0050175	4.7304126E-4	1.7088217E-4
A_32_P94866	WWTR1	2.0020678	6.027562E-4	2.883344E-4
A_24_P34632	PTMA	1.9997072	2.449662E-4	3.4596596E-5
A_24_P34476	RIF1	1.9985168	4.878963E-4	1.8138511E-4
A_24_P282172	TRIP11	1.997051	0.0010964718	9.216358E-4
A_23_P42036	LYRM2	1.9958657	9.022291E-4	6.391646E-4
A_23_P368718	SPRYD7	1.9913327	1.5677472E-4	5.4117845E-6
A_23_P149724	NUCKS1	1.9913033	9.952111E-4	7.9118146E-4
A_23_P500381	HTR7	1.9891075	0.0013322701	0.001320131
A_24_P366967	FNBP1L	1.9873269	6.242166E-4	3.0713165E-4
A_32_P119998	lnc-TUBA1C-1	1.9847481	5.753532E-4	2.5028284E-4
A_23_P411612	SPRYD4	1.9634136	0.0013203182	0.0012661822
A_24_P924389	BZW1	1.9352142	0.0012089367	0.0010712446
A_32_P98930	LLPH	1.9330993	3.893024E-4	1.11736E-4
A_23_P23151	CEP170	1.9317206	0.0010188104	8.364487E-4
A_24_P353486	KLHL4	1.9311978	4.3390074E-4	1.4924604E-4
A_23_P48387	PDS5B	1.9296118	2.2253957E-4	2.639571E-5
A_23_P162525	UTP20	1.926327	5.753532E-4	2.581881E-4
A_23_P116712	RASSF8	1.9261725	3.530762E-4	9.007867E-5
A_23_P357284	GPR4	1.9200838	0.0012856561	0.001188956
A_32_P1516	SH3D19	1.9183143	8.7093835E-4	6.075519E-4

A_24_P257108	TANK	1.9036129	0.001164781	0.0010135452
A_24_P103363	FAM172A	1.9028964	4.956784E-4	1.8630282E-4
A_24_P555473	MARCH3	1.8995186	9.4853743E-4	7.3026965E-4
A_23_P45140	KRAS	1.8973005	3.7883423E-4	1.0362308E-4
A_32_P109532	lnc-FAM105B-1	1.8927816	5.753532E-4	2.5190256E-4
A_24_P85169	BTBD9	1.8925081	0.0012341314	0.0011076259
A_32_P70468	KDELR2	1.891439	4.0550015E-4	1.2550993E-4
A_23_P146479	SURF2	1.8850158	5.5608887E-4	2.2471794E-4
A_32_P156136	NAA16	1.882977	7.4082275E-4	4.6411128E-4
A_23_P57413	PPM1F	1.8828828	0.001164781	0.0010060185
A_23_P46118	CHML	1.8802104	9.6478104E-4	7.494085E-4
A_23_P161156	ZNF438	1.8785765	0.0010894343	9.108004E-4
A_24_P927222	RBM27	1.8757873	3.6397288E-4	9.5345975E-5
A_24_P128524	ICMT	1.8726373	7.0888264E-4	4.0207693E-4
A_32_P80809	ACAA2	1.8682969	4.1581242E-4	1.3449969E-4
A_32_P57057	USP15	1.8596199	4.204519E-4	1.3966844E-4
A_24_P375453	SSBP3	1.8592755	3.3914755E-4	7.725457E-5
A_23_P113825	NACC2	1.8564368	5.838708E-4	2.6467035E-4
A_24_P213710	AHI1	1.8538904	9.696446E-4	7.5760385E-4
A_23_P217367	ATG4A	1.8519431	0.0012781684	0.0011700689
A_24_P405002	PDIK1L	1.8383238	1.5677472E-4	6.071003E-6
A_23_P82379	CACNA2D1	1.8381224	5.8364077E-4	2.632366E-4
A_24_P453740	DNAJC21	1.8335056	5.753532E-4	2.5065636E-4
A_23_P123974	DTYMK	1.8323935	9.110757E-4	6.5157964E-4
A_32_P326819	KRR1	1.8315505	6.472328E-4	3.2499005E-4
A_23_P18372	B3GNT5	1.8306806	3.5055724E-4	8.304773E-5
A_24_P791515	SYNCRIP	1.8277539	7.400307E-4	4.4165843E-4
A_23_P501080	ZNF92	1.8238966	3.0712617E-4	4.687347E-5
A_23_P23443	EFHD2	1.8216723	6.242166E-4	3.067936E-4
A_24_P161261	SPDYE2	1.819119	1.9240817E-4	1.0849721E-5
A_32_P63848	OXCT1	1.8136392	8.3226775E-4	5.5579917E-4
A_24_P196688	CARD8	1.8126081	0.0013020994	0.0012220158
A_24_P239664	BCKDHB	1.8062713	0.0013435701	0.0013405095
A_24_P827491	PA2G4	1.8039593	1.9764541E-4	1.6658041E-5
A_23_P78664	DDX39A	1.8020912	9.7270566E-4	7.622113E-4
A_32_P121303	NUP50	1.8000201	4.204519E-4	1.3983138E-4
A_32_P496952	RBM43	1.79621	7.25094E-4	4.1858893E-4
A_23_P421563	LSM3	1.7946393	3.3914755E-4	7.020066E-5
A_32_P9348	lnc-TSC1-1	1.7937293	5.2286015E-4	2.0025708E-4
A_32_P174713	SRP72	1.7891335	2.449662E-4	3.4385906E-5
A_23_P146997	TXLNG	1.7841176	0.0012856561	0.0012000077
A_24_P933503	MON2	1.7824017	1.9521249E-4	1.5118962E-5
A_32_P20904	LOC102725275	1.7700454	9.1396377E-4	6.7343353E-4
A_24_P16892	TAF2	1.7632846	3.7883423E-4	1.04737264E-4
A_23_P144999	RAPGEF6	1.7628253	7.360307E-4	4.3759457E-4
A_32_P101699	LOC729887	1.7582382	9.696446E-4	7.5600756E-4
A_23_P153084	RALBP1	1.7578359	6.666522E-4	3.492711E-4
A_23_P92281	GTPBP8	1.7551967	2.4230906E-4	3.1442145E-5

A_23_P204090	PTPN11	1.7521098	8.877035E-4	6.2483E-4
A_32_P68050	NEK1	1.7473773	3.3914755E-4	7.6256605E-5
A_23_P153855	ZNF492	1.7471088	6.083409E-4	2.9377738E-4
A_32_P8402	SYNCRIP	1.7466187	8.177051E-4	5.3644436E-4
A_24_P205627	TXLNA	1.7448064	0.0012551412	0.0011350594
A_23_P41970	AGGF1	1.7445552	5.753532E-4	2.4102253E-4
A_24_P184692	NKX2-1	1.7429115	8.3226775E-4	5.5844086E-4
A_23_P82474	TWISTNB	1.7406734	3.3680163E-4	6.242534E-5
A_24_P653603	C17orf89	1.7351823	2.0461227E-4	1.801082E-5
A_23_P216080	HOOK3	1.7329985	3.9035646E-4	1.171855E-4
A_23_P348857	CEP170	1.7253618	0.0012595624	0.0011447959
A_23_P144726	NOP16	1.7203113	0.0010188104	8.3137845E-4
A_23_P160598	TOE1	1.7142345	5.916913E-4	2.8095502E-4
A_23_P406355	FAM76B	1.7067972	7.988022E-4	5.1858457E-4
A_23_P74799	SLC25A24	1.7035702	0.0013246301	0.0012811148
A_24_P177987	DNAJC21	1.7021267	1.9240817E-4	8.418449E-6
A_23_P330727	PDDC1	1.7012873	3.3680163E-4	6.2910556E-5
A_24_P380991	RUFY2	1.7004983	7.973114E-4	5.1580055E-4
A_24_P330009	POLR2L	1.7000097	6.2580133E-4	3.0933687E-4
A_24_P290481	EZR	1.6998488	2.4230906E-4	3.2362423E-5
A_23_P8763	PTPN12	1.6997565	7.796705E-4	5.008362E-4
A_24_P911259	C1GALT1	1.6967083	8.3580194E-4	5.734466E-4
A_23_P259771	HEATR3	1.6896108	3.3914755E-4	7.653571E-5
A_23_P33022	POLR2L	1.6870428	2.4230906E-4	3.2781612E-5
A_23_P213661	PPIP5K2	1.683999	5.916913E-4	2.816736E-4
A_23_P165840	ODC1	1.6811627	9.1396377E-4	6.8443175E-4
A_23_P98382	TIMM8B	1.680723	9.1396377E-4	6.8495236E-4
A_24_P165656	PRKD3	1.6769959	1.9240817E-4	1.2272048E-5
A_23_P132863	LSM3	1.6739253	6.8069116E-4	3.6748018E-4
A_23_P202361	HPS1	1.6734138	7.007742E-4	3.9588154E-4
A_24_P264207	PTMA	1.6731312	1.5625464E-4	3.0455838E-6
A_24_P41170	PLEKHA5	1.6731274	5.179252E-4	1.9584416E-4
A_23_P58466	SMN1	1.6671002	0.0010089503	8.067006E-4
A_24_P85283	POLR3A	1.6660334	0.0010188104	8.242981E-4
A_24_P942370	GALNT4	1.6584125	7.678564E-4	4.914981E-4
A_23_P218619	MRPL30	1.6580487	6.6981174E-4	3.5611057E-4
A_24_P145229	CEP89	1.6498574	3.1473604E-4	5.125653E-5
A_24_P29001	LSM3	1.6342083	3.1496826E-4	5.5245004E-5
A_24_P284213	CASC4	1.6192081	2.4230906E-4	3.0250972E-5
A_24_P941336	TSR1	1.6165216	3.3914755E-4	6.939524E-5
A_32_P143516	FAM178A	1.6084676	5.753532E-4	2.509442E-4
A_23_P114466	TBL1Y	1.6059512	0.0012343524	0.0011119042
A_24_P398092	RAD23B	1.6030964	5.8406556E-4	2.6874998E-4
A_23_P103631	EBNA1BP2	1.5942426	0.0010479022	8.641015E-4
A_24_P226116	NAA15	1.5903966	0.0013040283	0.0012327374
A_23_P165247	DAZAP1	1.5888557	0.0011061478	9.3564397E-4
A_32_P1509	ZCCHC17	1.5885962	5.2286015E-4	2.032878E-4
A_24_P224219	EXOC5	1.5812374	7.4082275E-4	4.6522584E-4

A_23_P142918	C2orf69	1.5796496	7.407433E-4	4.4545837E-4
A_23_P401524	COX6A2	1.5773636	0.0011703165	0.0010221717
A_23_P116679	PA2G4	1.5717243	0.0013305083	0.0013146807
A_23_P65558	MGAT2	1.5701958	9.1396377E-4	6.787451E-4
A_23_P91769	NDUFA6	1.5629529	0.0013246301	0.001278083
A_24_P912818	ANKRD50	1.5604343	0.0012103982	0.0010752968
A_23_P90732	PNKD	1.5588175	3.1690695E-4	5.7028814E-5
A_24_P188071	TUBA1C	1.5587763	0.0010188104	8.377917E-4
A_32_P107797	FAM133CP	1.5541427	3.7883423E-4	1.0442723E-4
A_24_P337504	C5orf24	1.5508274	0.0010177682	8.1669755E-4
A_23_P97473	MRPL20	1.5404217	7.4082275E-4	4.5714376E-4
A_23_P115955	MRPL21	1.5392029	0.0013146335	0.0012466927
A_24_P158065	FAM133B	1.5383047	9.1396377E-4	6.813281E-4
A_23_P126291	SNRPE	1.5332049	2.1504618E-4	2.2338872E-5
A_24_P144504	BRCC3	1.5303979	0.0011061478	9.373279E-4
A_24_P230965	KIAA1586	1.5214591	3.7883423E-4	1.07868516E-4
A_24_P288979	GNPTAB	1.5178521	0.0013154031	0.0012584722
A_24_P4705	PPME1	1.5146358	9.110757E-4	6.5253937E-4
A_23_P300770	UBR1	1.5099536	0.0013534502	0.0013534502
A_23_P46725	EPC1	1.5085316	0.0013246301	0.0012838813

TableS3: Genes downregulated in PT-ECFC relative to CT-ECFC

ProbeName	Gene Symbol	FC (abs)	p (Corr)	p
A_23_P128574	ENOX1	7,480545	1.8134886E-4	5.4676344E-5
A_23_P132115	SIK1	6,473699	2.84636E-4	1.140676E-4
A_23_P205164	POU4F1	6,18772	1.0407757E-4	2.1244297E-5
A_32_P232883	LOC100507165	6,1080213	0.0011514068	0.0010072043
A_24_P191312	SLC1A4	5,6319747	8.889166E-4	6.8416615E-4
A_23_P143673	RASD2	5,597197	0.0010308647	8.4554066E-4
A_32_P158376	XLOC_l2_014504	5,449548	5.0709833E-4	2.972318E-4
A_24_P348006	RASD1	5,35677	0.0012274834	0.001149329
A_24_P27627	CHRNB1	5,3273754	0.001155831	0.0010400201
A_24_P91780	ADM2	4,775176	0.0010646742	8.8922225E-4
A_23_P42065	TNFRSF21	4,457057	3.1296673E-4	1.3245408E-4
A_23_P200303	HHLA3	4,2094502	2.4018642E-4	8.5459586E-5
A_24_P221414	DYNC1I1	3,7768717	7.7275874E-4	5.614801E-4
A_24_P316439	PLA2G4C	3,6613584	3.524967E-4	1.5983406E-4
A_24_P241815	JUNB	3,659653	6.161574E-5	7.893235E-6
A_24_P100228	XBP1	3,632505	3.358705E-5	2.1669705E-6
A_23_P142506	GADD45B	3,5963297	2.882939E-4	1.1607339E-4
A_23_P114299	CXCR3	3,595329	5.93987E-4	3.7100946E-4
A_23_P52161	NUAK2	3,5876007	5.1232695E-5	5.4686584E-6
A_24_P166527	TNFAIP3	3,5638201	1.9626711E-4	6.1746956E-5
A_24_P66592	CNKSR3	3,4225364	1.8628394E-4	5.6513105E-5
A_32_P213330	ARHGEF28	3,3868942	2.9441555E-5	1.4464405E-6
A_24_P42446	PURG	3,3492107	1.2230607E-4	2.748451E-5
A_23_P6596	HES1	3,2335005	2.0585267E-4	6.5854496E-5
A_23_P128817	PCK2	3,1930342	1.5264044E-4	3.9732247E-5
A_24_P317604	SLC37A3	3,1884117	8.708144E-7	1.6307385E-9
A_23_P108314	ADAMTS10	3,0990765	7.902579E-4	5.830742E-4
A_23_P127107	CLRN3	3,0985203	0.0012981272	0.001277676
A_24_P381975	TMEM231	3,0885332	2.772695E-5	1.0384625E-6
A_24_P372134	TMEM140	3,048863	3.1106852E-4	1.310682E-4
A_32_P223059	SLC45A1	2,9793122	4.3832755E-4	2.2983467E-4
A_23_P51699	ARHGEF2	2,9732327	2.1290686E-4	7.216506E-5
A_24_P570049	PPARA	2,973223	3.5280627E-4	1.6120735E-4
A_24_P920795	TMEM223	2,9566588	2.9441555E-5	1.3604681E-6
A_23_P135769	ACTB	2,9529712	8.836054E-5	1.3899411E-5
A_23_P170246	OCRL	2,9477766	2.5986159E-5	8.2731106E-7
A_23_P412389	FGF18	2,9228559	0.0010167885	8.301869E-4
A_23_P214803	SNAP91	2,9053795	5.9502886E-4	3.7440017E-4
A_24_P417036	PPARA	2,8909237	4.2198863E-4	2.1725702E-4
A_23_P200960	ERMAP	2,8625033	0.0010625293	8.849543E-4
A_23_P201587	SORT1	2,8618124	0.0011343106	9.817801E-4
A_32_P97763	PPP1R16B	2,8584619	8.5538055E-4	6.502158E-4
A_32_P44316	EEF1A1	2,8531308	4.4666097E-4	2.350407E-4
A_24_P397043	ST6GAL1	2,7776785	1.4589152E-4	3.7155893E-5

A_23_P384023	GLIS3	2,765722	0.0010746553	9.014028E-4
A_23_P217688	TSC22D3	2,7627947	0.0010234225	8.375199E-4
A_32_P117812	LYSMD4	2,7577853	5.2895142E-5	6.169608E-6
A_23_P158449	SLC37A3	2,7506735	2.9441714E-4	1.19704506E-4
A_24_P935125	ZNF23	2,749866	3.524967E-4	1.6040579E-4
A_24_P937915	PRKCH	2,748175	2.8380298E-5	1.1160791E-6
A_24_P272290	PXDC1	2,7409985	1.5559593E-4	4.3415344E-5
A_23_P37503	MYO1E	2,7305424	7.3244964E-4	5.148376E-4
A_23_P33511	SCX	2,715189	0.0010055621	8.134885E-4
A_23_P218884	DVL3	2,6587474	1.730137E-4	5.0434282E-5
A_24_P370946	CYR61	2,639288	1.09559965E-4	2.297887E-5
A_24_P185186	SAMD14	2,6372857	5.913919E-4	3.654669E-4
A_24_P682285	HSPA1A	2,6331058	9.6326316E-5	1.637798E-5
A_24_P372932	PEX5	2,6207218	4.6099845E-5	4.316465E-6
A_24_P291588	DVL3	2,6051123	2.0218536E-4	6.39875E-5
A_24_P147252	ZNF23	2,59498	5.362388E-6	3.0125776E-8
A_24_P144773	RNF145	2,587716	9.642507E-4	7.63651E-4
A_23_P385673	RASA3	2,5801013	2.5986159E-5	8.286214E-7
A_23_P131308	CYP27A1	2,552509	3.7209535E-4	1.766002E-4
A_23_P29830	CBLB	2,5521953	6.161574E-5	7.961585E-6
A_24_P933704	PAM	2,5506768	1.730137E-4	5.0664894E-5
A_23_P76823	ADSSL1	2,5501926	0.0012229048	0.0011404618
A_23_P373598	MAFK	2,5319319	0.0010886859	9.2558697E-4
A_32_P88120	YPEL1	2,5253851	5.074653E-4	2.9839718E-4
A_23_P4821	JUNB	2,5148609	8.529834E-5	1.2778778E-5
A_23_P256487	CD274	2,5147448	5.4566876E-4	3.2801437E-4
A_32_P407293	PRR18	2,5076456	4.1923643E-4	2.0832616E-4
A_23_P162238	RAB5B	2,4972672	6.5583176E-6	4.9125976E-8
A_24_P283535	ARHGEF10	2,487203	3.9512955E-4	1.9047497E-4
A_23_P66050	B3GNT9	2,4779904	2.8468423E-5	1.1728564E-6
A_24_P919330	FTH1	2,47085	3.5454295E-4	1.6332878E-4
A_23_P91859	TRANK1	2,434512	0.0011082354	9.5295365E-4
A_32_P827528	S1PR2	2,4309022	2.918586E-4	1.1805516E-4
A_23_P63521	LCE2C	2,4298964	2.544333E-4	9.624631E-5
A_24_P127292	TTC7B	2,4225953	2.5986159E-5	6.679277E-7
A_24_P124349	PDGFD	2,4200475	4.2198863E-4	2.1731625E-4
A_24_P925124	MBD1	2,3989708	9.172234E-4	7.162587E-4
A_24_P357465	TP53INP2	2,3582149	0.0013554527	0.0013541079
A_24_P201153	TJP2	2,3556192	4.7269178E-4	2.6644237E-4
A_23_P31046	MYCT1	2,343127	1.9475345E-4	6.090604E-5
A_24_P123616	HSPA1A	2,3408558	1.8685451E-4	5.738603E-5
A_24_P239988	ITSN2	2,3102925	0.001155831	0.001033089
A_24_P339071	CDR2	2,3098276	1.9425509E-4	6.002264E-5
A_23_P31177	TMEM140	2,3083649	8.3549914E-4	6.2740664E-4
A_23_P54918	LDHD	2,3009398	7.8686344E-4	5.7467556E-4
A_24_P131718	RAB6B	2,2738302	2.77001E-4	1.0737679E-4
A_24_P187993	ELMO2	2,273759	9.6060525E-5	1.58302E-5
A_24_P714134	HNRNPKP3	2,2728374	5.6446162E-5	6.793196E-6

A_24_P76210	KANTR	2,269807	2.9441555E-5	1.3082043E-6
A_23_P256735	CPQ	2,2685854	2.562731E-4	9.742217E-5
A_24_P192978	KLHL35	2,268482	0.0012309954	0.0011594547
A_32_P79610	CLK2	2,2667768	9.7752294E-5	1.7207332E-5
A_24_P252033	SERAC1	2,2558038	5.181066E-4	3.056247E-4
A_32_P144596	TNKS	2,2518804	8.037247E-5	1.17397985E-5
A_23_P66924	DYM	2,2460222	2.1771486E-4	7.501785E-5
A_24_P295609	FUT4	2,245506	0.0012453897	0.0011879082
A_23_P340728	PSEN1	2,2381775	7.680253E-5	1.1074521E-5
A_23_P3131	NEK9	2,237839	1.4144073E-4	3.3615397E-5
A_23_P158933	GPR125	2,2367287	4.4563607E-5	4.0057175E-6
A_23_P62881	SGIP1	2,2289076	2.8070694E-4	1.1039037E-4
A_23_P117782	LARP6	2,2216334	6.8434596E-4	4.6466995E-4
A_24_P413470	TP73	2,2209978	6.8434596E-4	4.6329838E-4
A_23_P73708	ZNF75D	2,2185698	1.2230607E-4	2.7348466E-5
A_24_P15702	PANDAR	2,214858	1.0407757E-4	2.1206564E-5
A_24_P211689	CXorf38	2,2118044	0.0011343106	9.856182E-4
A_23_P46690	TMEM81	2,2117221	8.259023E-4	6.170579E-4
A_24_P117902	OSBPL10	2,2073464	4.2198863E-4	2.159314E-4
A_24_P3761	ALDH5A1	2,2063808	0.001155831	0.0010411137
A_23_P154667	FKBP1A	2,2024658	2.748125E-5	9.777973E-7
A_32_P47701	EEF1A1	2,2020783	2.5986159E-5	8.759379E-7
A_23_P365719	TAPBP	2,1929936	7.88132E-4	5.785538E-4
A_24_P609382	C17orf51	2,1892917	2.8070694E-4	1.0944429E-4
A_24_P303815	UHRF2	2,1834521	9.869043E-4	7.891538E-4
A_23_P109034	SDC4	2,1759357	0.0011343106	9.841473E-4
A_24_P353740	CTTNBP2NL	2,169488	1.150854E-5	1.336069E-7
A_24_P341370	PPP2R5C	2,1689076	2.8070694E-4	1.10134264E-4
A_32_P146286	LOC100130331	2,1642342	2.242643E-5	3.9371213E-7
A_23_P201059	UBQLN4	2,1603963	1.252297E-4	2.861053E-5
A_24_P68649	RNPEP	2,1593695	1.15066185E-4	2.478017E-5
A_23_P127608	TMPRSS4	2,159062	5.295438E-4	3.1336298E-4
A_24_P140171	CRTAP	2,1557136	3.8233335E-5	2.7352398E-6
A_23_P75811	SLC3A2	2,155438	1.5341557E-4	4.243902E-5
A_23_P89621	CBX4	2,1525052	1.00626814E-4	1.9421768E-5
A_23_P148410	FTHL17	2,1501806	0.0012309604	0.0011548898
A_23_P356041	SPAG9	2,1471229	8.5818104E-4	6.5568887E-4
A_24_P11307	AHCYL1	2,1416752	3.8233335E-5	2.6830671E-6
A_23_P323094	PHC1	2,13413	8.810803E-5	1.3694694E-5
A_24_P378368	MBTPS1	2,1284413	2.242643E-5	4.8637025E-7
A_23_P169934	RILPL1	2,1262994	6.8434596E-4	4.643462E-4
A_23_P29422	GYG1	2,1210048	3.025269E-4	1.2466975E-4
A_23_P46964	HIF1AN	2,1116934	2.1532048E-4	7.3789604E-5
A_24_P255233	RAMP2-AS1	2,111077	0.0010167885	8.290961E-4
A_24_P121535	ACTC1	2,1104715	1.0695218E-4	2.2231634E-5
A_23_P33539	ADCY2	2,1067557	3.358705E-5	2.1658486E-6
A_23_P146576	GBGT1	2,1022177	0.0011638909	0.0010549123
A_23_P91640	ASPHD2	2,101509	8.0215564E-4	5.94857E-4

A_23_P410998	RAB5B	2,101338	1.0067404E-4	2.0048195E-5
A_24_P12065	CCNG2	2,095304	3.4757765E-4	1.5686557E-4
A_24_P59220	POTEF	2,0857255	2.9441555E-5	1.5097619E-6
A_24_P162172	SPG11	2,0766723	4.6855904E-4	2.59186E-4
A_23_P379550	YARS	2,0750136	1.4226987E-4	3.506023E-5
A_23_P378722	SAT1	2,0691595	0.0011960202	0.001104191
A_23_P426944	PAX9	2,062347	0.0012659018	0.0012161192
A_24_P59278	DSTYK	2,053165	4.2471953E-4	2.1951795E-4
A_23_P390704	STK38	2,0510254	5.743355E-5	7.206082E-6
A_24_P23034	ZNFX1	2,0502167	3.9567807E-4	1.9265224E-4
A_23_P100011	AP3S2	2,0495384	4.71301E-4	2.6301068E-4
A_23_P213369	KIAA0141	2,04376	1.2241534E-4	2.7738308E-5
A_24_P179044	SNX9	2,0424855	4.5774344E-4	2.4541953E-4
A_24_P6903	ACTBL2	2,0381093	2.2060401E-4	7.6426484E-5
A_23_P209183	COLGALT1	2,0339823	0.0012981272	0.0012786796
A_23_P138826	SYVN1	2,0213723	5.070476E-4	2.9532315E-4
A_23_P386509	GATAD2B	2,0175505	4.1923643E-4	2.126287E-4
A_23_P210686	ZCCHC3	2,0169592	9.6321554E-4	7.59389E-4
A_24_P33077	HOXB2	2,0165265	3.358705E-5	2.1800388E-6
A_24_P390833	MPPE1	2,010886	4.0847757E-5	3.2892387E-6
A_23_P3819	ZNF747	2,0089178	3.854821E-5	2.8937525E-6
A_24_P18190	HSPA5	2,0084343	1.0986148E-4	2.3247841E-5
A_23_P39237	ZFP36	2,007413	3.7209535E-4	1.769215E-4
A_24_P125353	CASC3	2,005098	1.4395443E-4	3.6123394E-5
A_23_P143242	CEBPB	2,0018227	0.0011739659	0.0010684408
A_24_P328320	GORASP2	1,9944365	7.6961285E-4	5.5775314E-4
A_24_P276628	PPT1	1,9934208	3.9512955E-4	1.9107388E-4
A_23_P19437	PPP1R11	1,9911178	4.6904717E-4	2.6087454E-4
A_32_P231617	TM4SF1	1,9853235	1.5313512E-4	4.1868403E-5
A_23_P48246	PEX5	1,9823297	9.6326316E-5	1.6071235E-5
A_23_P255812	SARAF	1,9813852	8.3549914E-4	6.2717666E-4
A_24_P267522	ZNF585A	1,980507	0.0013008508	0.0012837984
A_23_P135271	B4GALT1	1,97921	3.025269E-4	1.2631799E-4
A_24_P17710	KLHL5	1,9774275	4.1923643E-4	2.1256128E-4
A_24_P190424	RAB8A	1,9681767	4.736314E-5	4.700836E-6
A_24_P54485	CCDC115	1,9667315	1.7576655E-6	6.583017E-9
A_24_P282031	TXNDC11	1,9658815	9.412493E-5	1.5334961E-5
A_23_P203505	TRIM5	1,9647028	2.439342E-4	8.897012E-5
A_23_P85888	CLK2	1,961261	2.145522E-4	7.312453E-5
A_23_P6474	JOSD1	1,9583894	2.1218098E-5	3.1787414E-7
A_23_P211227	POFUT2	1,9569905	6.8434596E-4	4.6520148E-4
A_24_P19662	LRRC47	1,953327	2.8409224E-4	1.1278569E-4
A_23_P435521	TMEM106A	1,9518306	0.0010625293	8.822837E-4
A_23_P202510	ASB13	1,9510617	7.09686E-4	4.89072E-4
A_24_P73389	STK24	1,9490496	5.2895142E-5	6.118398E-6
A_24_P67027	CLK2	1,9486781	2.6139963E-4	1.0035005E-4
A_24_P172990	AARS	1,948033	5.070476E-4	2.949432E-4
A_24_P881527	CTNND1	1,9453429	9.412493E-5	1.5315682E-5

A_23_P162199	PIP4K2C	1,9446573	1.7077866E-4	4.8930964E-5
A_24_P247931	FBXO25	1,9438905	3.364182E-4	1.4728234E-4
A_23_P95330	ZDHHC9	1,9421595	2.2627845E-4	7.881609E-5
A_23_P34510	PHC2	1,9383764	7.575678E-4	5.4313894E-4
A_23_P84555	KCTD21	1,9381267	1.00626814E-4	1.9455185E-5
A_23_P130130	BCL6B	1,9381082	8.995145E-4	6.973764E-4
A_23_P69121	SIAH2	1,9337629	1.7458819E-4	5.1984123E-5
A_23_P54846	HERPUD1	1,9336538	1.691365E-4	4.8143724E-5
A_23_P202156	NFKB2	1,9311929	0.0012347014	0.0011745848
A_23_P25994	LGMN	1,9297384	0.0010373783	8.52826E-4
A_24_P50875	ANKHD1	1,9289845	9.4145344E-4	7.38706E-4
A_24_P93251	EIF4B	1,9272127	7.863808E-4	5.728504E-4
A_32_P122402	LOC441455	1,9233975	4.985719E-5	5.135104E-6
A_23_P51361	TXLNA	1,9222863	5.2895142E-5	6.1918176E-6
A_24_P597190	CTD-2151A2.1	1,9213939	3.5690336E-4	1.6508451E-4
A_24_P302332	NECAP2	1,9206079	3.358705E-5	2.1285634E-6
A_23_P86054	WDTC1	1,9175134	0.0012889589	0.001255166
A_23_P120899	CSF2RB	1,9148889	8.90337E-4	6.8692665E-4
A_23_P76743	ASPG	1,9141457	0.0011514068	0.0010053529
A_23_P14939	TXNDC11	1,9118091	9.7752294E-5	1.6919745E-5
A_23_P106174	PSEN1	1,9107677	8.810803E-5	1.3540001E-5
A_23_P113317	P4HTM	1,9095345	6.706006E-5	8.916225E-6
A_23_P435501	SERINC3	1,9038092	1.150854E-5	1.5086101E-7
A_23_P171054	ELK1	1,8980042	1.5313512E-4	4.0909694E-5
A_24_P401709	CARKD	1,897275	3.358705E-5	2.135534E-6
A_23_P121011	CSRNP1	1,8964891	4.78697E-4	2.743332E-4
A_32_P163169	VDAC1	1,8947449	1.730137E-4	5.0412305E-5
A_23_P216894	MAPKAP1	1,8916736	2.5986159E-5	8.2539816E-7
A_23_P128036	MARS	1,8857995	2.9441714E-4	1.20672375E-4
A_24_P173124	FLCN	1,8837814	1.00626814E-4	1.924659E-5
A_32_P214943	PCBP2	1,8822386	4.719266E-5	4.5071642E-6
A_23_P210521	SLC2A10	1,8706542	4.7140807E-4	2.639532E-4
A_23_P77228	CRTC3	1,8694117	7.3244964E-4	5.1446835E-4
A_23_P149281	EPHA2	1,8687803	5.9502886E-4	3.7416347E-4
A_32_P73775	TP73-AS1	1,8676465	0.001086326	9.135973E-4
A_24_P89843	CYHR1	1,866512	0.0013215935	0.0013067442
A_23_P205228	ATP7B	1,8633057	4.7650153E-4	2.719754E-4
A_23_P65442	IRF9	1,8626542	0.0010625293	8.854411E-4
A_23_P381976	OGT	1,8612378	4.6556006E-4	2.5544775E-4
A_24_P38930	CTNND1	1,8569378	2.0585267E-4	6.674068E-5
A_23_P155351	BTD	1,848394	7.481299E-4	5.323771E-4
A_23_P355455	TBC1D5	1,8458713	4.6590838E-4	2.5651135E-4
A_32_P41106	CBR3-AS1	1,8416525	0.0010882126	9.231466E-4
A_23_P12010	RRNAD1	1,8405184	9.412493E-5	1.5077935E-5
A_23_P161231	SFXN3	1,8377266	1.4589152E-4	3.704822E-5
A_24_P174257	CCDC93	1,8372182	5.93987E-4	3.7045212E-4
A_23_P367899	EPOR	1,831957	9.424835E-4	7.4127916E-4
A_23_P33482	UVRAG	1,8308985	6.5420143E-4	4.2951186E-4

A_24_P276791	LRRC42	1,8237276	1.9437674E-4	6.042423E-5
A_23_P12950	KBTBD4	1,8236737	4.2865184E-4	2.2296552E-4
A_24_P316005	RABGAP1L	1,8235227	2.4302263E-4	8.783402E-5
A_24_P63118	PDCD6IP	1,8233502	0.0010167885	8.264517E-4
A_23_P201086	ARF1	1,822161	5.1232695E-5	5.449803E-6
A_24_P73264	DUSP16	1,8214103	4.1740912E-4	2.0524353E-4
A_23_P107206	STAT3	1,8211828	1.4144073E-4	3.3676984E-5
A_24_P918436	CSNK1E	1,8208351	8.7387016E-5	1.3255334E-5
A_23_P210538	ELMO2	1,81735	2.9441555E-5	1.4118847E-6
A_23_P217054	DCAF10	1,8169227	4.7269178E-4	2.6641073E-4
A_23_P135437	EXOC4	1,8168133	4.1923643E-4	2.0774643E-4
A_23_P63243	C1orf43	1,8159266	5.3944497E-4	3.2023233E-4
A_24_P279172	B3GNT9	1,8138117	3.9512955E-4	1.9164523E-4
A_23_P159395	AXIN2	1,8132423	0.0011514068	0.0010155666
A_23_P98763	LRTOMT	1,8103411	7.3244964E-4	5.1329803E-4
A_23_P101319	ZNF285	1,808693	5.838363E-4	3.5861105E-4
A_32_P532216	QARS	1,8073815	6.360587E-4	4.0974567E-4
A_23_P64404	FADS3	1,8053316	1.7389159E-4	5.145107E-5
A_24_P612446	C6orf89	1,8043545	1.867935E-4	5.7017493E-5
A_23_P24709	OSBP	1,8039817	4.0847757E-5	3.2783012E-6
A_24_P82493	DCAKD	1,8037784	2.3371313E-4	8.184336E-5
A_23_P500905	ZNF19	1,7983222	7.2878075E-4	5.058583E-4
A_23_P205389	MOAP1	1,7974913	3.4080376E-4	1.512556E-4
A_24_P259490	ARF1	1,7962564	2.242643E-5	5.039647E-7
A_23_P407142	LUZP1	1,791848	1.4787295E-4	3.821436E-5
A_23_P60286	EIF4B	1,7914662	0.0011960202	0.0011027604
A_23_P359897	KDM7A	1,7894821	7.3276693E-4	5.17328E-4
A_23_P135499	CLIC4	1,7894511	8.5538055E-4	6.5194734E-4
A_24_P204358	PYCR1	1,7888808	1.4226987E-4	3.516783E-5
A_23_P204252	M6PR	1,7887013	0.0012347014	0.0011744222
A_23_P202307	INPP5A	1,7863646	6.937952E-4	4.7292406E-4
A_24_P28367	COPA	1,7862245	5.2895142E-5	5.914017E-6
A_32_P15421	RNF185	1,7849314	4.6099845E-5	4.257851E-6
A_24_P325107	FAM160B1	1,7840331	1.00626814E-4	1.943422E-5
A_23_P201963	PARD3	1,7839646	7.545368E-4	5.383493E-4
A_23_P143083	TMEM214	1,7836808	1.730137E-4	5.0867322E-5
A_32_P15320	EEF1A1	1,7806364	4.418006E-5	3.7026607E-6
A_23_P210969	PHF20	1,7798828	4.430445E-5	3.816488E-6
A_23_P409541	POLR1D	1,7784699	2.0585267E-4	6.724103E-5
A_23_P371787	SUSD6	1,7782441	5.93987E-4	3.706902E-4
A_23_P325631	SKI	1,7731348	7.088077E-4	4.8449886E-4
A_23_P89762	PHLPP1	1,7731153	7.3244964E-4	5.1573233E-4
A_32_P140706	PMS2	1,7710453	1.4144073E-4	3.3143046E-5
A_24_P345822	TFG	1,7697573	3.8233335E-5	2.7923222E-6
A_23_P91221	PKIG	1,7696	2.439342E-4	8.90771E-5
A_24_P164838	SPATA2	1,7686787	4.3832755E-4	2.29112E-4
A_23_P148015	AXIN2	1,7681879	8.9683547E-4	6.9361995E-4
A_24_P206758	SNX15	1,7662151	1.4144073E-4	3.4384644E-5

A_23_P128541	TRAFD1	1,7655689	4.1923643E-4	2.1193392E-4
A_24_P793483	LOC101927733	1,764476	5.403187E-4	3.2176284E-4
A_23_P41359	ZNF330	1,7604369	1.0067404E-4	2.0172514E-5
A_23_P213471	LPCAT1	1,7593863	5.8410305E-4	3.5986872E-4
A_24_P116805	STAT3	1,7591088	0.001086326	9.1805076E-4
A_23_P158239	SHMT2	1,7589165	2.111384E-4	7.0774855E-5
A_24_P272967	AVL9	1,7585057	0.0012242155	0.0011439767
A_32_P453321	C1orf228	1,7572922	7.6961285E-4	5.569318E-4
A_24_P15062	ZNF490	1,7538105	0.0012759074	0.001233504
A_24_P357056	TANGO2	1,7525969	4.4563607E-5	3.972984E-6
A_23_P46627	ADIPOR1	1,7525086	1.5313512E-4	4.0498686E-5
A_24_P218970	EIF4B	1,7510335	6.5348606E-4	4.2709106E-4
A_23_P26878	NDEL1	1,7508434	5.741157E-5	7.0958117E-6
A_23_P27040	TMEM98	1,7455859	0.0011514068	0.0010150522
A_23_P15299	WBP2	1,7452306	4.7650153E-4	2.7215912E-4
A_23_P205293	EXD2	1,7434757	6.457898E-4	4.1722372E-4
A_23_P151544	DDX24	1,7426411	6.481439E-5	8.4962685E-6
A_24_P198217	SNX15	1,7399421	8.4486813E-4	6.3602434E-4
A_23_P57877	ALAS1	1,7390227	4.8251217E-4	2.783029E-4
A_23_P139998	UBAC2	1,7377554	6.5268413E-4	4.253447E-4
A_24_P233960	SNX27	1,7368815	6.9625E-5	9.525519E-6
A_24_P928901	SLC31A1	1,7362324	5.800326E-4	3.551885E-4
A_23_P77440	NFATC3	1,7359658	2.4777706E-4	9.187239E-5
A_24_P929268	TTBK2	1,7354251	5.4472644E-4	3.2642783E-4
A_24_P393461	C1orf43	1,7302809	5.730432E-4	3.4876223E-4
A_32_P209624	MKRN7P	1,7253661	3.3174988E-4	1.4350978E-4
A_24_P210637	CHST14	1,7241061	4.609739E-4	2.5206813E-4
A_23_P92140	MYD88	1,724	3.358705E-5	2.2308884E-6
A_23_P257694	GTF2IRD1	1,723422	0.0010905329	9.3124155E-4
A_23_P213592	RNF44	1,7229316	0.001155831	0.0010389157
A_23_P3169	GMPR2	1,7226045	3.025269E-4	1.2549618E-4
A_24_P174550	RHOA	1,7224958	2.4018642E-4	8.529574E-5
A_23_P63010	CERS2	1,7179711	1.00626814E-4	1.8681043E-5
A_24_P337388	MARCH6	1,7170619	5.2895142E-5	6.2404383E-6
A_23_P251680	COMT	1,7147019	2.7187477E-4	1.0488053E-4
A_24_P252602	NCS1	1,7106895	6.2147743E-4	3.9686105E-4
A_24_P297166	SEPT5	1,7102246	0.0012194351	0.0011349424
A_24_P194730	ZSCAN22	1,7071207	6.2048837E-4	3.950675E-4
A_23_P348323	PCGF2	1,7013506	4.609739E-4	2.5178058E-4
A_32_P129950	NHLRC3	1,6997452	9.772618E-4	7.796134E-4
A_23_P331253	XPNPEP1	1,6982361	2.6046645E-4	9.950404E-5
A_24_P255338	SEC24C	1,6971636	3.358705E-5	2.2642955E-6
A_24_P263595	SNX29P2	1,6970246	6.6594424E-4	4.4146867E-4
A_24_P172304	ADAMTS13	1,6958613	4.8805727E-4	2.8241516E-4
A_24_P370670	ZMYM6NB	1,6954207	2.0585267E-4	6.784657E-5
A_23_P385081	SCAMP2	1,6907806	4.1923643E-4	2.1194322E-4
A_23_P81399	SQSTM1	1,6903305	0.0011082354	9.5531804E-4
A_23_P66715	PIGS	1,6886516	2.0585267E-4	6.602475E-5

A_23_P114172	TBC1D8B	1,6873515	2.8202668E-4	1.1143751E-4
A_24_P376322	MBD1	1,6855065	4.5697458E-4	2.4389093E-4
A_23_P26810	TP53	1,6851417	8.0145587E-4	5.928372E-4
A_23_P419277	MARCH8	1,68392	0.0012309954	0.0011612904
A_23_P23421	ARHGEF11	1,6836736	4.1740912E-4	2.055779E-4
A_23_P78383	C18orf8	1,6834366	1.00626814E-4	1.9576491E-5
A_23_P84922	HDAC8	1,6822225	1.0589791E-4	2.1814176E-5
A_23_P428835	PHF20	1,6804863	6.9625E-5	9.444901E-6
A_24_P207479	DEDD2	1,6793576	0.0011514068	0.0010112457
A_23_P117506	DHRS7	1,6789476	0.0011960202	0.0010987946
A_24_P940537	STK35	1,678886	3.6444032E-4	1.6935471E-4
A_23_P200767	TROVE2	1,6779115	4.5377194E-4	2.4048213E-4
A_24_P39508	CMTM3	1,6774877	4.7650153E-4	2.7082366E-4
A_23_P99186	UBE3B	1,6772497	9.3625684E-4	7.328752E-4
A_32_P22338	PDCD6IP	1,6752517	0.0012903799	0.0012613826
A_23_P305938	ZNF75D	1,67313	4.609739E-4	2.513074E-4
A_24_P69654	KLF6	1,6703315	3.4125536E-4	1.5209509E-4
A_24_P163405	TRIM27	1,6693412	1.6042136E-4	4.5178367E-5
A_24_P28845	ZNF2	1,6690451	6.8434596E-4	4.6272494E-4
A_23_P206474	TRAF7	1,6671985	4.78697E-4	2.7520597E-4
A_32_P70220	CHMP7	1,667083	2.1183983E-4	7.1406685E-5
A_24_P74981	KDM1A	1,6612755	2.3801083E-4	8.3794075E-5
A_23_P140050	SUPT20H	1,6586076	2.9441555E-5	1.543752E-6
A_24_P298013	GTPBP2	1,6584241	4.7650153E-4	2.7072616E-4
A_23_P4913	KDELR1	1,6581595	2.0585267E-4	6.6583605E-5
A_24_P205213	ARSB	1,6573284	0.0013549939	0.0013473816
A_23_P73593	MSN	1,656895	4.2865184E-4	2.2315583E-4
A_23_P76515	CERS5	1,6552289	0.001155831	0.0010256722
A_23_P424513	RANBP9	1,6543504	3.364182E-4	1.4741921E-4
A_23_P137403	LRRC41	1,6540321	3.2326084E-4	1.3862684E-4
A_24_P80633	CTNNA1	1,6518188	5.779992E-4	3.5286095E-4
A_32_P531428	VMAC	1,6468189	9.7645127E-4	7.7713816E-4
A_24_P159312	SLC7A6	1,6463554	0.001155831	0.0010292409
A_24_P382724	TUG1	1,6453303	3.144604E-4	1.3367512E-4
A_23_P396541	TADA2B	1,6435994	1.00626814E-4	1.9597732E-5
A_24_P11965	MRFAP1	1,6428521	9.7920136E-5	1.7576058E-5
A_24_P453259	ATF6	1,642352	0.0010905329	9.3079807E-4
A_24_P916141	DCAF7	1,6416059	4.1740912E-4	2.055445E-4
A_23_P111865	ZSCAN21	1,6396899	0.001155831	0.0010378966
A_23_P166193	EDEM2	1,6390388	3.5323558E-4	1.6206501E-4
A_24_P328819	TRAPPC11	1,6368743	1.10848516E-4	2.366429E-5
A_23_P210995	OSBPL2	1,6366693	3.676868E-4	1.7282656E-4
A_23_P2692	COG3	1,6354042	3.4359135E-4	1.5442308E-4
A_24_P320526	C6orf106	1,6350344	9.897869E-4	7.9516595E-4
A_24_P39759	RNF135	1,6343216	1.471672E-4	3.7756377E-5
A_24_P65121	TTC23	1,6288732	0.0010746553	9.015835E-4
A_24_P341019	TMEM230	1,6281607	0.0013554527	0.0013534412
A_24_P362394	VPS45	1,625835	0.0012546513	0.001200612

A_24_P484797	CIDECP	1,6256526	0.001261567	0.0012095923
A_24_P40594	HOMEZ	1,6244389	6.7227555E-4	4.4718845E-4
A_23_P213000	WDR1	1,6236742	4.6855904E-4	2.597256E-4
A_23_P251767	BANP	1,6205798	6.287135E-4	4.0265924E-4
A_23_P4007	FXR2	1,6188549	0.0013554527	0.0013554527
A_24_P307626	ATP1A4	1,6187612	0.0010380717	8.592279E-4
A_23_P84256	MAP2K5	1,6174879	0.0012981272	0.0012753889
A_23_P204702	TMBIM6	1,616638	0.0011960202	0.0011034444
A_24_P71021	GNB1	1,6153026	4.609739E-4	2.5140305E-4
A_23_P166135	XRN2	1,6116837	1.1580153E-4	2.5155388E-5
A_32_P188178	TRIP12	1,6113429	7.2878075E-4	5.063252E-4
A_32_P27046	CHGA	1,6107023	0.0012759074	0.0012320913
A_24_P193570	CNOT1	1,6100684	1.5313512E-4	4.171557E-5
A_24_P92640	LOC100128288	1,6096517	0.0012871044	0.0012485395
A_23_P111188	ZBTB22	1,6079589	8.9960964E-4	6.9913483E-4
A_23_P258931	ZNF623	1,6079545	0.001086326	9.195119E-4
A_24_P70993	CD99	1,6068523	0.001155831	0.0010337102
A_24_P410363	EGLN1	1,6043928	0.0013486039	0.0013385019
A_23_P27334	ZBTB14	1,6037934	9.923554E-4	7.990877E-4
A_23_P154786	MAP1LC3A	1,6032311	5.7062594E-4	3.4419796E-4
A_24_P103922	CTBP2	1,6031243	2.111384E-4	7.06638E-5
A_23_P205255	WDR20	1,5993565	2.4302263E-4	8.7595676E-5
A_24_P98249	TACC1	1,5993267	3.4080376E-4	1.5070927E-4
A_23_P142776	EIF3F	1,5991976	7.902579E-4	5.8221014E-4
A_23_P205296	EXD2	1,5984857	0.0010380717	8.573378E-4
A_23_P153783	USF2	1,5983433	8.529834E-5	1.2628166E-5
A_23_P37897	C16orf62	1,5976621	2.4777706E-4	9.161819E-5
A_23_P20248	MAP2K1	1,5976027	9.951883E-4	8.032324E-4
A_24_P378788	SLC35E1	1,5966094	2.9441714E-4	1.2074411E-4
A_23_P84565	POLR3D	1,5950434	8.5538055E-4	6.510192E-4
A_23_P12849	FBXO18	1,5925415	1.2542967E-4	2.8891103E-5
A_23_P207620	ANKFY1	1,5922011	1.5313512E-4	4.115837E-5
A_23_P137715	POGK	1,590145	7.09686E-4	4.887258E-4
A_23_P35343	ZNF248	1,5880202	0.0012309954	0.0011631169
A_23_P152272	RNPS1	1,5870935	0.0012453897	0.0011894171
A_24_P206047	SLC25A4	1,5864114	0.001016408	8.24166E-4
A_24_P120827	HGSNAT	1,5849605	2.4819013E-4	9.295511E-5
A_23_P24193	XPNPEP1	1,5816466	1.4144073E-4	3.443314E-5
A_23_P407012	CSF1	1,5784163	4.609739E-4	2.519989E-4
A_24_P766577	RNPS1	1,5782063	0.0011638909	0.0010535709
A_24_P143758	TPT1-AS1	1,5771393	9.7645127E-4	7.7703584E-4
A_23_P120442	NCOA3	1,5763634	6.6441024E-4	4.3920754E-4
A_23_P416242	RNF166	1,5759966	8.539912E-4	6.4449146E-4
A_23_P80156	PRMT2	1,5723153	5.987173E-4	3.7904747E-4
A_24_P49349	RABGAP1L	1,5699321	0.0011880514	0.0010857099
A_23_P133799	KLC4	1,5693338	0.0011863862	0.0010819664
A_23_P37076	BAG5	1,5678136	2.4777706E-4	9.164485E-5
A_23_P129704	TERF2IP	1,5670497	9.7752294E-5	1.7058957E-5

A_24_P185347	MBD1	1,5661194	3.650401E-4	1.7089891E-4
A_23_P402952	CTAGE15	1,5641209	3.4034465E-4	1.4977714E-4
A_23_P94636	RC3H2	1,5633167	3.6444032E-4	1.6993565E-4
A_24_P194755	MUM1	1,5613483	0.0011960202	0.0010997667
A_23_P113311	P4HTM	1,560372	0.0012085402	0.001120276
A_23_P144054	PRKCD	1,5591323	8.5538055E-4	6.509517E-4
A_24_P692198	GOT2	1,5582899	3.8892985E-4	1.8645327E-4
A_24_P394368	WDR19	1,5576041	6.4699404E-4	4.1921338E-4
A_24_P79755	AKR1A1	1,555571	8.1277266E-4	6.0425233E-4
A_32_P169574	OTUD5	1,5552272	5.987173E-4	3.7820358E-4
A_23_P29036	IFNGR2	1,5533623	7.6622394E-4	5.524274E-4
A_23_P8416	GALNT11	1,5530868	1.5266582E-4	4.0024745E-5
A_23_P39813	TTC31	1,5501318	3.1874437E-4	1.360931E-4
A_24_P373312	NFATC3	1,5497417	0.0010380717	8.565439E-4
A_23_P71821	PBX3	1,5478382	8.813992E-4	6.767297E-4
A_24_P364363	TNFRSF1A	1,5468543	9.897869E-4	7.948902E-4
A_23_P501339	CDIPT	1,5462148	0.001098584	9.40174E-4
A_32_P144708	CTBP2	1,5448623	3.364182E-4	1.4659186E-4
A_24_P202139	METTL9	1,5424166	0.0012759074	0.0012352886
A_24_P415601	RNH1	1,5415772	0.0013374251	0.0013249024
A_24_P226554	ACTB	1,5410713	6.5420143E-4	4.3049938E-4
A_24_P402106	ANKMY1	1,5359099	6.729901E-4	4.5118062E-4
A_23_P57643	TPRA1	1,5342983	0.0011460055	9.979261E-4
A_24_P62237	CERK	1,5307102	4.2198863E-4	2.163273E-4
A_23_P436117	HMG20A	1,5301838	5.7062594E-4	3.4571625E-4
A_23_P100103	VPS39	1,5294685	6.9625E-5	9.648408E-6
A_24_P243278	DAP	1,5283608	4.5521953E-4	2.4210177E-4
A_23_P77562	TMEM219	1,5272815	5.433107E-4	3.2456202E-4
A_23_P151653	APEX1	1,5211177	0.0011082354	9.5673505E-4
A_23_P88817	MLYCD	1,5206157	8.259023E-4	6.1710674E-4
A_32_P64570	KANK1	1,520615	0.0011673791	0.00106026
A_24_P305223	CTAGE1	1,5202446	0.001288372	0.0012521817
A_24_P186174	PPP6R2	1,5201818	7.634478E-4	5.4899615E-4
A_23_P100795	STAT3	1,520116	0.0012194351	0.0011339874
A_23_P208265	RPS5	1,5186596	0.001155831	0.0010292566
A_32_P447539	HMCN1	1,5179182	3.7209535E-4	1.7698918E-4
A_24_P369154	PPP6C	1,5162009	2.4179059E-4	8.648315E-5
A_24_P171345	PUM1	1,5153754	8.7635155E-4	6.712131E-4
A_24_P849801	RPL22	1,5124099	7.575678E-4	5.433492E-4
A_23_P131176	TMEM185B	1,5108728	6.5268413E-4	4.2432538E-4
A_23_P75749	GLYAT	1,5098917	0.001155831	0.0010326097
A_24_P24092	ZNF181	1,5067663	4.517699E-4	2.3857513E-4
A_24_P63727	STK38	1,5059849	0.0012085402	0.0011201852
A_23_P303203	CHMP7	1,5045445	9.0319983E-4	7.0361636E-4
A_23_P140602	NGRN	1,5036159	0.0011561047	0.0010435253
A_23_P29517	TFG	1,503106	7.34337E-5	1.0313722E-5
A_24_P201531	ARCN1	1,5026165	7.2892924E-4	5.077934E-4
A_24_P238744	POTEM	1,5001304	7.088077E-4	4.8581202E-4

Table S4: Gene ontology analysis for classification of the genes upregulated in PT based on the biological process

GO ID	GO Term	Count	p-Value	Genes	Fold Enrichment
46835	carbohydrate phosphorylation	3	0.0031	GCK, GNPTAB, HK2	34.51
6887	exocytosis	6	0.0081	STX2, NLGN1, CRCP, EXOC5, TXLNA, RAB27A	4.80
33554	cellular response to stress	14	0.0082	RAD23B, BRCC3, TNIK, NEK1, SMC5, CHEK1, PTPN11, LOC100133315, FANCM, DHRS2, RIF1, BAX, POLQ, MAP2K6	2.27
16192	vesicle-mediated transport	14	0.0094	KDELR2, STX2, NLGN1, CRCP, TXLNA, MON2, MARCH3, HOOK3, KRAS, FNBP1L, MCFD2, EXOC5, SH3D19, RAB27A	2.23
8104	protein localization	21	0.0012	KDELR2, STX2, NLGN1, ICMT, MON2, TIMM8B, HOOK3, EZR, CHML, GNPTAB, ANK3, ATG4A, BAX, MCFD2, NUP50, SRP72, EXOC5, NUP35, PEX10, NSF, RAB27A	2.19
7049	cell cycle	18	0.0042	TAF2, CKAP2, HAUS6, PDS5B, LIN9, DBF4, NEK1, DTYMK, E2F8, CHEK1, EML4, PA2G4, RIF1, SEPT7P2, NCAPG2, ZWILCH, ASPM, MAP2K6	2.13

Table S5: Gene ontology analysis for classification of the genes downregulated in PT based on the biological process

GO ID	GO Term	Count	p-Value	Genes	Fold Enrichment
6890	retrograde vesicle-mediated transport, Golgi to ER	6	0.0001	COG3, COPA, ARF1, ARCN1, RAB6B, TAPBP	12.8
6901	vesicle coating	4	0.0041	COPA, SNAP91, ARF1, ARCN1	11.95
51650	establishment of vesicle localization	5	0.0059	COPA, ARF1, PSEN1, MAP2K1, ARCN1	6.79
34976	response to endoplasmic reticulum stress	5	0.0066	ATF6, HERPUD1, AARS, TP53, HSPA5	6.59
31669	cellular response to nutrient levels	6	0.0028	PSEN1, MAP1LC3A, NUAK2, TP53, HSPA5, USF2	6.11
16050	vesicle organization	6	0.0058	COPA, RAB8A, SNAP91, ARF1, ARCN1, SORT1	5.17
6401	RNA catabolic process	7	0.0032	ZFP36, RNH1, RNPS1, HSPA1A, CASC3, DEDD2, XRN2	4.82
51098	regulation of binding	10	0.0072	KDM1A, MYD88, PSEN1, NCOA3, SKI, TNKS, FKBP1A, EGLN1, APEX1, PRKCD	2.93
1701	in utero embryonic development	11	0.0061	HES1, KDM1A, WDTC1, PCGF2, CEBPB, SYVN1, NDEL1, MYO1E, TP53, EGLN1, JUNB	2.8
46907	intracellular transport	35	0.000004	ARSB, COPA, SNX9, SNX15, CHMP7, AP3S2, PEX5, FTH1, TAPBP, WDR19, SQSTM1, DUSP16, EXOC4, PUM1, RAB6B, SEC24C, KDELR1, DYNC1I1, ARHGEF2, RAB8A, ACTC1, SCAMP2, MAP2K1, VPS45, MYO1E, TP53, M6PR, COG3, CBLB, NDEL1, PSEN1, ARF1, ARCN1, SORT1, ATP7B	2.39
60548	negative regulation of cell death	19	0.0012	CEBPB, SYVN1, SLC25A4, NUAK2, TMBIM6, AARS, TP53, CBX4, HSPA1A, PPT1, TP73, PCGF2, MYD88, PSEN1, SQSTM1, RHOA, SORT1, HSPA5, TNFAIP3	2.36
70727	cellular macromolecule localization	20	0.0024	SNX9, COPA, ARHGEF2, SNX15, VPS45, AP3S2, TP53, PEX5, TERF2IP, CASC3, COG3, CBLB,	2.16

				WDR19, DUSP16, ARCN1, EXOC4, SORT1, TNKS, SEC24C, KDELR1	
45892	negative regulation of transcription, DNA-dependent	17	0.0062	PPARA, GLIS3, WDTC1, CTBP2, PKIG, TP53, TRIM27, CBX4, SKI, STAT3, HES1, KDM1A, PCGF2, BCL6B, SIK1, HDAC8, RASD1	2.14
16192	vesicle-mediated transport	27	0.0005	COPA, SNAP91, CHMP7, AP3S2, PPT1, TXLNA, ITSN2, FTH1, TAPBP, WDR19, SQSTM1, NECAP2, EXOC4, PUM1, RAB6B, SEC24C, KDELR1, RAB8A, SCAMP2, VPS45, M6PR, ELMO2, COG3, PSEN1, ARF1, ARCN1, SORT1	2.1
34613	cellular protein localization	19	0.0049	SNX9, COPA, ARHGEF2, SNX15, VPS45, AP3S2, TP53, PEX5, TERF2IP, COG3, CBLB, WDR19, DUSP16, ARCN1, EXOC4, SORT1, TNKS, SEC24C, KDELR1	2.07
51254	positive regulation of RNA metabolic process	22	0.0026	KLF6, PPARA, DVL3, GLIS3, CEBPB, MAP2K1, TP53, ELK1, USF2, JUNB, TP73, STAT3, ARHGEF11, HES1, ATF6, TNFRSF1A, NCOA3, PAX9, ZSCAN21, SQSTM1, CSRNP1, NFATC3	2.05
6915	apoptosis	27	0.00010	PHLPP1, TNFRSF21, NUAK2, PPT1, BAG5, TNFRSF1A, MOAP1, TSC22D3, SQSTM1, DAP, TRAF7, DEDD2, ARHGEF2, YARS, ACTC1, TMBIM6, TP53, ELMO2, TP73, ARHGEF11, VDAC1, PSEN1, CSRNP1, PDCD6IP, SIAH2, TNFAIP3, GADD45B	2.01

Table S6: Enriched biological pathways captured by all differentially expressed genes in PT-ECFCs based on Genespring Pathways database: WikiPathway, KEGG. Pathways with enrichment p-value ≤ 0.01 are shown here. Pathways involving MAPK pathways modulation are highlighted in bold.

**WikiPathway**	**Count**	**p-value**
Hs_miR-targeted_genes_in_lymphocytes_-_TarBase_WP2004_82897	28	3,69E-07
Hs_Lymphocyte_TarBase_WP2004_46264	28	5,46E-07
Hs_miR-targeted_genes_in_muscle_cell_-_TarBase_WP2005_82898	24	1,02E-06
Hs_Oncostatin_M_Signaling_Pathway_WP2374_54197	10	1,80E-06
Hs_Muscle_cell_TarBase_WP2005_44926	23	3,85E-06
Hs_miR-targeted_genes_in_epithelium_-_TarBase_WP2002_82943	19	1,90E-05
Hs_Epithelium_TarBase_WP2002_45243	19	2,45E-05
Hs_trans-Golgi_Network_Vesicle_Budding_WP3578_83468	9	2,70E-05
Hs_DNA_Damage_Response_(only_ATM_dependent)_WP710_79974	11	7,21E-05
Hs_IL-5_signaling_pathway_WP127_44626	6	1,18E-04
Hs_Wnt_Signaling_Pathway_and_Pluripotency_WP399_79474	10	1,23E-04
Hs_miR-targeted_genes_in_leukocytes_-_TarBase_WP2003_82960	11	1,77E-04
Hs_RNA_Polymerase_I,_RNA_Polymerase_III,_and_Mitochondrial Transcription_WP1905_83386	10	3,09E-04
Hs_Insulin_Signaling_WP481_82731	12	3,78E-04
**Hs_MAPK_Cascade_WP422_38880**	**5**	**4,96E-04**
Hs_Cytosolic_sensors_of_pathogen-associated_DNA_WP2794_83421	7	5,17E-04
Hs_BDNF_signaling_pathway_WP2380_79953	11	5,61E-04
Hs_Leukocyte_TarBase_WP2003_44886	10	6,89E-04
Hs_RIG-I-MDA5_mediated_induction_of_IFN-alpha-beta_pathways_WP1904_83061	7	7,62E-04
Hs_Structural_Pathway_of_Interleukin_1_(IL-1)_WP2637_80029	6	8,18E-04
Hs_Nuclear_Receptors_Meta-Pathway_WP2882_83040	17	8,32E-04
Hs_AGE-RAGE_pathway_WP2324_53381	7	8,36E-04
Hs_SUMOylation_of_DNA_damage_response_and_repair_proteins_ WP3327_83291	7	1,00E-03
Hs_Regulation_of_toll-like_receptor_signaling_pathway_WP1449_45049	10	0,001246077
Hs_EGF-EGFR_Signaling_Pathway_WP437_79266	11	0,001474413
Hs_Interferon_type_I_WP585_47017	6	0,001531327
**Hs_MAPK_Signaling_Pathway_WP382_79951**	**11**	**0,001967802**
Hs_O-linked_glycosylation_WP3315_83262	8	0,002035042
Hs_Signaling_by_VEGF_WP1919_83276	8	0,002035042
Hs_Signaling_by_SCF-KIT_WP2713_83115	5	0,002227336
**Hs_Regulation_of_Actin_Cytoskeleton_WP51_79977**	**10**	**0,002591429**
Hs_IL-6_signaling_pathway_WP364_44627	5	0,002772101
Hs_Leptin_signaling_pathway_WP2034_44631	6	0,0028723
Hs_Signaling_Pathways_in_Glioblastoma_WP2261_81197	7	0,002969615
Hs_TSH_signaling_pathway_WP2032_44635	6	0,003953194
Hs_Wnt_Signaling_Pathway_WP428_79854	6	0,004264917
Hs_Metabolism_of_carbohydrates_WP1848_83255	9	0,005261387
Hs_Parkin-Ubiquitin_Proteasomal_System_pathway_WP2359_53587	6	0,005309053
Hs_Integrated_Breast_Cancer_Pathway_WP1984_82941	10	0,005342348

Hs_G_Protein_Signaling_Pathways_WP35_79952	7	0,005616032
Hs_Oxidative_Stress_Induced_Senescence_WP3404_83222	7	0,005616032
Hs_TNF_alpha_Signaling_Pathway_WP231_81070	7	0,005616032
Hs_Mitotic_Prophase_WP2654_83248	7	0,0059546
Hs_TGF_beta_Signaling_Pathway_WP366_45152	8	0,00602505
Hs_IL-1_signaling_pathway_WP195_44621	5	0,006455536
Hs_mRNA_Processing_WP411_79980	8	0,008858873
Hs_Processing_of_Capped_Intron-Containing_Pre-mRNA_WP1889_83367	9	0,008889845

**KEGG**	**Count**	**p-value**
**MAPK signaling pathway**	**16**	**3.3950486E-4**
Glycolysis / Gluconeogenesis	5	0.017449163
Pathways in cancer	17	0.0018220696

Table S7: Primer sequences used in ChIP experiment. All primer sets worked under identical quantitative PCR cycling conditions. Some of the primers used in this study are described in previous study: Human β-actin promoter (-204 ~ -59)[13].

Gene	Accession		Sequences (5'-3')	Length (bp)	Amplicon (bp)
Human MAP2K6 Promoter 1 (-594 ~ -410)	NM _002758	for	CAGACCTTCTCGACAAGCCA	20	185
		rev	CGAGATAACTGGGTGCGACA	20	
Human MAP2K6 Promoter 2 (-437 ~ -329)	NM _002758	for	GCAATCTTTGTCGCACCCAG	20	109
		rev	GAAGGCCGCTGGGTAATGTA	20	
Human MAP2K6 Promoter 3 (-112 ~ +66)	NM _002758	for	TTGCATGAAGATTGCACGCC	20	179
		rev	ATGCATGCACAACCCTTTGC	20	
Human MAP2K6 Promoter 4 (+92 ~ +200)	NM _002758	for	GCCTGGCGAGTGCATTTTTA	20	109
		rev	AAGCCCTGCCTTACAGGAAC	20	
Human MAP2K6 Promoter 5 (+182~ +297)	NM _002758	for	TTCCTGTAAGGCAGGGCTTG	20	116
		rev	CCAACCTGCTCTGTCTCTCC	20	
Human MAP2K6 Promoter 6 (+299 ~ +492)	NM _002758	for	AGCGTCTAAATGTGCAAGGCT	21	194
		rev	ACCGCAACGGTCTATTTCCC	20	
Human MAP2K6 Promoter 7 (+470 ~ +615)	NM _002758	for	TGGGGGAAATAGACCGTTGC	20	146
		rev	GAGCAAGACTCGTCTGCTTTT	21	
Human MAP2K6 Promoter 8 (+707 ~ +792)	NM _002758	for	GGGGGATGCTTTGCATCTTG	20	86
		rev	AACAGTGCAGTCCCTTCACAC	21	
Human MAP2K6 Enhancer 1 (+2459~ +2614)	NM _002758	for	CTCTGGAAGCTGGAAGGACTG	21	156
		rev	AACTCAACCCCAGACAGCTAA	21	
Human MAP2K6 Enhancer 2 (+3005 ~ +3196)	NM _002758	for	GCAGAGTGGTAGGAAAGGTCT	21	192
		rev	GTGTCCTGAGATGTACGGGG	20b	
Human MAP2K6 Enhancer 3 (+9052 ~ +9214)	NM _002758	for	TGTCAACGTGAAAGCCTCCC	20	163
		rev	TCCACGATGTAAGCGACTCT	20	
Human MAP2K6 Enhancer 4 (+15802 ~ +15953)	NM _002758	for	AGGGAGCACGCAGTCTAATG	20	152
		rev	GTGAGTCGCTCTACATCCCC	20	
Human Chromosome 5 (Control)		for	GGAGGTGGGGAAGTCAGGAA	20	163
		rev	GATCATCCGTGGCTTGAGAT	20	

Supplemental References

1. Vassallo PF, Simoncini S, Ligi I, Chateau A-L, Bachelier R, Robert S, Morere J, Fernandez S, Guillet B, Marcelli M, Tellier E, Pascal A, Simeoni U, Anfosso F, Magdinier F, Dignat-George F, Sabatier F. Accelerated senescence of cord blood endothelial progenitor cells in premature neonates is driven by SIRT1 decreased expression. *Blood*. 2014;123:2116–2126.

2. Freund A, Patil CK, Campisi J. p38MAPK is a novel DNA damage response-independent regulator of the senescence-associated secretory phenotype. *EMBO J*. 2011;30:1536–1548.

3. Maas SLN, De Vrij J, Broekman MLD. Quantification and size-profiling of extracellular vesicles using tunable resistive pulse sensing. *J Vis Exp JoVE*. 2014;e51623.

4. Robert S, Lacroix R, Poncelet P, Harhouri K, Bouriche T, Judicone C, Wischhusen J, Arnaud L, Dignat-George F. High-sensitivity flow cytometry provides access to standardized measurement of small-size microparticles--brief report. *Arterioscler Thromb Vasc Biol*. 2012;32:1054–1058.

5. Simoncini S, Njock M-S, Robert S, Camoin-Jau L, Sampol J, Harlé J-R, Nguyen C, Dignat-George F, Anfosso F. TRAIL/Apo2L mediates the release of procoagulant endothelial microparticles induced by thrombin in vitro: a potential mechanism linking inflammation and coagulation. *Circ Res*. 2009;104:943–951.

6. Lacroix R, Robert S, Poncelet P, Kasthuri RS, Key NS, Dignat-George F, ISTH SSC Workshop. Standardization of platelet-derived microparticle enumeration by flow cytometry with calibrated beads: results of the International Society on Thrombosis and Haemostasis SSC Collaborative workshop. *J Thromb Haemost JTH*. 2010;8:2571–2574.

7. Thuny F, Textoris J, Amara AB, Filali AE, Capo C, Habib G, Raoult D, Mege J-L. The gene expression analysis of blood reveals S100A11 and AQP9 as potential biomarkers of infective endocarditis. *PloS One*. 2012;7:e31490.

8. Bolstad BM, Irizarry RA, Astrand M, Speed TP. A comparison of normalization methods for high density oligonucleotide array data based on variance and bias. *Bioinforma Oxf Engl*. 2003;19:185–193.

9. Magdinier F, Wolffe AP. Selective association of the methyl-CpG binding protein MBD2 with the silent p14/p16 locus in human neoplasia. *Proc Natl Acad Sci U S A*. 2001;98:4990–4995.

10. Jaffe EA, Nachman RL, Becker CG, Minick CR. Culture of human endothelial cells derived from umbilical veins. Identification by morphologic and immunologic criteria. *J Clin Invest*. 1973;52:2745–2756.

11. Akeson AL, Woods CW. A fluorometric assay for the quantitation of cell adherence to endothelial cells. *J Immunol Methods*. 1993;163:181–185.

12. Vaporciyan AA, Jones ML, Ward PA. Rapid analysis of leukocyte-endothelial adhesion. *J Immunol Methods*. 1993;159:93–100.

13. Zhou S, Chen H-Z, Wan Y-Z, Zhang Q-J, Wei Y-S, Huang S, Liu J-J, Lu Y-B, Zhang Z-Q, Yang R-F, Zhang R, Cai H, Liu D-P, Liang C-C. Repression of P66Shc expression by SIRT1 contributes to the prevention of hyperglycemia-induced endothelial dysfunction. *Circ Res*. 2011;109:639–648.
